# A novel method of differential gene expression analysis using multiple cDNA libraries applied to the identification of tumour endothelial genes

**DOI:** 10.1186/1471-2164-9-153

**Published:** 2008-04-07

**Authors:** John MJ Herbert, Dov Stekel, Sharon Sanderson, Victoria L Heath, Roy Bicknell

**Affiliations:** 1 Cancer Research UK Angiogenesis Group, Institute for Biomedical Research, University of Birmingham Medical School, Edgbaston, BIRMINGHAM, B15 2TT, UK; 2 Centre for Systems Biology, School of Biosciences, University of Birmingham, Edgbaston, BIRMINGHAM, B15 2TT, UK; 3 Cancer Research UK, Institute of Molecular Medicine, University of Oxford, John Radcliffe Hospital, OXFORD, OX3 9DS, UK

## Abstract

**Background:**

In this study, differential gene expression analysis using complementary DNA (cDNA) libraries has been improved. Firstly by the introduction of an accurate method of assigning Expressed Sequence Tags (ESTs) to genes and secondly, by using a novel likelihood ratio statistical scoring of differential gene expression between two pools of cDNA libraries. These methods were applied to the latest available cell line and bulk tissue cDNA libraries in a two-step screen to predict novel tumour endothelial markers. Initially, endothelial cell lines were in silico subtracted from non-endothelial cell lines to identify endothelial genes. Subsequently, a second bulk tumour versus normal tissue subtraction was employed to predict tumour endothelial markers.

**Results:**

From an endothelial cDNA library analysis, 431 genes were significantly up regulated in endothelial cells with a False Discovery Rate adjusted q-value of 0.01 or less and 104 of these were expressed only in endothelial cells. Combining the cDNA library data with the latest Serial Analysis of Gene Expression (SAGE) library data derived a complete list of 459 genes preferentially expressed in endothelium. 27 genes were predicted tumour endothelial markers in multiple tissues based on the second bulk tissue screen.

**Conclusion:**

This approach represents a significant advance on earlier work in its ability to accurately assign an EST to a gene, statistically measure differential expression between two pools of cDNA libraries and predict putative tumour endothelial markers before entering the laboratory. These methods are of value and available  to researchers that are interested in the analysis of transcriptomic data.

## Background

### Study aim

The growth and survival of tumours is dependent on their ability to obtain a blood supply and damage inflicted on the tumour endothelium has been shown to effectively eradicate tumours [1]. It follows that the discovery of widely expressed tumour endothelial markers promises much clinical benefit [2]. The aim of this study was to apply novel bioinformatic methods to the latest public expression data repositories, with an emphasis on cDNA library analysis, to create an up to date list of putative endothelial genes and to predict tumour endothelial markers that are potential anti-cancer targets.

Previous studies [3-15] have employed cDNA or SAGE libraries to predict the transcriptional profiles of tissues of interest that were subsequently confirmed by experimental analysis. Our analysis [8] employed a cDNA subtractive Basic Local Alignment Search Tool (BLAST) [16], algorithm to predict endothelial specific genes. This approach required cross referencing of the results to SAGE libraries to confidently predict endothelial expression due to a large number of false positives associated with the BLAST method of EST to gene assignment used. In the study by Ho et al. 2003 [7], Unigene's Digital Differential Display (DDD) tool was employed to predict endothelial genes, which is reliant on Unigene clusters. DDD requires at least 1000 EST sequences from a cDNA library to be clustered into Unigene clusters for valid statistical analysis and can measure statistical significance accurately between only two libraries [15]. This 1000 sequence limit of DDD can remove small, but often potentially relevant, cDNA libraries from an analysis.

### Improving cDNA library analysis

This study aimed to improve both the statistical analysis and EST to gene assignment methods used in subtractive in silico cDNA differential gene expression analyses. To eliminate the cDNA library false positive discovery rate of the Huminiecki and Bicknell 2000 [8] study, EST to gene assignment was improved by combining human genome position of sequences with a BLAST database search. This eliminated all EST to gene assignment ambiguity.

In contrast to microarray gene expression data that produces continuous measures, EST data is in the form of discrete counts and the methods for finding differentially expressed genes are necessarily different from those used for microarrays [17]. Methods for EST analysis include the use of a Poisson model for the EST counts to derive a test statistic [15,18], a multinomial model leading to a traditional Chi Squared test [19], or a test conditioned on a constant total EST count using a hypergeometric or binomial distribution [19]. In contrast, the SAGEmap algorithm [20] computes a posterior distribution for a fold ratio. A disadvantage of the traditional Chi Squared test [19] is that it is unreliable when the EST counts are low. Here, we describe a new likelihood ratio test that is an extension of the method of Stekel et al. 2000 [15], that instead of identifying differentially expressed genes across a set of different libraries, will identify genes that are differentially expressed between two groups of libraries; within the groups the libraries should be the same, but between groups they may be different. The method removes the need for the 1000 Unigene cluster limit of the DDD tool, enables any size cDNA library to be analysed and accurately determines differential expression across more than two cDNA libraries. We also compare the results of our method with the methods of Susko and Roger [19] and the SAGEmap algorithm [20].

### In silico Tumour Endothelial Marker prediction

A two-step analysis was performed to predict tumour endothelial markers (TEMs). The first stage identified endothelial genes by comparing the expression patterns of genes between endothelial and non-endothelial cell lines. The second stage involved a comparison of bulk tumour and bulk normal cDNA libraries to identify genes up regulated in tumours. Putative TEMs are genes that were both endothelial and preferentially expressed in tumours.

## Results

### Development of an algorithm for EST to gene assignment

A new algorithm for assigning an EST to a gene has been developed that takes advantage of the almost complete human genome and combines it with a BLAST database search to achieve an accurate result. Initially, two EST pools and all Reference sequence project (Refseq) [21] mRNA sequences were aligned to the human genome using the BLAST like alignment tool (BLAT) [22]. BLAT was used in preference to BLAST for genome alignments because of its superior speed. Sequences occupying an ambiguous position in the genome were removed. The aligned sequences were then collected into Perl data structures and a simple custom-clustering algorithm (Jake cluster) assigned each EST to a gene or gene prediction based on their overlapping genome position. In the BLAST database search, each EST was BLAST searched against a Refseq database containing human mRNA and gene predictions. Only the best mRNA hit from the BLAST database search was assigned to an EST and this was regardless of the e-value result. The results of BLAT genome alignments and BLAST database search were then cross-referenced and accurate EST to gene assignment was made based on the following decision tree:

1) If genome BLAT mapping and BLAST database results agreed, then that gene was assigned to the EST regardless of e-value for the database search.

2) If the results disagreed, then the BLAST result alone was accepted if the alignment was of high quality, >= 92% identity with an alignment length of >= 100 bases.

**Figure 1:**
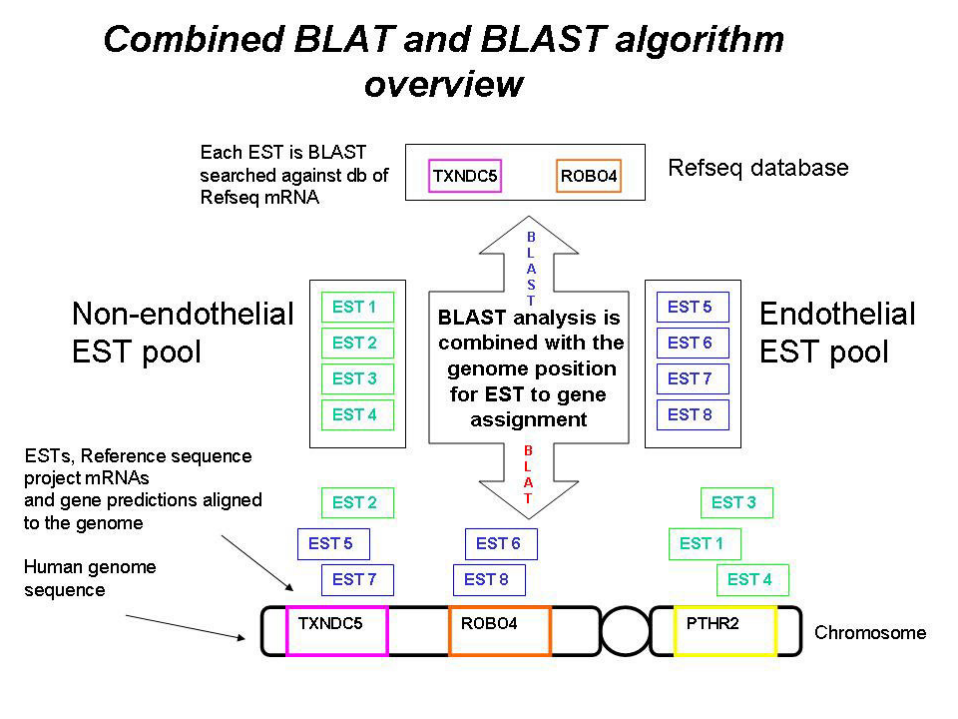
**An overview of the EST to gene assignment process**. Each EST sequence is BLAST searched against a Refseq mRNA database and the best mRNA is assigned that EST. In tandem, a mapping of all ESTs and Refseq mRNA to the human genome assigns ESTs to genes based on genome position. A decision tree makes the final assignment based on the quality of alignment and agreement between the two methods. If the genome position and BLAST result agree, the EST is assigned, if they do not agree but the BLAST result is of high quality (> 92% and > 100 bp alignment) the EST is also assigned. For any other result the EST is removed from the analysis.

A pictorial representation of the analysis is shown in Figure 1. The approach was able to assign ESTs to a gene even when the single pass cDNA sequencing of an EST was of low quality. Thus, first finding an unambiguous position in the genome that overlaps with a gene and then searching with BLAST to find the best gene, it was able to assign an EST to a gene. Further, using a high quality BLAST alignment alone for the assignment gives this approach the ability to also assign a gene that lies in a gap in the human genome sequence.

### Validation of the EST to gene assignment algorithm

The results of the BLAST subtraction method used in our previous work [8] were compared to those of the algorithm developed here. Using a custom relational database developed in house, cDNA libraries were collected and divided into 2 pools (endothelial and non-endothelial cells respectively) and formatted into BLAST databases. The same data were used in this experiment as used in the earlier study because the EST to gene BLAST protocol was dependent on an e-value [23]. The e-value was optimised in the earlier work for predictive capacity by performing trial runs. E-values are dependent on the size of a BLAST database and so it was important to use the same data. This was possible for the endothelial cell lines as the exact 11,117 ESTs were collected, however, for the non-endothelial pool the EST count had increased from 173,137 to 178,653 as a result of further EST sequencing. The expected value for the non-endothelial pool was kept at 10e-20 as the larger pool size made the e-value more stringent and less likely to deliver false positive hits.

Although there was a good agreement between the old BLAST and combined method EST assignments for some genes, a problem with searching mRNA queries against an EST database is that any EST is able to hit more than one gene using an e-value cut-off. In reality this is not possible, as an EST is derived from a single transcript derived from a single gene. Table 1 shows the number of EST sequences that hit more than one gene for both EST to gene assignment methods. From the endothelial pool using the earlier [8] method there were 5,228 from the 11,117 ESTs that were assigned to more than one gene using an e-value cut-off of 10e-30. The ambiguous assignment means that it is not possible to know which EST to gene assignment was correct without manual inspection and as such failed. Assuming the remaining 5,889 ESTs that hit only one gene were correctly assigned, this amounted to a 53% success rate. For the new combined algorithm there were no EST sequences assigned to more than one gene and the success rate for EST to gene assignment from the endothelial pool was 91%.

**Table 1:** A comparison of EST to gene assignment methods

	Endothelial EST pool count	ESTs unambiguously assigned to a gene	ESTs assigned to more than 1 gene	% success rate for the total pool
Huminiecki and Bicknell [8]	11,117	5,889	5,228	53
Method described here	11,117	10,153	0	91

A comparison of the two EST to gene assignment methods using the same data. The new method of EST to gene assignment improved accuracy enabling a higher percentage of ESTs to be unambiguously assigned compared to the Huminiecki and Bicknell method [8].

### Testing of statistical significance

To measure statistical significance of differential gene expression using cDNA libraries there is the DDD tool available at Unigene. This tool employs the Fisher exact test [24] to measure statistical significance (P < 0.05) between two libraries. According to [15] the statistics used by DDD are not valid for measuring statistical significance across multiple but only between two cDNA libraries. A further requirement of DDD is that it is only valid for cDNA libraries that contain at least 1000 sequences collected into Unigene clusters. Several endothelial cell line libraries used in this study contained less than 1000 sequences and comparisons between more than two libraries were required. For these reasons, the DDD tool and Unigene clusters were of no use in this analysis.

The statistics in the analyses used here combine a generalised likelihood ratio test with a False Discovery Rate (FDR) that accounts for the different size of the cDNA library pools. During cDNA library construction, bacterial colonies are picked at random from agar plates for single pass sequencing of the EST insert. This process is random and can be modelled by a Poisson distribution. To derive the appropriate statistical method, two hypotheses were compared with each other. The Null Hypothesis is that there is no difference in gene expression between two cDNA library pools and any differences in gene expression are due to sampling errors from the picking of colonies. Alternatively, the difference in gene expression could be due to a genuine biological effect. The likelihood ratio statistic (R-statistic) is derived by dividing the likelihood of seeing the data under the null hypothesis by the likelihood of seeing the data under the alternative hypothesis.

For the latest endothelial cDNA library data two p-values were generated. The first p-value can be derived from the R-statistic because 2R is asymptotically Chi Square distributed. The second was generated using a randomization method similar to the one used in Stekel et al. 2000. It should be noted that multiple testing on all genes in the human genome and using either of these p-values would result in many false positives. To account for multiple testing errors, a False Discovery Rate adjusted (FDR-adjusted) procedure was employed [25]. A q-value [26] of 0.01 represents 1% false discovery rate and means that 10 in 1000 significantly differentially expressed genes were false positives. A q-value of 0.01 was considered significant in this work. For comparison we have also included Bayesian posterior probabilities as used in the SAGE xProfiler tool [20] and a method from Susko and Roger 2004 [19].

### Application of the statistics to the analysis

Applying the new analysis and statistics to the original data used by [8], 14 genes were predicted significantly endothelial specific and a further 160 (Additional file 1) were significantly up regulated in endothelial cells. Table 2 lists the 14 predicted significantly endothelial specific genes.

**Table 2:** Endothelial specific genes found using the original data

Gene	FDR q-value	Endothelial ESTs	Non-endothelial ESTs
ECSM2	0.0000	9	0
TFPI	0.0000	7	0
MMRN1	0.0000	5	0
TIE1	0.0000	5	0
ACTA1	0.0000	5	0
ECSM1	0.0002	4	0
CD34	0.0002	4	0
BMX	0.0031	3	0
LOC650049	0.0031	3	0
APLN	0.0031	3	0
DUS4L	0.0031	3	0
FABP4	0.0031	3	0
LOC643977	0.0031	3	0
PAQR3	0.0031	3	0

A list of predicted endothelial specific genes using the new EST to gene assignment and statistical methods but with the original cDNA library data from Huminiecki and Bicknell [8]. 14 genes were predicted as significantly endothelial specific. A further 160 genes were predicted as showing significantly upregulated endothelial expression (q-value <= 0.01) (additional file 1) but were not endothelial specific (i.e. had EST hits in the non-endothelial pool). With the new analysis there was no longer a need to cross reference to SAGE libraries for accurate prediction.

It is of interest to compare the 16 predicted endothelial genes listed in table 7 of the original Huminiecki and Bicknell 2000 [8] analysis with those found here. Three of the original 16 genes were no longer predicted as significantly endothelial and Table 3 summarises the results. RAMP2 had no ESTs in either pool; COL4A1 was up regulated in endothelial cells but not to significance with a q-value of 0.5. In contrast, RASIP1 was endothelial specific with a single EST found in the endothelial pool but absent from the non-endothelial pool. However, the q-value of 0.36 was again not statistically significant.

**Table 3:** Comparison of methods with table 7 from Huminiecki and Bicknell (2000)

Gene	FDR q-value	Endothelial ESTs	Non-endothelial ESTs	Original Unigene ID
ECSM2	**0.0000**	9	0	Hs.30089
MMRN1	**0.0000**	5	0	Hs.268107
ECSM1	**0.0002**	4	0	Hs.13957
FABP4	**0.0031**	3	0	Hs.83213
RASIP1	0.3696	1	0	Hs.233955
RAMP2	-	0	0	Hs.155106
VWF	**0.0000**	27	1	Hs.110802
CD93 (ECSM3)	**0.0022**	4	1	Hs.8135
ROBO4 (ECSM4)	**0.0022**	4	1	Hs.111518
CDH5	**0.0022**	4	1	Hs.76206
EDN1	**0.0000**	7	2	Hs.2271
SDPR	**0.0001**	6	2	Hs.26530
PECAM1	**0.0000**	24	5	Hs.78146
EFEMP1	**0.0000**	40	8	Hs.76224
COL4A1	0.5598	4	16	Hs.119129
CTGF	**0.0000**	30	49	Hs.75511

Listing of the genes from Table 7 of our earlier work [8] and how they came out in the new analysis. 13 of the 16 genes were significantly endothelial; however, non-endothelial hits to known endothelial genes showed that the choice of non-endothelial cell lines could be improved. q-values in bold denote a significance threshold of <= 0.01.

Three genes identified as endothelial specific in the original analysis were not found to be so here. ROBO4 hit the EST [GenBank:AA577940] from the library NCI_CGAP_HSC1 that is a flow-sorted and non-normalized bone marrow cDNA library. EST accession [GenBank:AI380234] hit CD93 that is from a B-cell, chronic lymphocytic leukaemia flow-sorted cell line (NCI_CGAP_CLL1), while vWF hit a non-endothelial EST from the NCI_CGAP_Br4 library [GenBank:AA721546]. The last library was prepared from micro-dissected normal breast duct tissue and in view of the extensive literature showing restriction of von Willibrand factor expression to endothelium, is presumably from endothelial contamination of the dissected tissue. In subsequent analyses the non-endothelial pool was refined to exclude such hits.

### Current data with the new algorithm and statistics, experiment 1

Employing the new EST assignment algorithm and the novel statistical method, a similar subtractive screen to Huminiecki and Bicknell 2000 [8] was carried out but this time with the most recent publicly available data. In the earlier 2000 study there were 11,117 endothelial EST sequences. This has now increased to 31,114 and 64% of the currently available endothelial cell data was new. Table 4 lists the endothelial cell libraries used.

**Table 4:** Latest endothelial libraries available at Genbank

New/Original	cDNA library	Count
Original	Stratagene endothelial cell 937223	7173
Original	Aorta endothelial cells, TNF alpha-treated	1908
Original	Aorta endothelial cells	1245
Original	Human endothelial cells, large insert, pCMV expression library	859
Original	Umbilical vein endothelial cells II	404
Original	Human aortic endothelium	20
Original	HDMEC cDNA library	12
Original	Umbilical vein endothelial cells I	9
Original	Human endothelial cell (Y. Mitsui)	3
New	PUAEN2	9382
New	Sugano cDNA library, coronary artery endothelial cell	4707
New	VESEN1	1316
New	VESEN2	1173
New	HEV PCR-select	1049
New	UMVEN2	433
New	Human Endothelial cells	346
New	Sugano cDNA library, umbilical vein endothelial cell	342
New	PUAEN1	326
New	UMVEN1	167
New	CAE	88
New	Human umbilical vein Endothelial Cell cDNA library	48
New	Sugano cDNA library, endothelial cell	28
New	Human umbilical vein cord	15
New	IMS_CAS	15
New	Human umbilical venous cord	12
New	HUVEC cDNA Library	12
New	HUVEC Subtracted Library 1	8
New	Plasmid subtractive library of human umbilical vein endothelial cells (HUVEC) stimulated by lipopolysaccharide	8
New	IMS_CAE	4
New	Homo sapiens umbilical vein	2
Total		31114

Genbank endothelial cDNA libraries that were used in this study. 21 new libraries have been submitted since our 2000 analysis [8]. The 30 combined libraries incorporate 31,114 endothelial ESTs.

In view of aberrant gene expression by carcinoma lines arising from genetic instability and endothelial contamination of libraries isolated by Fluorescence-activated cell sorting (FACS) or micro-dissection, a non-endothelial pool with no carcinoma cell, flow sorted or micro-dissected lines (136,336 ESTs, Additional file 2) was constructed (experiment 1).

Additional file 3, parts 1–3, shows the ranking of results using the different p-value methods and the posterior probability. The top 10 endothelial genes differ greatly between methods and is biologically intuitive with the Chi squared derived q-values rather than with the randomization generated q-values or the Susko and Roger statistics [19]. Of potential concern might be low EST counts giving significant results, where Chi Squared statistics might normally be considered unreliable.

An example is the already validated endothelial gene TEK, which has 4 endothelial ESTs to 0 none-endothelial ESTs. The p-value given by the randomization method, for which the low counts are not a problem, is more significant than that given using the Chi Squared approximation. Since our primary concern is minimizing false positive (Type 1) errors, and the Chi Squared approximation is behaving more conservatively than the randomization method, we can confidently report that these genes are differentially expressed despite the small number of ESTs observed. In comparison, both the posterior probability [20] and Susko and Roger [19] methods both produced an insignificant result for this gene. Additional files 4, 5, 6 and 7 show the full results of experiment 1 ranked using the four different statistical methods. It is of interest to note that the posterior probability score using a target fold difference factor of 2 was the most conservative statistic producing a list of 424 genes with a posterior probability cut-off of >= 0.9. In contrast the randomization generated q-values produced 661 significant genes (q-value <= 0.01), the Susko and Roger method 536 and the Chi Square q-values 554 significant genes. We chose to use the Chi Square q-values in all subsequent analyses as this gave the greatest prediction of validated endothelial genes.

**Figure 2:**
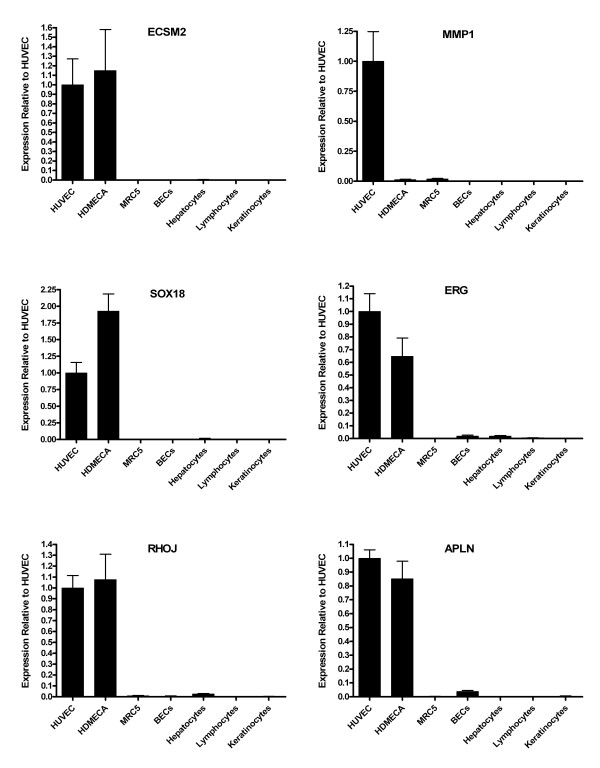
**Real time PCR analysis of randomly chosen endothelial predicted genes across a range of cell types**. Real time PCR was carried out on the predicted endothelial genes ECSM2, MMP1, SOX18, ERG, RHOJ and APLN. The graphs illustrate the power of the bioinformatics models as all genes examined were up regulated or specific to HUVECs and/or HDMECs.

**Figure 3:**
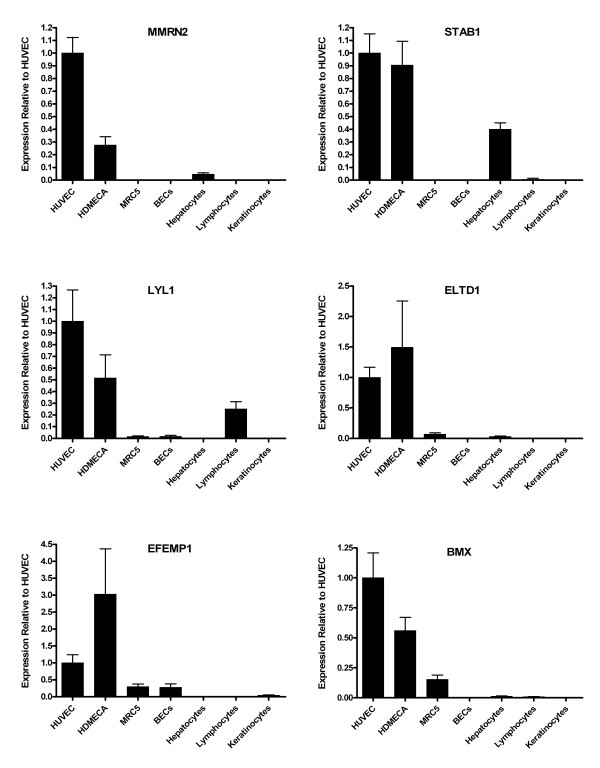
**Real time PCR analysis of randomly chosen endothelial predicted genes across a range of cell types**. Real time PCR was carried out on predicted endothelial genes MMRN2, STAB1, LYL1, ELTD1, EFEMP1 and BMX. The graphs illustrate the power of the bioinformatics models as all genes examined were up regulated or specific to HUVECs and/or HDMECs.

From a cDNA library endothelial subtraction analysis alone (experiment 1) using the Chi Squared generated q-values there were 431 genes (Additional file 8) that were significantly up regulated in endothelial cell lines. Of these, 104 genes showed an endothelial specific profile (Table 5), as transcripts were absent in the non-endothelial pool. The gene with the most significant endothelial specific profile was the metallo-proteinase gene MMP1, a surprising result as literature suggests this gene is widely expressed [27,28]. Based on this literature, it is possible that a fibroblast cell line added to the non-endothelial pool would help to improve the analyses but human lung fibroblasts used for in the Real time PCR in this work and showed no MMP1 expression (Figures 2 and 3). It is also worthy of note that MMP1 is also up regulated in endothelial cells according to SAGE library analysis in experiments 2 and 3. There are currently 13 endothelial SAGE libraries in the public domain and 11 of them express MMP1 at a high level. The average tag count for MMP1 was 216 tags and the median 180 tags. Therefore, although the literature suggests MMP1 is not specific to endothelial cells, the current available SAGE data shows MMP1 to be highly expressed in endothelial cells. The literature also shows MMP1 to have a critical role [29-31]. This analysis also predicted ROBO4, CD93 and VWF as endothelial specific genes.

**Table 5:** Endothelial specific genes from cDNA library analysis with latest data

Gene	FDR q-value	Endothelial ESTs	Non-endothelial ESTs
MMP1	0	203	0
ROBO4	0	130	0
SPARCL1	5.21E-70	97	0
VWF	1.33E-52	73	0
HHIP	6.58E-44	61	0
C9orf26	1.60E-23	33	0
RHOJ	4.68E-22	31	0
BMX	2.45E-21	30	0
ELTD1	1.26E-20	29	0
MMRN1	1.84E-18	26	0
EMCN	5.17E-17	24	0
CDH5	2.75E-16	23	0
SOX7	3.94E-14	20	0
ARHGAP24	1.03E-12	18	0
FGD5, PCDH12	1.03E-12	18	0
CD93	5.37E-12	17	0
ERG, MYCT1	2.64E-11	16	0
FLJ22746	1.28E-10	15	0
SELE	6.56E-10	14	0
ANGPT2, TCF4	3.36E-09	13	0
EDG1	1.68E-08	12	0

This table shows the top 24 genes from the 104 genes in the human genome with the most endothelial specific expression profile predicted by applying the new analysis to the latest cDNA libraries. The other 80 endothelial specific genes are in additional file 8.

### cDNA and SAGE library analysis combined, experiment 2

The SAGE library screen was very similar to the cDNA library approach as it involved comparing two pools of SAGE library cell lines using the SAGEmap xProfiler tool. Although the problem is the same, to find differentially expressed genes, the statistical methods used by Lash et al. [20] differ to this Poisson approach used here as it employs Bayesian statistics. These authors do not reject the null hypothesis that gene expression between two pools of libraries is equal, they estimate the probability that gene expression differs by a fold factor which is dependent on an assumed probability density function f(x). For a full description consult the article on SAGEmap paper [20].

For the second experiment, the data from the cDNA analysis of experiment 1 was combined with SAGE in the same way as the 2000 analysis. The SAGE analysis used the latest endothelial libraries against a pool of normal non-endothelial libraries (Additional file 9). There were 10 endothelial cell line SAGE libraries containing 427,254 tags and 74% of the SAGE library data was new since 2000 and submitted to SAGEmap [20]. Results are presented in Additional file 10 that lists 27 endothelial genes.

### cDNA and SAGE library analysis combined (including carcinoma cell line cDNA data), experiment 3

Although cancer, micro-dissected and sorted libraries in non-endothelial cell lines were thought to contaminate and invalidate the analysis, there exist many of these libraries in the public domain. Thus, to maximise the chance of predicting a comprehensive set of endothelial genes, a final experiment (experiment 3) was performed using non-endothelial libraries that included cancer, micro-dissected and sorted libraries (178,653 ESTs and 733,461 SAGE tags, Additional file 11). The SAGEmap xProfiler analysis was again combined with a cDNA library subtraction. 58 endothelial genes were predicted from this analysis (Additional file 12).

### A comprehensive set of in silico predicted endothelial genes

Combining the results of all three analyses gave a non-redundant list of 459 genes preferentially expressed at a statistically significant level in endothelial cells (Gene symbols in Additional file 13).

### Experimental validation of the endothelial gene prediction

Real time PCR was carried out on predicted endothelial genes to examine the predictive power of the in silico analyses. A random selection of genes was PCR amplified from human umbilical vein endothelial cells (HUVECs), human dermal micro-vascular endothelial cells (HDMECs) and a selection of normal primary, non-endothelial isolates; human lung fibroblasts (MRC-5), human bronchial epithelial cells (HBEC), adult human epidermal keratinocytes, peripheral blood lymphocytes and hepatocytes. Total RNA was extracted and Real time PCR was performed to measure differential expression of these genes between the cell types. Figures 2 and 3 show the power of the bioinformatics models as all genes examined were up regulated or specific to HUVECs and/or HDMECs. Namely, ECSM2, MMP1, SOX18, ERG, RHOJ, APLN, MMRN2, STAB1, LYL1, ELTD1, EFEMP1 and BMX.

### Tumour endothelial marker prediction

Following the prediction of endothelial genes, a second screen was performed to identify genes up regulated in tumours or foetal tissue. Bulk tissue cDNA libraries that contain endothelium were used. All bulk tissue libraries used are in Additional files 14 to 28. The subtraction procedure carried out compared bulk tumour with bulk normal cDNA libraries from the same organ or tissue. The analysis involved six tissues, namely, lung, brain, colon, kidney, prostate and skin. Three foetal tissues (lung, brain and kidney) were also screened since foetal tissues, like tumours, have active angiogenesis. By screening each tissue independently, the analysis was able to identify genes that were putatively up regulated in a tissue specific fashion. Special attention was taken in choosing normal tissue libraries, to ensure that they contained no active angiogenesis (e.g. foetal libraries were avoided for the normal tissue pools). Genes that were both selectively or preferentially expressed in tumour or foetal tissues and preferentially expressed in endothelial cells constituted predicted TEMs. 27 genes were chosen as being potential TEMs based on the specific or/and significant up regulation in multiple tissues (Additional file 29).

## Discussion

### Identifying differentially expressed genes

The identification of cell or tissue specific genes is of ongoing interest to the biologist as such genes often perform a unique function within that cell or tissue type. In the past, tissue specific genes were sought using a range of molecular subtraction techniques employing mRNA/cDNA from the cell type of interest and a putative 'control' cell. Examples of such techniques include subtractive hybridisation, PCR display and PCR select. These approaches have been highly successful but are laborious and expensive. More recent approaches have included selective insertional gene trapping or FACS sorting of cell lineages labelled with GFP in e.g. zebrafish followed by gene chip analysis. Both techniques have been used to identify endothelial genes [32,33], for example in zebrafish the endothelium and precursors were labelled with Fli promoter GFP. Nevertheless, such techniques are still expensive and laborious.

An alternative is to analyse computationally the vast amount of expression data now available in the public domain. We performed such an analysis in 2000 [8] that identified several previously unknown endothelial genes including Robo4, the endothelial Roundabout guidance gene. A critical finding in the earlier analysis was the need to cross reference a cDNA with a SAGE analysis to achieve accurate prediction of expression. A complementary approach by Ho [7] combined cDNA and SAGE library database mining with microarray analysis. Virtual subtraction was carried out on data in the public domain using available tools to identify putative endothelial genes. These genes were then micro-arrayed and probed with RNA samples from a selection of cultured endothelial and non-endothelial cell types. The results of Ho et al. are compared with ours below.

We became aware that these earlier computational techniques could be improved and there are several cogent reasons for repeating such an analysis now. Firstly, the large increase in expression data available now compared to 2000, in particular the number of ESTs in the endothelial libraries has more than doubled. Secondly, following the publication of the human genome, we have developed a new technique that combines a BLAST search with genome BLAT alignments to increase the accuracy of EST to gene assignment. This removes the ambiguity of EST to gene assignment and consequent inaccuracies of the results present in our earlier analysis. Finally, we developed a novel likelihood ratio statistic analysis that can identify differentially expressed genes across multiple cDNA libraries. Using these improvements to cDNA library analysis and the inclusion of the latest SAGE library data, we have derived what we consider to be a near definitive set of endothelial specific genes.

### cDNA library analysis improvements

Our earlier analysis [8] showed that endothelial genes are not reliably predicted using cDNA library analysis alone. Two possible explanations for this were. 1) Computationally, the EST to gene assignment was inaccurate for some genes with the BLAST protocol chosen and 2) a statistical analysis was not applied to the EST counts in order to determine the significance of the differential gene expression.

Repeating our method on the cell lines used in the earlier [8] study validated the new approach. The new analysis proved the critical importance of accurate EST to gene assignment to enable a successful analysis using cDNA libraries alone. The new method produced a successful assignment of 91% of the ESTs compared with 53% for the earlier study. Using the 2007, as apposed to the 2000 data, we have 31,114 assigned endothelial ESTs and a success rate of 94%. It should be noted that this success rate is also dependent on the quality of cDNA libraries, but comparing like for like, the new algorithm improved the accuracy of assignment by 38%. In order to identify differentially expressed genes between two pools of cDNA libraries convincingly, it is essential to employ a rigorous statistical analysis. The method described here makes use of the intrinsic variability associated with cDNA library measurements and represents the most powerful statistical analysis possible associated with that model. We note that the test is more appropriate than a t-test, and more powerful than non-parametric statistics such as the Mann-Whitney test. We also note that differential expression of cDNA libraries can be performed on line at the CGAP and Unigene. However, DDD was not used in these analyses as it does not employ the maximal statistics test and only performs differential expression between cDNA libraries that have at least 1000 EST sequences clustered into Unigene as the analysis is invalid with fewer sequences. In contrast, the likelihood ratio statistics used in these analyses can be applied to cDNA libraries of any size and the EST to gene assignment does not rely on Unigene clusters.

One feature of our test is that it does not take into account genes with EST counts of 0 in all libraries. Although such genes are not observed to be differentially expressed, and so do not contribute to the FDR as no test has been applied to them, it is possible for such genes to be truly differentially expressed but expressed at low levels and so not seen. It would be possible to extend the test we describe to take this possibility into account when testing those genes that are expressed. The likelihood functions for the null and alternative hypotheses, instead of based on an unconditional probability, could condition the probabilities on there being a count of at least 1 in at least one library. This is unlikely to make a material difference to the results.

### Comparison of the results with previous work

It is of interest to compare the results of this analysis with two previous bioinformatic analyses to identify endothelial genes, those of Huminiecki and Bicknell [8] and Ho et al. [7]. In our earlier 2000 study, 16 genes were predicted as endothelial by a combined SAGE and cDNA library analysis. From the 16 genes, 13 were also predicted as significantly endothelial in this study. The three genes that differed between the two analyses were COL4A1, RAMP and RASIP1. In the new analysis RASIP1 was endothelial specific but not to significance, COL4A1 was expressed in both cDNA library pools and RAMP was not expressed in either pool. It is interesting that ECSM2 was the most endothelial specific gene in the Huminiecki and Bicknell [8] and Ho et al. [7] studies and was predicted as endothelial here but it was not ranked first, ROBO4 and MMP1 ranked higher. Real time PCR (Figure 3) and *in-situ *(unpublished data) show extreme endothelial specificity for ECSM2 and its lower ranking is simply due to fewer ESTs, i.e. it is expressed at a lower level in the cDNA libraries. A comparison with the endothelial genes found in this study with that of Ho et al. [7] reveals 30 of the 49 genes were predicted as significantly (q-value <= 0.01) up regulated in endothelial cells. A further 5 genes were endothelial specific but not to significance (q-value > 0.01). 14 genes failed to show significant or specific expression in endothelial cells. Interestingly, the second ranked endothelial gene from the [7] analysis, SHE, showed only a single endothelial EST in this analysis. We conclude that although tissue specific genes can be predicted by cDNA analysis alone, it is advisable to use as many data sources as possible in order to derive a comprehensive list of genes. Finally, our results show that it is better to use normal cell isolates than carcinoma cell lines or libraries derived from micro-dissected or FACS sorted cells for this type of analysis, since several 4characterised endothelial genes hit ESTs in these non-endothelial libraries (Table 3: VWF, ROBO4 and CDH5).

### Extended analysis to predict tumour endothelial markers

We extended the analysis to identify which of the endothelial genes were expressed in tumours but not normal tissue. This was achieved by combining the endothelial screen with an analysis that compared gene expression between tumour and normal bulk tissue libraries from several organs. A gene was a predicted TEM if it was preferentially expressed in endothelial cells and tumour tissues but absent in normal tissues. A list of 27 promising new TEMs based on these analyses is given in Additional file 29. Each cell in Additional file 29 represents the result for a tumour/foetal screen for a particular organ. Ultimately, we wanted to find genes that showed tumour and foetal specific expression in all or most of the organs at a statistically significant level. Cells with green coloured text represent this type of result, having 0 ESTs in the normal pool and a q-value of less than 0.01. Genes showing significant and specific expression in multiple organs (brain, skin, kidney and foetal lung) were PLOD3 and THRAP4. However, these genes showed expression in normal tissue for several other organs. Likewise some genes were significantly up regulated in tumours in multiple organs but were not specific to tumours (cells in blue). These genes, although putative TEMs, were considered of least therapeutic value, as some expression was evident in normal tissues and as such they can not be used to specifically target tumours.

### Investigation of a subset of predicted TEMs

As endothelium comprises less than 5% of tumour tissue, it was hypothesised that genes with a tumour specific although not statistically significantly different expression could still be a TEM. Such genes are shown in Additional file 29 in red text. The most promising TEMs from Additional file 29 were selected based on little or no expression in normal tissue across all or multiple organs (red and green cells). Of these, angiopoietin 2 (ANGPT2), protocadherin 12 (PCDH12) and leucine rich repeat containing 8 family member C (LRRC8C) had expression profiles completely restricted to tumour or foetal tissues. ANGPT2, in these in silico results, was restricted to renal and colon tumour tissue in adults and lung in the embryo. This result is supported by the literature that indicates ANGPT2 is associated with tumour endothelium and tumour progression [34-37]. In contrast, leucine rich repeat containing 8 family member C was not yet reported to be a TEM in the literature but rather a gene responsible for adipocyte differentiation [38]. The 9 putative novel TEMs with the best tumour profile are listed in Table 6. The table excludes genes that already have a substantial literature (e.g. angiopoietin2). A final gene worthy of note is mediator of RNA polymerase II transcription subunit 28 homolog (*S. cerevisiae*, MED28): Previous work has shown MED28 to be significantly up-regulated in tumours, its over expression is able to stimulate cellular proliferation and its expression is up-regulated by endothelial cells when exposed to tumour media [39,40].

**Table 6:** The top Nine Tumour Endothelial Markers

Gene	Product
SPHK1	sphingosine kinase 1 isoform 2
KCTD15	potassium channel tetramerisation domain containing 15
LRRC8C	factor for adipocyte differentiation 158
PCDH12	protocadherin 12 precursor
C12orf11	hypothetical protein LOC55726
ECSM2	hypothetical protein LOC641700
GBP4	guanylate binding protein 4
IKBKE	IKK-related kinase epsilon
MED28	mediator of RNA polymerase II transcription, subunit 28 homolog

This table lists the top nine tumour endothelial markers from the analyses.

### TEM experimental validation

*In situ *hybridisation and immunostaining are the most definitive direct methods to experimentally validate the predicted TEMs in Table 6. However, the sensitivity to optimisation of in situ hybridisation makes it problematic for high throughput analysis. The second requires antibodies and the time to prepare these slows progress. Therefore to validate our approach at this time, the next section describes the analyses and literature search results for previously predicted tumour endothelial markers.

### An assessment of the TEM prediction fidelity based on previously validated TEMs

#### Delta4

Delta4 has been cited to have endothelial specific expression [41-43] and to be up regulated in tumour vessels [41,44]. In this study, Delta4 (DLL4) was endothelial specific but was expressed at a very low level in endothelial cell cultures. Thus, delta4 matched one EST from the endothelial pool and none from the non-endothelial pool, with an FDR-adjusted q-value of 0.28. Even though delta4 was not statistically significantly up regulated in endothelial cells, it showed some evidence of being endothelial specific as there were no ESTs found from the non-endothelial pool. DLL4 was found in brain and colon tumour tissues. However, the expression was not specific or significant in tumours. Thus, in our analysis DLL4 was not a predicted TEM.

#### GPR124

GPR124 (TEM5) was previously identified as a putative TEM using custom SAGE library analysis [14]. In the current analysis, GPR124 failed to match any endothelial ESTs from the 31,114 EST in the endothelial pool. From the non-endothelial pool, GPR124 did match a single EST [GenBank:BF325872] from the AN0041 cDNA library derived from a normal amniotic fluid cell line. These results suggest that GPR124 is only expressed at a low level in normal tissue and is absent or at a very low level in cultured endothelial cells. In contrast, GPR124 was predicted as significantly and specifically up regulated in multiple tumour tissues. Thus, GPR124 appears to be a tumour but not a tumour endothelial marker.

#### TEM1

TEM1 (endosialin or CD 248) [14] has a count of 1 and 2 ESTs for the endothelial and non-endothelial pools respectively. The FDR-adjusted q-value for this gene was 0.61, a non-significant value. One EST from the non-endothelial pool, accession [GenBank:CN484271] was from a primary human ocular pericyte cDNA library. This agrees with experimental findings of MacFadyen et al. [45,46] that have shown that endosialin is expressed by fibroblasts and a subset of pericytes associated with tumour vessels but not by tumour endothelium.

#### SPARC

Numerous studies have reported SPARC to be up regulated in endothelial cells, to have a role in tissue remodelling and be linked to tumour progression [47-50]. Our analysis strongly predicted SPARC to be a TEM. SPARC was up regulated in endothelial cells with a significant q-value of 8.4 × 10^-10 ^and also significantly up regulated in brain, colon, kidney and prostate tumour tissue.

#### ROBO4

Several groups have independently reported ROBO4 as a TEM [51-53]. In this study ROBO4 was highly endothelial specific, both from the in silico and experimental analyses. In the tumour screen, ROBO4 was seen to be tumour specific in brain and kidney tumour tissues but not at a statistically significant level. Thus ROBO4 was predicted as a tumour endothelial marker, but not in all tumour types. In this case our analysis may be under predictive, as experimentally ROBO4 has been found to be a strong TEM [52,53].

These results demonstrate the absolute need for experimental verification of bioinformatics predictions and there is evidence both for and against the use of cDNA analyses for the prediction of TEMs. If TEM1, TEM5 and DLL4 are true TEMs then this technique is not 100% reliable. Conversely, the successful prediction of ROBO4, ANGPT2, VIM and SPARC shows that these methods do have the ability to predict a validated TEM. The novel predictions of this analysis await future validation.

### Differential gene expression analyses using these methods through a web tool

A web tool has been developed to enable researchers to take advantage of these new algorithms and design their own differential gene expression analysis [54]. As far as possible, each Human EST from all of the cDNA libraries at Genbank has been assigned to a Refseq gene or gene prediction based on the new Jake cluster algorithm so that any tissue of interest can be queried provided cDNA libraries exist. The interface lets the user select two pools of cDNA libraries, group A and group B, to be compared and produces a list of differentially expressed genes. The results are returned to the user as a table, reporting the q-value and whether the gene is up or down regulated in pool A. It is hoped these new algorithms and statistical methods will enable the rapid prediction of differentially expressed genes.

## Conclusion

New cDNA library data is continually been submitted to Genbank and the amount of relevant information that can be mined is increasing. cDNA library analysis has been improved in this work by more accurate EST to gene assignment and the best possible statistics applied to the data. Using these tools on the latest data sets will lead to the prediction of new biologically and therapeutically important genes. This is enhanced by the statistics as unlike DDD at NCBI, they permit the inclusion of cDNA libraries of all sizes.

We have shown that these methods accurately predict the identity of endothelial and tumour endothelial genes by comparing our results with that of known genes. ROBO4 has consistently been shown to be highly endothelial specific and was ranked second in this work. TEMs are also successfully predicted as shown by SPARC and Angiopoeitin 2.

It is hoped that biologists will take advantage of these methods for their disease of interest by using the online tool. An interface has been designed such that a user can select tissue, histology, preparation and protocol of interest.

## Methods

### Construction of databases

A large part of this study involved the collection and processing of data in the public domain with speed and accuracy, in particular the creation and use of a Relational Database Management System (RDBMS) MySQL database called dbestlibraries. The database was central to all processes in tandem with Perl scripts (see Additional files 30, 31, 32, 33, 34, 35, 36), which were written for the import of data, assignment of EST to gene symbols and the accurate calculation of the FDR-adjusted q-value results. The development of the database involved creating tables that housed EST information such as library, tissue and accession numbers. This enabled the fast retrieval of EST accessions for each of the libraries when an analysis was performed. Other tables stored used were gene, Refseq, Unigene and the results of each SAGE and cDNA library analysis. Apart from advantages in archiving results, it also enabled the cross referencing of SAGE and cDNA results and the assignment of gene symbols etc. See Additional file 37 for an overview of the table structure of dbestrlibraries.

Data was collected from Genbank flat files (release 154) downloaded from the NCBI [55] that supplied all cDNA library data imported into the database. 10,788 libraries containing 8,003,786 ESTs were imported into the database. Information concerning 29,367 human reference sequence project mRNAs and gene predictions were downloaded from release 14 of the Reference sequence project [56]. Finally, all information relating to Refseq sequences was downloaded and imported into the database using data downloaded [57].

### Selection of EST library pools

The CGAP library finder [58] was used as a tool for choosing which libraries to compare in tumour and endothelial screens. Additional endothelial cDNA libraries were discovered, using a Perl script to parse raw Genbank flat files, which identified libraries with keywords such as "cell lines" and "endothelial". Normalised or subtracted libraries were excluded from this analysis.

### Normal versus tumour tissue screen

Bulk tumour and normal cDNA libraries for 6 organs were chosen using the CGAP library browser. The combined algorithm was employed to perform virtual subtraction hybridisation between tumour and normal libraries of the same organ. All results were imported into the dbestlibraries database. Results with an FDR-adjusted q-value of <= 0.01 were significant.

### cDNA library screen (EST to gene assignment)

To perform in silico virtual subtraction, two different protocols for assigning an EST to a gene were combined for greatest accuracy. The first protocol took advantage of the almost complete human genome by using genome address to assign an EST to a gene. A genome address of a gene or EST is the physical base pair position it occupies on a chromosome. Both cDNA pools and all Refseq mRNAs were aligned to the human genome using BLAT to generate genome addresses. The BLAT alignment genome addresses were clustered using a Perl algorithm called the Jake cluster algorithm to identify EST sequences that overlapped with a gene and to assign them. To save processing time using BLAT, the human genome addresses of Refseq genes and ESTs were downloaded from the University of California Santa Cruz (UCSC) table browser page [59]. This file contained the pre-processed BLAT output [22]. BLAT is designed to rapidly align DNA sequences that are 95% identical or more, over at least 40 base pairs.

For the second method of assigning an EST to a gene, each EST from both cDNA library pools was collected as a FASTA sequence and BLAST searched against a database of all Refseq mRNAs. A liberal e-value [23] cut-off of 1 was employed and the -v and -b BLAST options were set to 1. This ensured that only the best mRNA that matched the EST was returned in the BLAST results.

A Perl script algorithm (Jake cluster, Additional file 32) was utilised to combine the results of the genome BLAT address with the BLAST search method. If the genome address assignment agreed with the BLAST result, then the EST was assigned to the gene, if they disagreed, only a high quality BLAST result allowed EST to gene assignment (>= 92% identity, >= 100 bp alignment).

### Combining cDNA and SAGE library analysis

For experiments 2 and 3 described in the results section, the cDNA analysis was combined with a SAGE library analysis for endothelial gene prediction. The SAGEmap xProfiler tool at NCBI was used for this: Available from the SAGEmap website [20]. No SAGE analyses were carried out for the tumour screen, as there were insufficient bulk tumour or bulk normal libraries SAGE libraries available.

For experiments 2 and 3 the SAGE xProfiler analyses were performed using a fold difference factor of 10 and a 0% coefficient of variance cut off. Only genes with a posterior probability of 0.9 or more were considered significant. In pool "A" (endothelial cell line pool) there were 10 SAGE libraries containing 427,254 SAGE transcripts. In pool "B", the normal non-endothelial pool, there were 11 normal non-endothelial libraries with 329,470 transcripts. For the cancer cell line non-endothelial pool [8], there were 24 SAGE libraries consisting of 733,461 transcripts. As the cancer cell line non-endothelial pool was twice the size of the normal non-endothelial pool, more genes were significantly up regulated in the former due to pool size and statistics.

### Statistical methods

We now describe a statistical methodology for the comparison of two groups of cDNA libraries to enable the discovery of differentially expressed genes. The method combines a likelihood ratio test with a False Discovery Rate procedure (FDR) in order to provide a robust list of differentially expressed genes. The analyses extend our earlier work, which identified differentially expressed genes in a single group of cDNA libraries [15].

As described in [15], we consider the expression of gene *j *in a set of cDNA libraries. There are two groups of libraries: *m *libraries from non-endothelial cell lines, and *n *libraries from endothelial cell lines. We let *N*_*i *_: 1 ≤ *i *≤ *m *be the number of ESTs sequenced in each non-endothelial cell line library, and *N*_*m*+*i *_: 1 ≤ *i *≤ *n *be the number of ESTs sequenced in each of the endothelial cell line library. For each gene *j*, let *x*_*i*,*j *_be the number of copies of associated ESTs in library *i*.

For each gene, we compare two hypotheses concerning its frequency of expression in the libraries, using a likelihood ratio test. Under the null hypothesis, the gene is not differentially expressed and we would expect its frequency to be identical in both the non-endothelial and endothelial cell libraries. In contrast, under the alternative hypothesis, the gene is differentially expressed, and so we would expect the frequency to be different in the non-endothelial and endothelial cell lines.

In both cases, as long as the number of copies of ESTs from the gene is small relative to the total number of ESTs sequenced in the library, the distribution of the gene is well approximated by a Poisson distribution. Under the null hypothesis, the frequency is *f*_*j*_, then for library *i*, the number of ESTs is approximately distributed as a Poisson variable with parameter *f*_*i*_*N*_*i*_. Thus the likelihood function is

$$L^{0}=\prod_{i=1}^{m+n}\frac{e^{-f_{j}N_{i}}{\left(f_{j}N_{i}\right)}^{x_{i,j}}}{X_{i,j}!}$$

The likelihood estimate of *f*_*j *_under the null hypothesis can be found by solving:

$$\frac{dL^{0}}{df_{j}}=0$$

And the solution *f*_*j*_^(0) ^is given by:

$$f_{j}^{\left(0\right)}=\frac{\sum_{i=1}^{m+n}x_{i,j}}{\sum_{i=1}^{m+n}N_{i}}$$

Equation 3 is simply the proportion of ESTs for the gene of interest among all ESTs in all of the libraries. Thus under the null hypothesis, the likelihood function (*L*^0 ^_*j*_) is given by equation 1 with *f *= *f*_*j*_.

For the alternative hypothesis, the frequency of gene transcripts is different in the non-endothelial and endothelial cell line libraries. By a similar argument, we derive frequencies for each gene *j *in the non-endothelial libraries *f*_*j*_^(1) ^and the endothelial libraries *f*_*j*_^(2) ^which is given by:

$$f_{j}^{\left(1\right)}=\frac{\sum_{i=1}^{m}x_{i,j}}{\sum_{i=1}^{m}N_{i}}$$

$$f_{j}^{\left(2\right)}=\frac{\sum_{i=1}^{n}x_{m+i,j}}{\sum_{i=1}^{n}N_{m+i}}$$

Observe that equations 4 and 5 are very similar to equation 3, and simply represent the proportion of ESTs for the gene of interest among all ESTs in the relevant libraries. Under the alternative hypothesis, the likelihood function is given by:

$$L_{j}^{1}=\prod_{i=1}^{m}\frac{e^{-f_{j}^{\left(1\right)}N_{i}}{\left(f_{j}^{\left(1\right)}N_{i}\right)}^{x_{i,j}}}{x_{i,j}!}\prod_{i=1}^{n}\frac{e^{-f_{j}^{\left(2\right)}N_{m+i}}{\left(f_{j}^{\left(2\right)}N_{m+i}\right)}^{x_{m+i,j}}}{x_{m+i,j}!}$$

And thus the log likelihood ratio is:

$$R_{j}=\log(f_{j}^{\left(1\right)}/f_{j}^{\left(0\right)})\sum_{i=1}^{m}x_{i,j}+\log(f_{j}^{\left(2\right)}/f_{j}^{\left(0\right)})\sum_{i=1}^{n}x_{m+i,j}$$

Equation 7 can be explained very simply: there are two terms, one for the non-endothelial libraries and one for the endothelial libraries. Each term is the log ratio of the frequency of the gene in the relevant libraries and the overall frequency of the gene, multiplied by the total number of ESTs for that gene in the relevant libraries. The equation is very similar to the R statistic derived in Stekel et al. 2000 [15].

Under Wilke's Theorem [60], 2*R*_*j *_is asymptotically distributed as a *X*^2 ^distribution with a 1 degree of freedom. Alternatively, a randomization procedure can be used to derive a p-value not dependent on a *X*^2 ^approximation as described in Stekel et al. 2000 [15]. Random data for the total EST count in the normal and disease libraries are generating using Poisson distributions with parameters based on the null hypothesis frequency are generated 1,000,000 times and the R statistic is computed for each data set. This gives an empirical distribution for the R statistic against which the value of R from the real data is compared to generate a p-value. Where several genes shared the same EST counts, only one distribution was used, so that these genes would have the same p-values. However, when analyzing all genes in the library in order to find those that are most differentially expressed, it is essential to combine the p-value with a False Discovery Rate Procedure [25]. Thus the results we present are the FDR-adjusted q-values [26].

### A definition of terms

*m *= the number of non-endothelial cell line libraries

*n *= the number of endothelial cell line libraries

*x*_*i*,*j *_= number of transcript copies of gene *j *in cDNA library *i*

*N*_*i *_= the total number of clones sequenced in the cDNA library *i*

*x*_*m*+*i*,*j *_= the number of copies of gene *j *in the *m *+ *i*'th cDNA library

*N*_*m*+*i *_= is the total number of clones sequenced in the *m *+ *i*'th cDNA library

*f*_*j *_= is the frequency of gene *j*

### Computation of the statistics

To compute the FDR adjusted q-values [26] for a given data set, we calculate the R-values for all genes. We then computed the p-values for every gene using both the Chi Squared value of 2R and the randomization method. The genes were then ordered according to the p-values, ranked from smallest to highest. Each p-value was adjusted by multiplying it by the number of genes in the analysis and dividing by its rank position (The smallest p-value is rank position 1). To derive the q-value [26], the list of ranked values was stepped through, comparing p-value and its adjusted value and always selecting the lowest.

### Calculation of cDNA library Posterior probabilities for statistical comparison (experiment 1)

Source code of the SAGEmap xProfiler tool was downloaded from the NCBI [61]. All the defaults for the program were used except for the c value in statistic which was set to 3 based on the findings of [62]. The target fold difference factor was left at 2.

### Calculation of cDNA library Susko and Roger statistics

Software and documentation was downloaded from the Susko and Roger 2004 website [19]. The software was installed and a Perl wrapper script (Additional file 36) was used to execute the expr_est differential gene expression software. In preparation of running this software, all the non-endothelial and endothelial libraries were combined into two groups and differential gene expression was measured between these two groups using the expr_est software via the Perl wrapper.

### Cell isolates and extraction of RNA

Human umbilical vein endothelial cells (pooled HUVEC), adult human dermal microvascular endothelial cells (HDMEC), human bronchial epithelial cells (HBEC) and adult human Epidermal keratinocytes were obtained from TCS Cellworks (Botolph Claydon, UK). Cells were grown in their appropriate growth media and supplements, according to manufacturers' instructions and RNA extracted at passage 2–3. Human lung fibroblasts (MRC-5) were obtained from the American Type Culture Collection (Manassas, VA) and cultured in DMEM containing 10% FCS. All cells were grown at 37°C in a humidified atmosphere of 5% CO2 in air.

Cryopreserved human hepatocytes (TCS Cellworks) were thawed in Leibovitch L15 medium (Invitrogen, Paisley, UK), centrifuged and resuspended in fresh media, RNA was extracted after 30 minutes incubation at 37°C in 5% CO2. Cryopreserved human peripheral blood lymphocytes were obtained from TCS Cellworks, after thawing they were washed in PBS and used immediately for RNA extraction.

### Quantitative PCR

Total RNA was extracted from cells in culture using TRI reagent (Sigma, Dorset, UK) cDNA was prepared using a high capacity cDNA archive kit (Applied Biosystems, Cheshire, UK). The Universal ProbeLibrary system (Roche) was used for real time PCR analysis (see Additional file 38 for primer sequences and Universal probe numbers). Reactions were performed in triplicate using Absolute qPCR mix (ABgene, Epsom, UK) according to manufactures instructions using 10 ng of cDNA.

Reactions were performed in a Rotor-GENE RG30000 thermocycler (Corbett Reaearch, UK) using the following cycling conditions; 95°C for 10 minutes followed by 40 cycles of 95°C for 15 seconds and 60°C for 1 minute. The appropriate housekeeper genes were determined as described by Vandesompele et al [63] using the software geNorm. For the cell type screen FLOT2, Ubiquitin C and B-Actin were used. The raw data was analysed using a method described by Pfaffl [64].

## Abbreviations

BLAST, Basic Local Alignment Search Tool; BLAT, BLAST Like Alignment Tool; cDNA, complementary DNA; DDD, Digital Differential Display; EST, Expressed Sequence Tag; FDR, False Discovery Rate; FDR-adjusted, False Discovery Rate adjusted; FACS, Fluorescence-activated cell sorting; GFP, Green Fluorescent Protein; HBEC, human bronchial epithelial cells; HDMECs, human dermal micro-vascular endothelial cells; HUVECs, human umbilical vein endothelial cells; MRC-5, Human lung fibroblasts; PCR, Polymerase Chain Reaction; RDBMS, Relational Database Management System; Refseq, Reference Sequence Project; SAGE, Serial Analysis of Gene Expression; TEM, Tumour Endothelial Marker;

## Authors' contributions

JH performed the bioinformatic analyses, conceived the new method of EST to gene assignment and wrote the paper. DS conceived the statistical analysis. SS performed the Real Time PCR analyses. VH helped in the interpretation of the data and the writing of the paper. RB conceived the overall project, helped with the interpretation of the data and the writing of the paper. All authors read and approved the final manuscript.

## Supplementary Material

Additional File 1 174 endothelial genes were found applying the new cDNA library analysis and statistics to the cDNA library data used by Huminiecki and Bicknell (2000) [8]. 14 of these genes showed a statistically significant endothelial specific expression profile. Click here for file

Additional file 2 There were 136,336 ESTs from 208 Genbank normal, non-endothelial cDNA libraries that were used in experiments one and two. Click here for file

Additional file 3 Comparisons of the most significantly differentially expressed genes using different statistical methods. Click here for file

Additional file 4 From the latest available endothelial cDNA library data, 424 genes were predicted to be statistically significantly up regulated in endothelial cells using a posterior probability of >= 0.9 with a target fold difference factor of 2. The table also shows Susko and Roger statistics, results of the Poisson statistics with p-values generated from Chi Square and randomization analyses for comparison. Click here for file

Additional file 5 554 genes were found to be significantly up or down regulated in endothelial cells based on a q-value <= 0.01 generated using a Chi Square p-value. randomization q-values (Stekel et al. 2000), Susko and Roger statistics and posterior probabilities are also displayed for comparison. Click here for file

Additional file 6 661 genes were found to be significantly up or down regulated in endothelial cells based on a q-value <= 0.01 generated using a randomization procedure (Stekel et al. 2000). Chi Square q-values, Susko and Roger statistics and posterior probabilities are also displayed for comparison. Click here for file

Additional file 7 536 genes were found to be significantly up or down regulated in endothelial cells based on the Susko and Roger statistics. Chi Square q-values, randomization procedure (Stekel et al. 2000) q-values and posterior probabilities are also displayed for comparison. Click here for file

Additional file 8 From the latest available endothelial cDNA library data, 431 genes predicted to be statistically significantly up regulated in endothelial cells. 104 genes showed an endothelial specific profile. Click here for file

Additional file 9 SAGE libraries used for experiment 2 were collected from SAGEmap. There were 10 endothelial libraries consisting of 427,254 tags and 11 normal non-endothelial libraries 329,470 tags. Click here for file

Additional file 10 27 genes were predicted to be endothelial specific using a combined SAGE and cDNA library analysis of the latest libraries. The genes are sorted in descending order according to the number of non-endothelial library hits. Click here for file

Additional file 11 Experiment 3 included cDNA and SAGE libraries from cancer, microdissected and flow sorted cell lines. 178,653 ESTs and 733,461 SAGE tags were contained within these library pools. Click here for file

Additional file 12 58 endothelial specific genes were predicted by SAGE-CGAP xProfiler. All SAGE and cDNA non-endothelial cell libraries, including those from transformed cell lines and those produced by tissue micro-dissection or cell sorting, were used in this analysis. Click here for file

Additional file 13 By combining all the genes found from experiments 1, 2 and 3, a non-redundant comprehensive list of 459 endothelial genes is produced. HUGO and Refseq interim gene symbols are presented. Click here for file

Additional file 14 237 Brain tumour bulk tissue libraries containing 140,621 ESTs were used versus brain normal libraries to find differentially expressed genes. Click here for file

Additional file 15 24 Brain foetal bulk tissue libraries containing 69,862 ESTs were used versus brain normal libraries to find differentially expressed genes. Click here for file

Additional file 16 302 Brain normal bulk tissue libraries containing 100,554 ESTs were used versus brain tumour/foetal libraries to find differentially expressed genes. Click here for file

Additional file 17 178 lung tumour bulk tissue libraries containing 108,107 ESTs were used versus lung normal libraries to find differentially expressed genes. Click here for file

Additional file 18 10 lung foetal bulk tissue libraries containing 112,690 ESTs were used versus lung normal libraries to find differentially expressed genes. Click here for file

Additional file 19 91 lung normal bulk tissue libraries containing 82,757 ESTs were used versus lung tumour/foetal libraries to find differentially expressed genes. Click here for file

Additional file 20 7 kidney bulk tumour tissue libraries containing 38,519 ESTs were used versus kidney normal libraries to find differentially expressed genes. Click here for file

Additional file 21 5 kidney bulk foetal tissue libraries containing 2,605 ESTs were used versus kidney normal libraries to find differentially expressed genes. Click here for file

Additional file 22 5 kidney bulk normal tissue libraries containing 72,476 ESTs were used versus kidney tumour/foetal libraries to find differentially expressed genes. Click here for file

Additional file 23 131 prostate bulk tumour tissue libraries containing 19,125 ESTs were used versus prostate normal libraries to find differentially expressed genes. Click here for file

Additional file 24 129 prostate bulk normal tissue libraries containing 68,480 ESTs were used versus prostate tumour libraries to find differentially expressed genes. Click here for file

Additional file 25 6 skin bulk tumour tissue libraries containing 12,484 ESTs were used versus skin normal libraries to find differentially expressed genes. Click here for file

Additional file 26 4 skin bulk normal tissue libraries containing 33,218 ESTs were used versus skin tumour libraries to find differentially expressed genes. Click here for file

Additional file 27 557 colon bulk tumour tissue libraries containing 143,025 ESTs were used versus colon normal libraries to find differentially expressed genes. Click here for file

Additional file 28 134 colon bulk normal tissue libraries containing 37,269 ESTs were used versus colon tumour libraries to find differentially expressed genes. Click here for file

Additional file 29 Potential Tumour Endothelial Markers and their likelihood ratio test statistic results. Click here for file

Additional file 30 A quick guide to what is needed for setting up the analysis pipeline locally. Click here for file

Additional file 31 This program takes in tab delimited format of Genes, ESTS or Unigene clusters BLAT mapped onto the Human Genome (UCSC genome browser, table browser) and stores a hash on disk in the Perl data format needed for clustering overlapping sequences based on genome Chromosome position. Click here for file

Additional file 32 Calls the clustering algorithm from the tem_scripts module that clusters Genes to ESTS based on Human Genome Mapping position. Click here for file

Additional file 33 A Perl module that houses many Perl routines for assigning EST to genes, database manipulation and gene annotation. Click here for file

Additional file 34 This Perl program performs the likelihood ratio test using p-values generated on the basis of 2R been Chi Squared distributed. It also produces FDR q-values. Click here for file

Additional file 35 This is a bash shell script that enables the simultaneous execution of the Perl programs for the pipeline analysis. Click here for file

Additional file 36 This program is a wrapper to the Susko and Roger expr_est program which measures differential expression between two cDNA libraries. Click here for file

Additional file 37 A schema of the database constructed to store cDNA library data, gene/Refseq annotations and cDNA/SAGE library analysis results. Click here for file

Additional file 38 Real Time PCR was carried out to find preferential expression of genes amongst several cell isolates. This lists the primers used for each of the genes. Click here for file
