# Supplementary material for: A novel method of differential gene expression analysis using multiple cDNA libraries applied to the identification of tumour endothelial genes
Source: BMC Genomics. 2008 Apr 7;9:153. doi: 10.1186/1471-2164-9-153 (PMC2346479; doi:10.1186/1471-2164-9-153)
Supplement: Additional File 1 — 174 endothelial genes were found applying the new cDNA library analysis and statistics to the cDNA library data used by Huminiecki and Bicknell (2000) [8]. 14 of these genes showed a statistically significant endothelial specific expression profile. [file 1471-2164-9-153-S1.doc]

**Additional File 1:** 174 endothelial genes were found applying the new cDNA library analysis and statistics to the cDNA library data used by Huminiecki and Bicknell (2000). 14 of these genes showed a statistically significant endothelial specific expression profile.

| **Gene** | **q-value** | **Endo Count** | **Non-Endo Count** |
| --- | --- | --- | --- |
| *ECSM2* | 0.0000 | 9 | 0 |
| *TFPI* | 0.0000 | 7 | 0 |
| *MMRN1* | 0.0000 | 5 | 0 |
| *TIE1* | 0.0000 | 5 | 0 |
| *ACTA1* | 0.0000 | 5 | 0 |
| *ECSM1* | 0.0002 | 4 | 0 |
| *CD34* | 0.0002 | 4 | 0 |
| *BMX* | 0.0031 | 3 | 0 |
| *LOC650049* | 0.0031 | 3 | 0 |
| *APLN* | 0.0031 | 3 | 0 |
| *DUS4L* | 0.0031 | 3 | 0 |
| *FABP4* | 0.0031 | 3 | 0 |
| *LOC643977* | 0.0031 | 3 | 0 |
| *PAQR3* | 0.0031 | 3 | 0 |
| *ANXA2* | 0.0000 | 174 | 193 |
| *SERPINE1* | 0.0000 | 83 | 94 |
| *MMP1* | 0.0000 | 54 | 18 |
| *TMSB4X* | 0.0000 | 92 | 162 |
| *VIM* | 0.0000 | 66 | 72 |
| *EFEMP1* | 0.0000 | 40 | 8 |
| *PPIA* | 0.0000 | 88 | 196 |
| *S100A6* | 0.0000 | 58 | 62 |
| *ACTG1* | 0.0000 | 110 | 344 |
| *RPL19* | 0.0000 | 58 | 85 |
| *VWF* | 0.0000 | 27 | 1 |
| *RPL13A* | 0.0000 | 84 | 242 |
| *RPSA* | 0.0000 | 74 | 184 |
| *RPLP0* | 0.0000 | 104 | 404 |
| *TPT1* | 0.0000 | 68 | 170 |
| *RPL3* | 0.0000 | 67 | 176 |
| *RPS8* | 0.0000 | 50 | 90 |
| *RPS27A* | 0.0000 | 60 | 142 |
| *RPL9* | 0.0000 | 56 | 122 |
| *PECAM1* | 0.0000 | 24 | 5 |
| *RPS15* | 0.0000 | 33 | 28 |
| *TMSB10* | 0.0000 | 42 | 63 |
| *RPS18* | 0.0000 | 66 | 195 |
| *FTH1* | 0.0000 | 81 | 305 |
| *RPS4X* | 0.0000 | 51 | 123 |
| *RPS3A* | 0.0000 | 60 | 179 |
| *RPS24* | 0.0000 | 52 | 131 |
| *C4orf18* | 0.0000 | 17 | 1 |
| *RPL37A* | 0.0000 | 59 | 184 |
| *EEF1A1* | 0.0000 | 137 | 837 |
| *SPARC* | 0.0000 | 46 | 108 |
| *RPL17* | 0.0000 | 38 | 68 |
| *RPS2* | 0.0000 | 63 | 218 |
| *RPS20* | 0.0000 | 57 | 179 |
| *RPL31* | 0.0000 | 51 | 148 |
| *RPS12* | 0.0000 | 41 | 90 |
| *RPL6* | 0.0000 | 45 | 117 |
| *LGALS1* | 0.0000 | 37 | 74 |
| *RPL7A* | 0.0000 | 45 | 123 |
| *RPS3* | 0.0000 | 61 | 239 |
| *CTGF* | 0.0000 | 30 | 49 |
| *RPL26* | 0.0000 | 39 | 96 |
| *RPS15A* | 0.0000 | 34 | 73 |
| *MCAM* | 0.0000 | 25 | 32 |
| *RPL5* | 0.0000 | 34 | 76 |
| *TUBA3* | 0.0000 | 22 | 24 |
| *RPS10* | 0.0000 | 32 | 69 |
| *TXNDC5* | 0.0000 | 25 | 42 |
| *RPL4* | 0.0000 | 39 | 121 |
| *RPLP1* | 0.0000 | 37 | 109 |
| *RPL23* | 0.0000 | 33 | 86 |
| *RPS13* | 0.0000 | 37 | 111 |
| *RPL24* | 0.0000 | 32 | 81 |
| *RPL27A* | 0.0000 | 36 | 109 |
| *RPS26* | 0.0000 | 21 | 30 |
| *RPS6* | 0.0000 | 42 | 153 |
| *IFI27* | 0.0000 | 13 | 5 |
| *RPS27* | 0.0000 | 28 | 65 |
| *ITGA5* | 0.0000 | 15 | 11 |
| *RPL10A* | 0.0000 | 32 | 93 |
| *RPS7* | 0.0000 | 34 | 106 |
| *RPL7* | 0.0000 | 27 | 67 |
| *RPS19* | 0.0000 | 36 | 125 |
| *RPL32* | 0.0000 | 36 | 127 |
| *WBP2* | 0.0000 | 15 | 14 |
| *NACA* | 0.0000 | 24 | 57 |
| *LDHB* | 0.0000 | 24 | 62 |
| *MGP* | 0.0000 | 13 | 12 |
| *SLC25A3* | 0.0000 | 19 | 37 |
| *RPL23A* | 0.0000 | 32 | 126 |
| *IFITM3* | 0.0000 | 18 | 37 |
| *MARCKSL1* | 0.0000 | 17 | 32 |
| *RPS25* | 0.0000 | 28 | 101 |
| *RPLP2* | 0.0000 | 23 | 68 |
| *GNB2L1* | 0.0000 | 51 | 300 |
| *CALM2* | 0.0000 | 19 | 47 |
| *MT2A* | 0.0000 | 16 | 32 |
| *ACTB* | 0.0000 | 59 | 388 |
| *RPL14* | 0.0000 | 19 | 50 |
| *PROCR* | 0.0000 | 9 | 6 |
| *EDN1* | 0.0000 | 7 | 2 |
| *RPL11* | 0.0000 | 24 | 89 |
| *IL32* | 0.0000 | 11 | 14 |
| *RPS14* | 0.0000 | 31 | 146 |
| *NPM1* | 0.0000 | 27 | 114 |
| *RPL18* | 0.0000 | 22 | 81 |
| *RPS5* | 0.0000 | 25 | 105 |
| *ATP5C1* | 0.0000 | 11 | 18 |
| *RPL37* | 0.0000 | 22 | 86 |
| *ITGB1* | 0.0000 | 20 | 72 |
| *PRCP* | 0.0000 | 10 | 14 |
| *LOC653949* | 0.0001 | 13 | 29 |
| *HHIP* | 0.0001 | 6 | 2 |
| *SDPR* | 0.0001 | 6 | 2 |
| *RPS11* | 0.0001 | 30 | 158 |
| *FTL* | 0.0001 | 28 | 141 |
| *PFDN5* | 0.0001 | 12 | 26 |
| *LOC653713* | 0.0001 | 17 | 57 |
| *C6orf130* | 0.0002 | 5 | 1 |
| *SLC7A7* | 0.0002 | 5 | 1 |
| *POLR2L* | 0.0002 | 8 | 9 |
| *SEC61B* | 0.0002 | 6 | 3 |
| *EEF1G* | 0.0002 | 38 | 240 |
| *SMAD1* | 0.0002 | 7 | 6 |
| *FKBP1A* | 0.0002 | 17 | 60 |
| *MYL6* | 0.0003 | 25 | 123 |
| *ATP5O* | 0.0004 | 12 | 30 |
| *RPL36* | 0.0004 | 16 | 56 |
| *GSTO1* | 0.0005 | 14 | 43 |
| *UBA52* | 0.0005 | 22 | 103 |
| *RPS23* | 0.0005 | 13 | 37 |
| *RPL34* | 0.0005 | 18 | 72 |
| *PARVB* | 0.0007 | 5 | 2 |
| *RPL18A* | 0.0007 | 22 | 106 |
| *RPS16* | 0.0007 | 15 | 52 |
| *SRP14* | 0.0008 | 13 | 39 |
| *CD63* | 0.0008 | 11 | 27 |
| *RPL29* | 0.0008 | 18 | 75 |
| *ARPC3* | 0.0012 | 10 | 23 |
| *TM4SF1* | 0.0012 | 10 | 23 |
| *SHFM1* | 0.0012 | 8 | 13 |
| *RPL38* | 0.0018 | 13 | 43 |
| *RANBP1* | 0.0018 | 8 | 14 |
| *RPL15* | 0.0018 | 25 | 141 |
| *PRDX1* | 0.0022 | 19 | 90 |
| *FRMD4B* | 0.0022 | 4 | 1 |
| *CDH5* | 0.0022 | 4 | 1 |
| *ROBO4* | 0.0022 | 4 | 1 |
| *CD93* | 0.0022 | 4 | 1 |
| *C14orf78* | 0.0022 | 4 | 1 |
| *NDUFB9* | 0.0025 | 8 | 15 |
| *HN1* | 0.0027 | 10 | 26 |
| *FUNDC2* | 0.0032 | 6 | 7 |
| *LOC440990* | 0.0034 | 8 | 16 |
| *CD99* | 0.0035 | 12 | 40 |
| *K-ALPHA-1* | 0.0036 | 39 | 291 |
| *XR_001013* | 0.0039 | 5 | 4 |
| *FBL* | 0.0041 | 9 | 22 |
| *P4HB* | 0.0042 | 10 | 28 |
| *C10orf58* | 0.0045 | 7 | 12 |
| *LAMA4* | 0.0052 | 6 | 8 |
| *UBC* | 0.0054 | 17 | 81 |
| *TGM2* | 0.0064 | 8 | 18 |
| *ATP5H* | 0.0066 | 7 | 13 |
| *HNRPA1* | 0.0067 | 20 | 109 |
| *SSR2* | 0.0068 | 9 | 24 |
| *WDR70* | 0.0068 | 4 | 2 |
| *RHOBTB1* | 0.0068 | 4 | 2 |
| *RPS9* | 0.0073 | 19 | 101 |
| *ARMET* | 0.0074 | 5 | 5 |
| *PTRH2* | 0.0074 | 5 | 5 |
| *EIF1* | 0.0075 | 17 | 84 |
| *COX5B* | 0.0079 | 6 | 9 |
| *RPL10* | 0.0081 | 30 | 209 |
| *UBE2G2* | 0.0081 | 8 | 19 |
| *BTF3* | 0.0082 | 12 | 45 |
| *PTRF* | 0.0083 | 11 | 38 |
| *EDF1* | 0.0088 | 7 | 14 |
| *PLS3* | 0.0088 | 7 | 14 |
| *NPC2* | 0.0096 | 12 | 46 |
