# Supplementary material for: A novel method of differential gene expression analysis using multiple cDNA libraries applied to the identification of tumour endothelial genes
Source: BMC Genomics. 2008 Apr 7;9:153. doi: 10.1186/1471-2164-9-153 (PMC2346479; doi:10.1186/1471-2164-9-153)
Supplement: Additional file 2 — There were 136,336 ESTs from 208 Genbank normal, non-endothelial cDNA libraries that were used in experiments one and two. [file 1471-2164-9-153-S2.doc]

**Additional File 2:**  There were 136,336 ESTs from 208 Genbank normal, non-endothelial cDNA libraries that were used in experiments 1 and 2.

**Library EST counts**

Stratagene NT2 neuronal precursor 937230 5718

Stratagene neuroepithelium NT2RAMI 937234 2329

Stratagene hNT neuron (#937233) 7908

Stratagene fibroblast (#937212) 1640

Stratagene corneal stroma (#937222) 737

Stratagene cat#937212 (1992) 281

normal human keratinocytes 197

KG1-a Lambda Zap Express cDNA library 880

Human Trabecular Meshwork cDNA library 1021

Human salivary gland cell line HSG 11

Human promyelocyte 1461

Human primary human ocular pericytes.Unamplified (hw) 3538

Human primary human ocular pericytes. Equalized (hx) 906

Human epidermal keratinocyte 586

Hembase; Erythroid Progenitor Cells (LCB:ad library) 721

Hembase; Erythroid Precursor Cells (LCB:cl library) 9842

HeLa SRIG (Synthetic retinoids induced genes) 61

"CCRF-CEM cells, cyclohexamide treated I" 111

**Library EST counts**

NIH_MGC_56 15414

NIH_MGC_48 18761

NIH_MGC_43 11813

NIH_MGC_106 6521

NCI_CGAP_Pr25 1883

NCI_CGAP_Ov38 892

NCI_CGAP_HN6 571

Namalwa B cells I 244

LNCAP cells I 406

L17N670205n1 3784

ecnorm 625

L17N670205 593

BN0412 250

BN0411 156

BN0410 463

BN0409 48

BN0408 195

BN0407 113

BN0406 177

BN0405 112

BN0404 19

BN0403 25

BN0133 43

BN0132 53

BN0131 73

BN0130 155

BN0129 94

BN0128 38

BN0127 69

BN0126 114

BN0125 58

BN0124 16

BN0123 65

BN0121 223

BN0120 309

BN0118 57

**Library EST counts**

BN0117 91

BN0116 266

BN0115 1022

BN0114 607

BN0113 28

BN0112 63

BN0111 64

BN0109 58

BN0106 72

BN0105 161

BN0103 153

BN0102 150

BN0101 100

BN0098 57

BN0097 83

BN0096 169

BN0095 198

BN0094 2

BN0093 66

BN0091 74

BN0090 1030

BN0087 21

BN0085 302

BN0084 336

BN0083 544

BN0082 77

BN0081 61

BN0080 211

BN0078 45

BN0077 69

BN0075 182

BN0074 533

BN0073 44

BN0072 239

BN0070 2351

BN0068 49

**Library EST counts**

BN0067 243

BN0066 298

BN0065 64

BN0064 90

BN0063 302

BN0062 19

BN0060 24

BN0059 40

BN0058 30

BN0057 122

BN0056 132

BN0055 64

BN0054 164

BN0053 83

BN0052 98

BN0051 54

BN0050 161

BN0049 6

BN0048 251

BN0047 826

BN0046 849

BN0045 249

BN0044 260

BN0043 91

BN0042 1104

BN0041 235

BN0040 163

BN0039 512

BN0038 65

BN0037 139

BN0036 772

BN0035 123

BN0034 1119

BN0033 607

BN0032 218

BN0030 149

BN0026 132

BN0025 260

BN0024 164

BN0023 175

BN0022 128

BN0020 259

BN0015 56

BN0014 130

BN0013 54

BN0012 10

BN0011 76

BN0008 26

BN0007 305

BN0006 14

BN0005 547

BN0004 39

BN0003 140

BN0002 154

BN0001 142

AN0096 129

AN0095 43

AN0094 255

AN0093 428

**Library EST counts**

AN0092 36

AN0091 265

AN0090 46

AN0089 745

AN0088 34

AN0087 985

AN0086 244

AN0085 76

AN0084 262

AN0083 596

AN0082 117

AN0081 76

AN0080 1112

AN0079 14

AN0078 70

AN0077 109

AN0076 38

AN0075 35

AN0069 145

AN0068 262

AN0067 276

AN0066 53

AN0064 49

AN0063 131

AN0062 281

AN0061 33

AN0060 119

AN0059 177

AN0058 282

AN0056 67

AN0048 112

AN0047 133

AN0042 38

AN0041 507

AN0040 218

AN0039 437

AN0038 222

AN0037 372

AN0036 330

AN0034 102

AN0033 49

AN0032 103

AN0030 10

AN0029 153

AN0027 328

AN0026 11

AN0025 273

AN0024 47

AN0017 22

AN0016 138

AN0014 159

AN0013 48

AN0012 151

AN0007 235

AN0006 9

AN0005 30

AN0004 229

AN0003 41

AN0001 30
