# Supplementary material for: A novel method of differential gene expression analysis using multiple cDNA libraries applied to the identification of tumour endothelial genes
Source: BMC Genomics. 2008 Apr 7;9:153. doi: 10.1186/1471-2164-9-153 (PMC2346479; doi:10.1186/1471-2164-9-153)
Supplement: Additional file 3 — Comparisons of the most significantly differentially expressed genes using different statistical methods. [file 1471-2164-9-153-S3.doc]

**Additional file 3:** Comparisons of the most significantly differentially expressed genes using different statistical methods.

Parts 1 and 2 of this table show the top 10 most differentially expressed genes using q-values generated from different p-values. Part 1 shows the genes q-value ordered using a p-value generated from 2R which is asymptotically Chi Square distributed. Highly endothelial specific genes like ROBO4 appear in the top ten of this list. Part 2 shows genes q-value ordered using a p-value generated by a random randomization method. Although there are endothelial specific genes in the top ten, it is obvious from the EST counts that the Chi square q-values give a more biologically intuitive list of genes. Part 3 shows the top ten genes ordered using the Susko and Roger statistical approach. Part 4 shows the results for the endothelial gene TEK. The sample counts are low and as such should not produce a significant result. If q-values from the different methods are compared, the Chi squared q-value produces the less significant result. The Susko and Roger and posterior probabilities were insignificant for TEK. All analyses employed the Chi Square p-value generated q-values. All the parts of this table also show the posterior probability and the Susko and Roger statistics for each gene [20].

| **Part1** |  |  |  |  |  |  |  |  |  |  |  |  |
| --- | --- | --- | --- | --- | --- | --- | --- | --- | --- | --- | --- | --- |
| **Gene** | **Endo** | **NoneEndo** | **Chi square Rank** | **Chi Square qvalue** | **Boot strap rank** | **Boot strap qvalue** | **2fold Posterior prob.** | **Susko Roger Rank** | **p-value** | **Ben Hoch** | **Reject Null Hypoth.** | **reg.** |
| MCAM | 156 | 9 | 1 | 0 | 47 | 4.94E-05 | 1 | 7 | 0.00E+00 | 2.46E-05 | 1 | up |
| ENG | 149 | 2 | 2 | 0 | 111 | 4.94E-05 | 1 | 8 | 0.00E+00 | 2.87E-05 | 1 | up |
| APLP2 | 178 | 41 | 3 | 0 | 122 | 4.94E-05 | 1 | 25 | 0.00E+00 | 9.83E-05 | 1 | up |
| A2M | 143 | 3 | 4 | 0 | 135 | 4.94E-05 | 1 | 46 | 0.00E+00 | 1.84E-04 | 1 | up |
| MMP1 | 203 | 0 | 5 | 0 | 147 | 4.94E-05 | 1 | 32 | 0.00E+00 | 1.27E-04 | 1 | up |
| ROBO4 | 130 | 0 | 6 | 0 | 151 | 4.94E-05 | 1 | 19 | 0.00E+00 | 7.37E-05 | 1 | up |
| PRG1 | 159 | 15 | 7 | 0 | 168 | 4.94E-05 | 1 | 26 | 0.00E+00 | 1.02E-04 | 1 | up |
| CTGF | 202 | 58 | 8 | 0 | 174 | 4.94E-05 | 1 | 3 | 0.00E+00 | 8.19E-06 | 1 | up |
| TGM2 | 209 | 27 | 9 | 0 | 219 | 4.94E-05 | 1 | 21 | 0.00E+00 | 8.19E-05 | 1 | up |
| CA1 | 0 | 1163 | 10 | 0 | 245 | 4.94E-05 | 0.93 | 48 | 4.67E-129 | 1.92E-04 | 1 | down |
| **Part2** |  |  |  |  |  |  |  |  |  |  |  |  |
| **Gene** | **Endo** | **NoneEndo** | **Chi square Rank** | **Chi Square qvalue** | **Boot strap rank** | **Boot strap qvalue** | **2fold Posterior prob.** | **Susko Roger Rank** | **p-value** | **Ben Hoch** | **Reject Null Hypoth.** | **reg.** |
| MMP2 | 19 | 11 | 222 | 3.77E-06 | 1 | 4.94E-05 | 0.99 | 215 | 3.81E-06 | 1.01E-03 | 1 | up |
| EDN1 | 16 | 3 | 160 | 4.04E-08 | 2 | 4.94E-05 | 1 | 160 | 4.05E-08 | 6.88E-04 | 1 | up |
| RPS18 | 66 | 99 | 144 | 1.35E-08 | 3 | 4.94E-05 | 0.74 | 193 | 6.91E-07 | 8.72E-04 | 1 | up |
| TMSB10 | 45 | 31 | 77 | 6.74E-13 | 4 | 4.94E-05 | 1 | 96 | 1.21E-11 | 4.01E-04 | 1 | up |
| ERG | 16 | 0 | 96 | 2.64E-11 | 5 | 4.94E-05 | 1 | 115 | 8.53E-11 | 4.83E-04 | 1 | up |
| NOSTRIN | 9 | 0 | 208 | 2.09E-06 | 6 | 4.94E-05 | 0.99 | 211 | 2.94E-06 | 9.58E-04 | 1 | up |
| COL1A1 | 1 | 99 | 187 | 6.69E-07 | 7 | 4.94E-05 | 1 | 125 | 5.22E-10 | 5.32E-04 | 1 | down |
| RNF4 | 14 | 3 | 191 | 7.35E-07 | 8 | 4.94E-05 | 1 | 189 | 5.66E-07 | 8.35E-04 | 1 | up |
| SEC14L1 | 14 | 1 | 149 | 1.85E-08 | 9 | 4.94E-05 | 1 | 154 | 2.00E-08 | 6.55E-04 | 1 | up |
| RGS4 | 51 | 11 | 38 | 4.57E-25 | 10 | 4.94E-05 | 1 | 31 | 0.00E+00 | 1.23E-04 | 1 | up |
| **Part3** |  |  |  |  |  |  |  |  |  |  |  |  |
| SPARCL1 | 97 | 0 | 11 | 0.0000 | 179 | 0.0000 | 1 | 1 | 0.00E+00 | 4.09E-06 | 1 | up |
| NCL | 85 | 30 | 27 | 0.0000 | 28 | 0.0000 | 1 | 2 | 0.00E+00 | 4.09E-06 | 1 | up |
| CTGF | 202 | 58 | 8 | 0.0000 | 174 | 0.0000 | 1 | 3 | 0.00E+00 | 8.19E-06 | 1 | up |
| UNC45A | 34 | 5 | 55 | 0.0000 | 130 | 0.0000 | 1 | 4 | 0.00E+00 | 1.23E-05 | 1 | up |
| RHOJ | 31 | 0 | 42 | 0.0000 | 154 | 0.0000 | 1 | 5 | 0.00E+00 | 1.64E-05 | 1 | up |
| EMP1 | 136 | 30 | 13 | 0.0000 | 225 | 0.0000 | 1 | 6 | 0.00E+00 | 2.05E-05 | 1 | up |
| MCAM | 156 | 9 | 1 | 0.0000 | 47 | 0.0000 | 1 | 7 | 0.00E+00 | 2.46E-05 | 1 | up |
| ENG | 149 | 2 | 2 | 0.0000 | 111 | 0.0000 | 1 | 8 | 0.00E+00 | 2.87E-05 | 1 | up |
| HYOU1 | 43 | 11 | 48 | 0.0000 | 72 | 0.0000 | 1 | 9 | 0.00E+00 | 3.28E-05 | 1 | up |
| ITGA5 | 113 | 15 | 14 | 0.0000 | 131 | 0.0000 | 1 | 10 | 0.00E+00 | 3.68E-05 | 1 | up |
| **Part4** |  |  |  |  |  |  |  |  |  |  |  |  |
| **Gene** | **Endo** | **NoneEndo** | **Chi square Rank** | **Chi Square qvalue** | **Boot strap rank** | **Boot strap qvalue** | **2fold Posterior prob.** | **Susko Roger Rank** | **p-value** | **Ben Hoch** | **Reject Null Hypoth.** | **reg.** |
| TEK | 4 | 0 | 476 | 0.005884 | 420 | 0.001633 | 0.78 | 393 | 5.11E-03 | 2.54E-03 | 0 | up |
