# Supplementary material for: A novel method of differential gene expression analysis using multiple cDNA libraries applied to the identification of tumour endothelial genes
Source: BMC Genomics. 2008 Apr 7;9:153. doi: 10.1186/1471-2164-9-153 (PMC2346479; doi:10.1186/1471-2164-9-153)
Supplement: Additional file 5 — 554 genes were found to be significantly up or down regulated in endothelial cells based on a q-value <= 0.01 generated using a Chi Square p-value. randomization q-values (Stekel et al. 2000), Susko and Roger statistics and posterior probabilities are also displayed for comparison. [file 1471-2164-9-153-S5.doc]

**Additional file 5:** 554 genes were found to be significantly up or down regulated in endothelial cells based on a q-value <= 0.01 generated using a chi square p-value. Susko and Roger statistics, randomization q-values and posterior probabilities are also displayed for comparison.

| **Gene** | **Endo Count** | **NonEndo Count** | **Chi square Rank** | **Chi square qvalue** | **Random rank** | **Random qvalue** | **2fold Posterior Probability** | **Susko Roger rank** | **Susko Roger pvalue** | **Susko Roger ben/hoch** | **Susko Roger reject null** | **Up/Down reg.** |
| --- | --- | --- | --- | --- | --- | --- | --- | --- | --- | --- | --- | --- |
| MCAM | 156 | 9 | 1 | 0 | 47 | 4.94E-05 | 1 | 7 | 0.00E+00 | 2.46E-05 | 1 | UP |
| ENG | 149 | 2 | 2 | 0 | 111 | 4.94E-05 | 1 | 8 | 0.00E+00 | 2.87E-05 | 1 | UP |
| APLP2 | 178 | 41 | 3 | 0 | 122 | 4.94E-05 | 1 | 25 | 0.00E+00 | 9.83E-05 | 1 | UP |
| A2M | 143 | 3 | 4 | 0 | 135 | 4.94E-05 | 1 | 46 | 0.00E+00 | 1.84E-04 | 1 | UP |
| MMP1 | 203 | 0 | 5 | 0 | 147 | 4.94E-05 | 1 | 32 | 0.00E+00 | 1.27E-04 | 1 | UP |
| ROBO4 | 130 | 0 | 6 | 0 | 151 | 4.94E-05 | 1 | 19 | 0.00E+00 | 7.37E-05 | 1 | UP |
| PRG1 | 159 | 15 | 7 | 0 | 168 | 4.94E-05 | 1 | 26 | 0.00E+00 | 1.02E-04 | 1 | UP |
| CTGF | 202 | 58 | 8 | 0 | 174 | 4.94E-05 | 1 | 3 | 0.00E+00 | 8.19E-06 | 1 | UP |
| TGM2 | 209 | 27 | 9 | 0 | 219 | 4.94E-05 | 1 | 21 | 0.00E+00 | 8.19E-05 | 1 | UP |
| CA1 | 0 | 1163 | 10 | 0 | 245 | 4.94E-05 | 0.93 | 48 | 4.67E-129 | 1.92E-04 | 1 | down |
| SPARCL1 | 97 | 0 | 11 | 5.21E-70 | 179 | 4.94E-05 | 1 | 1 | 0.00E+00 | 4.09E-06 | 1 | UP |
| HDLBP | 164 | 61 | 12 | 2.15E-67 | 157 | 4.94E-05 | 1 | 17 | 0.00E+00 | 6.55E-05 | 1 | UP |
| EMP1 | 136 | 30 | 13 | 3.22E-67 | 225 | 4.94E-05 | 1 | 6 | 0.00E+00 | 2.05E-05 | 1 | UP |
| ITGA5 | 113 | 15 | 14 | 4.73E-63 | 131 | 4.94E-05 | 1 | 10 | 0.00E+00 | 3.68E-05 | 1 | UP |
| CD74 | 6 | 819 | 15 | 4.39E-61 | 90 | 4.94E-05 | 1 | 49 | 2.97E-80 | 1.97E-04 | 1 | down |
| EIF4G2 | 156 | 73 | 16 | 1.50E-57 | 128 | 4.94E-05 | 1 | 16 | 0.00E+00 | 6.14E-05 | 1 | UP |
| HSPA8 | 251 | 243 | 17 | 8.46E-56 | 74 | 4.94E-05 | 1 | 14 | 0.00E+00 | 5.32E-05 | 1 | UP |
| VWF | 73 | 0 | 18 | 1.33E-52 | 228 | 4.94E-05 | 1 | 41 | 0.00E+00 | 1.64E-04 | 1 | UP |
| VIM | 209 | 181 | 19 | 2.73E-51 | 167 | 4.94E-05 | 1 | 36 | 0.00E+00 | 1.43E-04 | 1 | UP |
| BGN | 82 | 6 | 20 | 3.03E-50 | 145 | 4.94E-05 | 1 | 18 | 0.00E+00 | 6.96E-05 | 1 | UP |
| SGK | 86 | 9 | 21 | 4.82E-50 | 202 | 4.94E-05 | 1 | 42 | 0.00E+00 | 1.68E-04 | 1 | UP |
| CAV1 | 80 | 8 | 22 | 7.41E-47 | 177 | 4.94E-05 | 1 | 47 | 0.00E+00 | 1.88E-04 | 1 | UP |
| NR_002819 | 3 | 587 | 23 | 1.38E-45 | 58 | 4.94E-05 | 1 | 50 | 9.38E-60 | 2.01E-04 | 1 | down |
| HHIP | 61 | 0 | 24 | 6.58E-44 | 241 | 4.94E-05 | 1 | 38 | 0.00E+00 | 1.51E-04 | 1 | UP |
| SERPINE1 | 97 | 30 | 25 | 8.81E-43 | 126 | 4.94E-05 | 1 | 34 | 0.00E+00 | 1.35E-04 | 1 | UP |
| PTRF | 98 | 34 | 26 | 2.51E-41 | 136 | 4.94E-05 | 1 | 37 | 0.00E+00 | 1.47E-04 | 1 | UP |
| NCL | 85 | 30 | 27 | 1.76E-35 | 28 | 4.94E-05 | 1 | 2 | 0.00E+00 | 4.09E-06 | 1 | UP |
| S100A8 | 48 | 1 | 28 | 2.28E-32 | 48 | 4.94E-05 | 1 | 11 | 0.00E+00 | 4.09E-05 | 1 | UP |
| LOC441763 | 1 | 382 | 29 | 4.87E-31 | 67 | 4.94E-05 | 1 | 52 | 9.76E-41 | 2.09E-04 | 1 | down |
| RHOB | 54 | 6 | 30 | 7.14E-31 | 118 | 4.94E-05 | 1 | 27 | 0.00E+00 | 1.06E-04 | 1 | UP |
| DYSF | 48 | 3 | 31 | 9.74E-30 | 190 | 4.94E-05 | 1 | 28 | 0.00E+00 | 1.11E-04 | 1 | UP |
| ENPP2 | 43 | 1 | 32 | 8.63E-29 | 200 | 4.94E-05 | 1 | 23 | 0.00E+00 | 9.01E-05 | 1 | UP |
| FTL | 31 | 695 | 33 | 2.65E-28 | 166 | 4.94E-05 | 1 | 51 | 8.00E-43 | 2.05E-04 | 1 | down |
| CYCS | 1 | 338 | 34 | 3.27E-27 | 68 | 4.94E-05 | 1 | 53 | 6.36E-36 | 2.13E-04 | 1 | down |
| DCTN1 | 61 | 20 | 35 | 4.40E-26 | 12 | 4.94E-05 | 1 | 29 | 0.00E+00 | 1.15E-04 | 1 | UP |
| PECAM1 | 39 | 1 | 36 | 6.19E-26 | 53 | 4.94E-05 | 1 | 22 | 0.00E+00 | 8.60E-05 | 1 | UP |
| TMSB4X | 120 | 121 | 37 | 2.20E-25 | 129 | 4.94E-05 | 1 | 35 | 0.00E+00 | 1.39E-04 | 1 | UP |
| RGS4 | 51 | 11 | 38 | 4.57E-25 | 10 | 4.94E-05 | 1 | 31 | 0.00E+00 | 1.23E-04 | 1 | UP |
| GRN | 57 | 19 | 39 | 3.37E-24 | 125 | 4.94E-05 | 1 | 45 | 0.00E+00 | 1.80E-04 | 1 | UP |
| RNF40 | 46 | 8 | 40 | 6.68E-24 | 240 | 4.94E-05 | 1 | 33 | 0.00E+00 | 1.31E-04 | 1 | UP |
| C9orf26 | 33 | 0 | 41 | 1.60E-23 | 150 | 4.94E-05 | 1 | 30 | 0.00E+00 | 1.19E-04 | 1 | UP |
| RHOJ | 31 | 0 | 42 | 4.68E-22 | 154 | 4.94E-05 | 1 | 5 | 0.00E+00 | 1.64E-05 | 1 | UP |
| S100A6 | 58 | 25 | 43 | 5.42E-22 | 232 | 4.94E-05 | 1 | 43 | 0.00E+00 | 1.72E-04 | 1 | UP |
| BMX | 30 | 0 | 44 | 2.45E-21 | 213 | 4.94E-05 | 1 | 12 | 0.00E+00 | 4.50E-05 | 1 | UP |
| EFEMP1 | 47 | 13 | 45 | 2.52E-21 | 86 | 4.94E-05 | 1 | 15 | 0.00E+00 | 5.73E-05 | 1 | UP |
| GAPDH | 50 | 737 | 46 | 8.52E-21 | 215 | 4.94E-05 | 1 | 54 | 1.31E-34 | 2.17E-04 | 1 | down |
| ELTD1 | 29 | 0 | 47 | 1.26E-20 | 212 | 4.94E-05 | 1 | 13 | 0.00E+00 | 4.91E-05 | 1 | UP |
| HYOU1 | 43 | 11 | 48 | 5.34E-20 | 72 | 4.94E-05 | 1 | 9 | 0.00E+00 | 3.28E-05 | 1 | UP |
| RPL26 | 76 | 60 | 49 | 7.66E-20 | 175 | 4.94E-05 | 1 | 20 | 0.00E+00 | 7.78E-05 | 1 | UP |
| B2M | 26 | 515 | 50 | 4.83E-19 | 15 | 4.94E-05 | 1 | 55 | 5.80E-30 | 2.21E-04 | 1 | down |
| ANXA2 | 178 | 304 | 51 | 6.84E-19 | 18 | 4.94E-05 | 0.49 | 85 | 1.03E-12 | 3.48E-04 | 1 | UP |
| ESM1 | 29 | 1 | 52 | 7.74E-19 | 159 | 4.94E-05 | 1 | 24 | 0.00E+00 | 9.42E-05 | 1 | UP |
| MMRN1 | 26 | 0 | 53 | 1.84E-18 | 183 | 4.94E-05 | 1 | 40 | 0.00E+00 | 1.60E-04 | 1 | UP |
| ZNF207 | 47 | 19 | 54 | 2.54E-18 | 214 | 4.94E-05 | 1 | 39 | 0.00E+00 | 1.56E-04 | 1 | UP |
| UNC45A | 34 | 5 | 55 | 2.74E-18 | 130 | 4.94E-05 | 1 | 4 | 0.00E+00 | 1.23E-05 | 1 | UP |
| CALM2 | 20 | 444 | 56 | 7.60E-18 | 85 | 4.94E-05 | 1 | 56 | 1.17E-27 | 2.25E-04 | 1 | down |
| EMCN | 24 | 0 | 57 | 5.17E-17 | 203 | 4.94E-05 | 1 | 44 | 0.00E+00 | 1.76E-04 | 1 | UP |
| RPS27A | 60 | 43 | 58 | 8.53E-17 | 101 | 4.94E-05 | 1 | 62 | 2.22E-16 | 2.50E-04 | 1 | UP |
| CDH5 | 23 | 0 | 59 | 2.75E-16 | 186 | 4.94E-05 | 1 | 61 | 2.22E-16 | 2.46E-04 | 1 | UP |
| LMNA | 53 | 34 | 60 | 5.46E-16 | 65 | 4.94E-05 | 1 | 71 | 2.26E-14 | 2.91E-04 | 1 | UP |
| WARS | 39 | 14 | 61 | 5.81E-16 | 104 | 4.94E-05 | 1 | 65 | 3.11E-15 | 2.62E-04 | 1 | UP |
| RPN1 | 36 | 11 | 62 | 1.10E-15 | 139 | 4.94E-05 | 1 | 67 | 6.00E-15 | 2.70E-04 | 1 | UP |
| GNAI3 | 39 | 15 | 63 | 1.72E-15 | 162 | 4.94E-05 | 1 | 70 | 1.75E-14 | 2.87E-04 | 1 | UP |
| PRDX2 | 3 | 233 | 64 | 1.86E-15 | 64 | 4.94E-05 | 1 | 58 | 8.72E-22 | 2.33E-04 | 1 | down |
| VAMP3 | 35 | 11 | 65 | 4.39E-15 | 124 | 4.94E-05 | 1 | 74 | 3.26E-14 | 3.03E-04 | 1 | UP |
| NDRG1 | 33 | 9 | 66 | 5.85E-15 | 23 | 4.94E-05 | 1 | 75 | 3.57E-14 | 3.07E-04 | 1 | UP |
| RPL19 | 61 | 52 | 67 | 6.71E-15 | 132 | 4.94E-05 | 1 | 84 | 7.27E-13 | 3.44E-04 | 1 | UP |
| ADAM15 | 25 | 2 | 68 | 7.11E-15 | 246 | 4.94E-05 | 1 | 73 | 2.38E-14 | 2.99E-04 | 1 | UP |
| SOX7 | 20 | 0 | 69 | 3.94E-14 | 161 | 4.94E-05 | 1 | 78 | 2.16E-13 | 3.19E-04 | 1 | UP |
| RPL37A | 59 | 52 | 70 | 5.52E-14 | 210 | 4.94E-05 | 1 | 90 | 4.26E-12 | 3.68E-04 | 1 | UP |
| PLOD1 | 33 | 11 | 71 | 6.93E-14 | 217 | 4.94E-05 | 1 | 80 | 4.15E-13 | 3.28E-04 | 1 | UP |
| ECE1 | 32 | 10 | 72 | 8.38E-14 | 196 | 4.94E-05 | 1 | 81 | 4.55E-13 | 3.32E-04 | 1 | UP |
| LOC651928 | 1 | 182 | 73 | 9.06E-14 | 201 | 4.94E-05 | 1 | 59 | 6.22E-19 | 2.37E-04 | 1 | down |
| OS9 | 29 | 7 | 74 | 1.16E-13 | 60 | 4.94E-05 | 1 | 82 | 4.77E-13 | 3.36E-04 | 1 | UP |
| XRCC6 | 60 | 58 | 75 | 4.52E-13 | 165 | 4.94E-05 | 1 | 103 | 3.27E-11 | 4.34E-04 | 1 | UP |
| DYNLL1 | 33 | 13 | 76 | 6.27E-13 | 91 | 4.94E-05 | 1 | 88 | 3.40E-12 | 3.60E-04 | 1 | UP |
| TMSB10 | 45 | 31 | 77 | 6.74E-13 | 4 | 4.94E-05 | 1 | 96 | 1.21E-11 | 4.01E-04 | 1 | UP |
| C4orf18 | 22 | 2 | 78 | 8.15E-13 | 11 | 4.94E-05 | 1 | 86 | 1.87E-12 | 3.52E-04 | 1 | UP |
| PCDH12 | 18 | 0 | 79 | 1.03E-12 | 19 | 4.94E-05 | 1 | 91 | 4.31E-12 | 3.73E-04 | 1 | UP |
| ARHGAP24 | 18 | 0 | 80 | 1.03E-12 | 35 | 4.94E-05 | 1 | 91 | 4.31E-12 | 3.73E-04 | 1 | UP |
| FGD5 | 18 | 0 | 81 | 1.03E-12 | 63 | 4.94E-05 | 1 | 91 | 4.31E-12 | 3.73E-04 | 1 | UP |
| CALU | 49 | 39 | 82 | 1.24E-12 | 117 | 4.94E-05 | 1 | 102 | 3.26E-11 | 4.30E-04 | 1 | UP |
| SERPINH1 | 44 | 31 | 83 | 2.02E-12 | 114 | 4.94E-05 | 1 | 101 | 3.19E-11 | 4.26E-04 | 1 | UP |
| FLNB | 39 | 23 | 84 | 2.21E-12 | 229 | 4.94E-05 | 1 | 99 | 2.15E-11 | 4.13E-04 | 1 | UP |
| GJA1 | 37 | 21 | 85 | 5.23E-12 | 218 | 4.94E-05 | 1 | 106 | 3.94E-11 | 4.46E-04 | 1 | UP |
| CD93 | 17 | 0 | 86 | 5.37E-12 | 142 | 4.94E-05 | 1 | 98 | 1.92E-11 | 4.09E-04 | 1 | UP |
| CORO1A | 0 | 139 | 87 | 6.00E-12 | 59 | 4.94E-05 | 1 | 64 | 8.46E-16 | 2.58E-04 | 1 | down |
| PPP2R1A | 43 | 31 | 88 | 6.12E-12 | 119 | 4.94E-05 | 1 | 114 | 8.38E-11 | 4.79E-04 | 1 | UP |
| PLS3 | 36 | 20 | 89 | 7.67E-12 | 83 | 4.94E-05 | 1 | 108 | 5.30E-11 | 4.54E-04 | 1 | UP |
| WDR6 | 32 | 15 | 90 | 1.54E-11 | 73 | 4.94E-05 | 1 | 110 | 6.88E-11 | 4.63E-04 | 1 | UP |
| SLC29A1 | 20 | 2 | 91 | 1.77E-11 | 208 | 4.94E-05 | 1 | 100 | 3.11E-11 | 4.18E-04 | 1 | UP |
| MANSC1 | 20 | 2 | 92 | 1.77E-11 | 211 | 4.94E-05 | 1 | 100 | 3.11E-11 | 4.18E-04 | 1 | UP |
| RPS20 | 64 | 75 | 93 | 1.94E-11 | 235 | 4.94E-05 | 0.98 | 133 | 1.53E-09 | 5.65E-04 | 1 | UP |
| IGHG1 | 0 | 133 | 94 | 1.94E-11 | 92 | 4.94E-05 | 1 | 66 | 3.90E-15 | 2.66E-04 | 1 | down |
| GANAB | 37 | 23 | 95 | 2.30E-11 | 76 | 4.94E-05 | 1 | 118 | 1.69E-10 | 4.99E-04 | 1 | UP |
| ERG | 16 | 0 | 96 | 2.64E-11 | 5 | 4.94E-05 | 1 | 115 | 8.53E-11 | 4.83E-04 | 1 | UP |
| MYCT1 | 16 | 0 | 97 | 2.64E-11 | 226 | 4.94E-05 | 1 | 115 | 8.53E-11 | 4.83E-04 | 1 | UP |
| KRT19 | 0 | 131 | 98 | 2.83E-11 | 209 | 4.94E-05 | 1 | 68 | 6.49E-15 | 2.74E-04 | 1 | down |
| TPD52L2 | 25 | 7 | 99 | 3.21E-11 | 163 | 4.94E-05 | 1 | 111 | 7.67E-11 | 4.67E-04 | 1 | UP |
| PRCP | 24 | 6 | 100 | 3.49E-11 | 98 | 4.94E-05 | 1 | 112 | 7.70E-11 | 4.71E-04 | 1 | UP |
| NONO | 61 | 70 | 101 | 3.50E-11 | 32 | 4.94E-05 | 0.98 | 137 | 2.16E-09 | 5.81E-04 | 1 | UP |
| RPAP1 | 18 | 1 | 102 | 3.56E-11 | 17 | 4.94E-05 | 1 | 109 | 6.44E-11 | 4.59E-04 | 1 | UP |
| DYNC1I2 | 29 | 12 | 103 | 3.82E-11 | 107 | 4.94E-05 | 1 | 117 | 1.34E-10 | 4.95E-04 | 1 | UP |
| C15orf15 | 0 | 129 | 104 | 4.01E-11 | 21 | 4.94E-05 | 0.92 | 69 | 1.08E-14 | 2.78E-04 | 1 | down |
| DC2 | 0 | 129 | 105 | 4.01E-11 | 49 | 4.94E-05 | 0.92 | 69 | 1.08E-14 | 2.78E-04 | 1 | down |
| MYO1C | 27 | 10 | 106 | 6.74E-11 | 164 | 4.94E-05 | 1 | 119 | 1.92E-10 | 5.04E-04 | 1 | UP |
| HBB | 0 | 126 | 107 | 7.37E-11 | 29 | 4.94E-05 | 1 | 72 | 2.32E-14 | 2.95E-04 | 1 | down |
| RPL31 | 55 | 59 | 108 | 7.50E-11 | 34 | 4.94E-05 | 0.99 | 139 | 2.77E-09 | 5.90E-04 | 1 | UP |
| TNPO1 | 19 | 2 | 109 | 7.50E-11 | 149 | 4.94E-05 | 1 | 116 | 1.26E-10 | 4.91E-04 | 1 | UP |
| WDR1 | 45 | 39 | 110 | 7.52E-11 | 221 | 4.94E-05 | 1 | 130 | 1.19E-09 | 5.53E-04 | 1 | UP |
| FLJ22746 | 15 | 0 | 111 | 1.28E-10 | 95 | 4.94E-05 | 1 | 123 | 3.79E-10 | 5.24E-04 | 1 | UP |
| IKBKE | 21 | 4 | 112 | 1.34E-10 | 181 | 4.94E-05 | 1 | 120 | 2.36E-10 | 5.08E-04 | 1 | UP |
| SMG6 | 21 | 4 | 113 | 1.34E-10 | 182 | 4.94E-05 | 1 | 120 | 2.36E-10 | 5.08E-04 | 1 | UP |
| SPRR3 | 17 | 1 | 114 | 1.67E-10 | 243 | 4.94E-05 | 1 | 121 | 2.72E-10 | 5.16E-04 | 1 | UP |
| RPL32 | 37 | 26 | 115 | 1.69E-10 | 192 | 4.94E-05 | 1 | 131 | 1.23E-09 | 5.57E-04 | 1 | UP |
| ACTG1 | 110 | 928 | 116 | 1.86E-10 | 97 | 4.94E-05 | 0.97 | 57 | 3.72E-23 | 2.29E-04 | 1 | down |
| ALB | 0 | 120 | 117 | 2.36E-10 | 36 | 4.94E-05 | 1 | 77 | 1.07E-13 | 3.15E-04 | 1 | down |
| CYR61 | 37 | 27 | 118 | 3.26E-10 | 39 | 4.94E-05 | 1 | 138 | 2.29E-09 | 5.85E-04 | 1 | UP |
| DUSP6 | 19 | 3 | 119 | 5.03E-10 | 55 | 4.94E-05 | 1 | 127 | 7.26E-10 | 5.40E-04 | 1 | UP |
| UBC | 89 | 147 | 120 | 5.77E-10 | 187 | 4.94E-05 | 0.55 | 180 | 1.88E-07 | 7.94E-04 | 1 | UP |
| SELE | 14 | 0 | 121 | 6.56E-10 | 45 | 4.94E-05 | 1 | 134 | 1.69E-09 | 5.69E-04 | 1 | UP |
| C10orf10 | 16 | 1 | 122 | 8.22E-10 | 172 | 4.94E-05 | 1 | 129 | 1.14E-09 | 5.49E-04 | 1 | UP |
| LOC647099 | 2 | 149 | 123 | 8.60E-10 | 134 | 4.94E-05 | 1 | 76 | 3.97E-14 | 3.11E-04 | 1 | down |
| FAM62A | 25 | 10 | 124 | 9.20E-10 | 105 | 4.94E-05 | 1 | 136 | 2.02E-09 | 5.77E-04 | 1 | UP |
| RPS12 | 42 | 38 | 125 | 9.39E-10 | 176 | 4.94E-05 | 0.99 | 148 | 9.79E-09 | 6.30E-04 | 1 | UP |
| ACTR3 | 30 | 18 | 126 | 1.95E-09 | 40 | 4.94E-05 | 1 | 144 | 6.43E-09 | 6.10E-04 | 1 | UP |
| MMS19L | 21 | 6 | 127 | 2.30E-09 | 239 | 4.94E-05 | 1 | 140 | 3.41E-09 | 5.94E-04 | 1 | UP |
| SDCBP | 25 | 11 | 128 | 2.51E-09 | 56 | 4.94E-05 | 1 | 142 | 5.19E-09 | 6.02E-04 | 1 | UP |
| TXNDC5 | 34 | 25 | 129 | 2.52E-09 | 231 | 4.94E-05 | 1 | 150 | 1.20E-08 | 6.39E-04 | 1 | UP |
| KIAA0174 | 35 | 27 | 130 | 2.94E-09 | 223 | 4.94E-05 | 1 | 152 | 1.52E-08 | 6.47E-04 | 1 | UP |
| TCF4 | 13 | 0 | 131 | 3.36E-09 | 193 | 4.94E-05 | 1 | 145 | 7.50E-09 | 6.14E-04 | 1 | UP |
| ANGPT2 | 13 | 0 | 132 | 3.36E-09 | 234 | 4.94E-05 | 1 | 145 | 7.50E-09 | 6.14E-04 | 1 | UP |
| CA2 | 0 | 105 | 133 | 4.79E-09 | 123 | 4.94E-05 | 1 | 92 | 4.87E-12 | 3.85E-04 | 1 | down |
| RPS8 | 69 | 103 | 134 | 4.79E-09 | 103 | 4.94E-05 | 0.75 | 186 | 3.34E-07 | 8.19E-04 | 1 | UP |
| THBS1 | 40 | 38 | 135 | 7.11E-09 | 152 | 4.94E-05 | 0.99 | 164 | 5.36E-08 | 7.08E-04 | 1 | UP |
| TIE1 | 16 | 2 | 136 | 7.50E-09 | 170 | 4.94E-05 | 1 | 147 | 8.11E-09 | 6.26E-04 | 1 | UP |
| DAB2 | 21 | 7 | 137 | 7.57E-09 | 113 | 4.94E-05 | 1 | 149 | 1.07E-08 | 6.35E-04 | 1 | UP |
| RPS24 | 53 | 66 | 138 | 7.60E-09 | 84 | 4.94E-05 | 0.93 | 177 | 1.64E-07 | 7.78E-04 | 1 | UP |
| SPARC | 52 | 64 | 139 | 8.41E-09 | 184 | 4.94E-05 | 0.93 | 179 | 1.67E-07 | 7.90E-04 | 1 | UP |
| SERPINA3 | 0 | 102 | 140 | 8.57E-09 | 38 | 4.94E-05 | 1 | 95 | 1.05E-11 | 3.97E-04 | 1 | down |
| BNIP3L | 3 | 148 | 141 | 1.08E-08 | 94 | 4.94E-05 | 1 | 83 | 5.86E-13 | 3.40E-04 | 1 | down |
| GPI | 38 | 35 | 142 | 1.11E-08 | 100 | 4.94E-05 | 0.99 | 166 | 7.02E-08 | 7.16E-04 | 1 | UP |
| C11orf58 | 0 | 100 | 143 | 1.28E-08 | 224 | 4.94E-05 | 1 | 97 | 1.74E-11 | 4.05E-04 | 1 | down |
| RPS18 | 66 | 99 | 144 | 1.35E-08 | 3 | 4.94E-05 | 0.74 | 193 | 6.91E-07 | 8.72E-04 | 1 | UP |
| MYH9 | 2 | 133 | 145 | 1.66E-08 | 222 | 4.94E-05 | 1 | 87 | 1.88E-12 | 3.56E-04 | 1 | down |
| TINAGL1 | 22 | 9 | 146 | 1.67E-08 | 81 | 4.94E-05 | 1 | 155 | 2.52E-08 | 6.59E-04 | 1 | UP |
| EDG1 | 12 | 0 | 147 | 1.68E-08 | 158 | 4.94E-05 | 1 | 156 | 3.34E-08 | 6.63E-04 | 1 | UP |
| SDPR | 12 | 0 | 148 | 1.68E-08 | 236 | 4.94E-05 | 1 | 156 | 3.34E-08 | 6.63E-04 | 1 | UP |
| SEC14L1 | 14 | 1 | 149 | 1.85E-08 | 9 | 4.94E-05 | 1 | 154 | 2.00E-08 | 6.55E-04 | 1 | UP |
| TMBIM1 | 26 | 15 | 150 | 2.16E-08 | 20 | 4.94E-05 | 1 | 163 | 4.65E-08 | 7.04E-04 | 1 | UP |
| PPIA | 96 | 182 | 151 | 2.27E-08 | 230 | 4.94E-05 | 0.18 | 221 | 6.26E-06 | 1.04E-03 | 1 | UP |
| VAT1 | 29 | 20 | 152 | 2.43E-08 | 16 | 4.94E-05 | 1 | 165 | 6.85E-08 | 7.12E-04 | 1 | UP |
| RPS3A | 62 | 581 | 153 | 2.67E-08 | 24 | 4.94E-05 | 0.99 | 60 | 2.20E-17 | 2.42E-04 | 1 | down |
| EXOSC10 | 20 | 7 | 154 | 2.78E-08 | 43 | 4.94E-05 | 1 | 158 | 3.59E-08 | 6.76E-04 | 1 | UP |
| NRP1 | 20 | 7 | 155 | 2.78E-08 | 99 | 4.94E-05 | 1 | 158 | 3.59E-08 | 6.76E-04 | 1 | UP |
| SNAP23 | 18 | 5 | 156 | 4.01E-08 | 115 | 4.94E-05 | 1 | 162 | 4.39E-08 | 6.96E-04 | 1 | UP |
| TYK2 | 18 | 5 | 157 | 4.01E-08 | 205 | 4.94E-05 | 1 | 162 | 4.39E-08 | 6.96E-04 | 1 | UP |
| SRM | 2 | 128 | 158 | 4.03E-08 | 33 | 4.94E-05 | 1 | 93 | 6.23E-12 | 3.89E-04 | 1 | down |
| IMPDH2 | 0 | 94 | 159 | 4.04E-08 | 189 | 4.94E-05 | 1 | 113 | 8.02E-11 | 4.75E-04 | 1 | down |
| EDN1 | 16 | 3 | 160 | 4.04E-08 | 2 | 4.94E-05 | 1 | 160 | 4.05E-08 | 6.88E-04 | 1 | UP |
| TFPI2 | 24 | 13 | 161 | 4.77E-08 | 66 | 4.94E-05 | 1 | 169 | 8.49E-08 | 7.33E-04 | 1 | UP |
| DDX5 | 50 | 65 | 162 | 6.27E-08 | 133 | 4.94E-05 | 0.88 | 197 | 9.27E-07 | 8.88E-04 | 1 | UP |
| RPS15A | 34 | 31 | 163 | 7.54E-08 | 57 | 4.94E-05 | 0.98 | 184 | 3.11E-07 | 8.11E-04 | 1 | UP |
| SERINC3 | 20 | 8 | 164 | 8.07E-08 | 41 | 4.94E-05 | 1 | 170 | 9.90E-08 | 7.37E-04 | 1 | UP |
| POLE3 | 1 | 110 | 165 | 8.32E-08 | 71 | 4.94E-05 | 1 | 105 | 3.51E-11 | 4.42E-04 | 1 | down |
| ARHGEF15 | 11 | 0 | 166 | 8.36E-08 | 89 | 4.94E-05 | 1 | 173 | 1.48E-07 | 7.53E-04 | 1 | UP |
| GIMAP6 | 11 | 0 | 167 | 8.36E-08 | 143 | 4.94E-05 | 1 | 173 | 1.48E-07 | 7.53E-04 | 1 | UP |
| COL3A1 | 0 | 89 | 168 | 1.10E-07 | 50 | 4.94E-05 | 1 | 122 | 2.87E-10 | 5.20E-04 | 1 | down |
| BAG3 | 18 | 6 | 169 | 1.33E-07 | 198 | 4.94E-05 | 1 | 172 | 1.38E-07 | 7.45E-04 | 1 | UP |
| F2R | 18 | 6 | 170 | 1.33E-07 | 207 | 4.94E-05 | 1 | 172 | 1.38E-07 | 7.45E-04 | 1 | UP |
| UBE2V2 | 2 | 121 | 171 | 1.45E-07 | 185 | 4.94E-05 | 1 | 104 | 3.32E-11 | 4.38E-04 | 1 | down |
| RPS26 | 21 | 10 | 172 | 1.49E-07 | 106 | 4.94E-05 | 1 | 181 | 1.95E-07 | 7.98E-04 | 1 | UP |
| ADSS | 17 | 5 | 173 | 1.57E-07 | 155 | 4.94E-05 | 1 | 175 | 1.53E-07 | 7.66E-04 | 1 | UP |
| ALDH1A1 | 17 | 5 | 174 | 1.57E-07 | 178 | 4.94E-05 | 1 | 175 | 1.53E-07 | 7.66E-04 | 1 | UP |
| GPR177 | 16 | 4 | 175 | 1.75E-07 | 26 | 4.94E-05 | 1 | 176 | 1.60E-07 | 7.74E-04 | 1 | UP |
| HNRPH1 | 26 | 18 | 176 | 1.81E-07 | 195 | 4.94E-05 | 0.99 | 187 | 3.65E-07 | 8.23E-04 | 1 | UP |
| POSTN | 19 | 8 | 177 | 2.96E-07 | 204 | 4.94E-05 | 1 | 185 | 3.16E-07 | 8.15E-04 | 1 | UP |
| SDHD | 4 | 139 | 178 | 3.75E-07 | 141 | 4.94E-05 | 1 | 107 | 3.99E-11 | 4.50E-04 | 1 | down |
| PLA2G4C | 10 | 0 | 179 | 4.33E-07 | 14 | 4.94E-05 | 1 | 192 | 6.60E-07 | 8.56E-04 | 1 | UP |
| NOD27 | 10 | 0 | 180 | 4.33E-07 | 31 | 4.94E-05 | 1 | 192 | 6.60E-07 | 8.56E-04 | 1 | UP |
| RAD54L2 | 10 | 0 | 181 | 4.33E-07 | 96 | 4.94E-05 | 1 | 192 | 6.60E-07 | 8.56E-04 | 1 | UP |
| RAPGEF3 | 10 | 0 | 182 | 4.33E-07 | 121 | 4.94E-05 | 1 | 192 | 6.60E-07 | 8.56E-04 | 1 | UP |
| MAT2A | 22 | 13 | 183 | 5.32E-07 | 247 | 4.94E-05 | 0.99 | 194 | 7.07E-07 | 8.76E-04 | 1 | UP |
| RPS13 | 39 | 46 | 184 | 6.03E-07 | 44 | 4.94E-05 | 0.91 | 212 | 3.21E-06 | 9.91E-04 | 1 | UP |
| FAM43A | 13 | 2 | 185 | 6.56E-07 | 61 | 4.94E-05 | 1 | 188 | 4.93E-07 | 8.27E-04 | 1 | UP |
| ICAM2 | 13 | 2 | 186 | 6.56E-07 | 146 | 4.94E-05 | 1 | 188 | 4.93E-07 | 8.27E-04 | 1 | UP |
| COL1A1 | 1 | 99 | 187 | 6.69E-07 | 7 | 4.94E-05 | 1 | 125 | 5.22E-10 | 5.32E-04 | 1 | down |
| PRSS23 | 15 | 4 | 188 | 7.22E-07 | 180 | 4.94E-05 | 1 | 190 | 5.71E-07 | 8.43E-04 | 1 | UP |
| RALA | 15 | 4 | 189 | 7.22E-07 | 238 | 4.94E-05 | 1 | 190 | 5.71E-07 | 8.43E-04 | 1 | UP |
| LOC653352 | 44 | 58 | 190 | 7.34E-07 | 278 | 8.63E-05 | 0.85 | 218 | 5.66E-06 | 1.02E-03 | 1 | UP |
| RNF4 | 14 | 3 | 191 | 7.35E-07 | 8 | 4.94E-05 | 1 | 189 | 5.66E-07 | 8.35E-04 | 1 | UP |
| GPR56 | 14 | 3 | 192 | 7.35E-07 | 153 | 4.94E-05 | 1 | 189 | 5.66E-07 | 8.35E-04 | 1 | UP |
| LAMA4 | 19 | 9 | 193 | 7.36E-07 | 127 | 4.94E-05 | 0.99 | 196 | 7.75E-07 | 8.84E-04 | 1 | UP |
| TXNRD1 | 24 | 17 | 194 | 8.73E-07 | 27 | 4.94E-05 | 0.99 | 200 | 1.38E-06 | 9.01E-04 | 1 | UP |
| PRDX3 | 1 | 97 | 195 | 9.59E-07 | 51 | 4.94E-05 | 1 | 128 | 8.52E-10 | 5.45E-04 | 1 | down |
| NM_001042465 | 36 | 41 | 196 | 1.12E-06 | 102 | 4.94E-05 | 0.92 | 216 | 4.61E-06 | 1.01E-03 | 1 | UP |
| KRT8 | 0 | 77 | 197 | 1.18E-06 | 79 | 4.94E-05 | 1 | 143 | 6.09E-09 | 6.06E-04 | 1 | down |
| NUCB1 | 23 | 16 | 198 | 1.39E-06 | 137 | 4.94E-05 | 0.99 | 206 | 1.96E-06 | 9.33E-04 | 1 | UP |
| SPP1 | 1 | 95 | 199 | 1.40E-06 | 80 | 4.94E-05 | 1 | 132 | 1.39E-09 | 5.61E-04 | 1 | down |
| KRT17 | 0 | 76 | 200 | 1.43E-06 | 194 | 4.94E-05 | 1 | 146 | 7.86E-09 | 6.22E-04 | 1 | down |
| RPL10A | 34 | 362 | 201 | 1.44E-06 | 160 | 4.94E-05 | 0.99 | 79 | 4.08E-13 | 3.23E-04 | 1 | down |
| CS | 24 | 18 | 202 | 1.65E-06 | 42 | 4.94E-05 | 0.98 | 210 | 2.53E-06 | 9.54E-04 | 1 | UP |
| CDC42 | 19 | 10 | 203 | 1.76E-06 | 169 | 4.94E-05 | 0.99 | 204 | 1.77E-06 | 9.25E-04 | 1 | UP |
| ART4 | 11 | 1 | 204 | 1.89E-06 | 82 | 4.94E-05 | 0.99 | 201 | 1.41E-06 | 9.05E-04 | 1 | UP |
| PLD2 | 11 | 1 | 205 | 1.89E-06 | 87 | 4.94E-05 | 0.99 | 201 | 1.41E-06 | 9.05E-04 | 1 | UP |
| KIAA1539 | 11 | 1 | 206 | 1.89E-06 | 109 | 4.94E-05 | 0.99 | 201 | 1.41E-06 | 9.05E-04 | 1 | UP |
| NR_002802 | 0 | 74 | 207 | 2.09E-06 | 263 | 8.63E-05 | 1 | 151 | 1.31E-08 | 6.43E-04 | 1 | down |
| NOSTRIN | 9 | 0 | 208 | 2.09E-06 | 6 | 4.94E-05 | 0.99 | 211 | 2.94E-06 | 9.58E-04 | 1 | UP |
| SRPX | 9 | 0 | 209 | 2.09E-06 | 30 | 4.94E-05 | 0.99 | 211 | 2.94E-06 | 9.58E-04 | 1 | UP |
| FZD4 | 9 | 0 | 210 | 2.09E-06 | 37 | 4.94E-05 | 0.99 | 211 | 2.94E-06 | 9.58E-04 | 1 | UP |
| CD34 | 9 | 0 | 211 | 2.09E-06 | 110 | 4.94E-05 | 0.99 | 211 | 2.94E-06 | 9.58E-04 | 1 | UP |
| CALCRL | 9 | 0 | 212 | 2.09E-06 | 120 | 4.94E-05 | 0.99 | 211 | 2.94E-06 | 9.58E-04 | 1 | UP |
| SMURF2 | 9 | 0 | 213 | 2.09E-06 | 148 | 4.94E-05 | 0.99 | 211 | 2.94E-06 | 9.58E-04 | 1 | UP |
| ECSM2 | 9 | 0 | 214 | 2.08E-06 | 173 | 4.94E-05 | 0.99 | 211 | 2.94E-06 | 9.58E-04 | 1 | UP |
| SLC35A2 | 9 | 0 | 214 | 2.09E-06 | 206 | 4.94E-05 | 0.99 | 211 | 2.94E-06 | 9.58E-04 | 1 | UP |
| MAP1LC3B | 18 | 9 | 215 | 2.49E-06 | 78 | 4.94E-05 | 0.99 | 209 | 2.36E-06 | 9.50E-04 | 1 | UP |
| GSTM3 | 0 | 73 | 216 | 2.50E-06 | 62 | 4.94E-05 | 1 | 153 | 1.69E-08 | 6.51E-04 | 1 | down |
| ALDOA | 4 | 127 | 217 | 2.69E-06 | 108 | 4.94E-05 | 1 | 126 | 6.01E-10 | 5.36E-04 | 1 | down |
| TNFRSF1A | 12 | 2 | 218 | 2.72E-06 | 140 | 4.94E-05 | 0.99 | 205 | 1.91E-06 | 9.29E-04 | 1 | UP |
| TRAM2 | 13 | 3 | 219 | 2.96E-06 | 138 | 4.94E-05 | 0.99 | 207 | 2.08E-06 | 9.38E-04 | 1 | UP |
| CASP7 | 13 | 3 | 220 | 2.96E-06 | 171 | 4.94E-05 | 0.99 | 207 | 2.08E-06 | 9.38E-04 | 1 | UP |
| RPS7 | 43 | 60 | 221 | 3.03E-06 | 242 | 4.94E-05 | 0.77 | 240 | 2.00E-05 | 1.13E-03 | 1 | UP |
| MMP2 | 19 | 11 | 222 | 3.77E-06 | 1 | 4.94E-05 | 0.99 | 215 | 3.81E-06 | 1.01E-03 | 1 | UP |
| KRT18 | 6 | 144 | 223 | 4.12E-06 | 93 | 4.94E-05 | 1 | 124 | 5.07E-10 | 5.28E-04 | 1 | down |
| MGAT4B | 0 | 70 | 224 | 4.55E-06 | 69 | 4.94E-05 | 1 | 159 | 3.62E-08 | 6.84E-04 | 1 | down |
| MAPRE1 | 21 | 15 | 225 | 6.54E-06 | 191 | 4.94E-05 | 0.98 | 226 | 7.41E-06 | 1.06E-03 | 1 | UP |
| ELOVL5 | 19 | 12 | 226 | 8.22E-06 | 188 | 4.94E-05 | 0.98 | 228 | 7.75E-06 | 1.06E-03 | 1 | UP |
| BRD9 | 0 | 67 | 227 | 8.44E-06 | 112 | 4.94E-05 | 1 | 168 | 7.77E-08 | 7.25E-04 | 1 | down |
| KIAA0101 | 0 | 67 | 228 | 8.44E-06 | 216 | 4.94E-05 | 1 | 168 | 7.77E-08 | 7.25E-04 | 1 | down |
| CLIC4 | 23 | 19 | 229 | 8.59E-06 | 22 | 4.94E-05 | 0.97 | 230 | 1.13E-05 | 1.07E-03 | 1 | UP |
| SRP9 | 5 | 130 | 230 | 8.69E-06 | 233 | 4.94E-05 | 1 | 135 | 1.91E-09 | 5.73E-04 | 1 | down |
| MOV10 | 14 | 5 | 231 | 9.06E-06 | 52 | 4.94E-05 | 0.99 | 220 | 6.06E-06 | 1.03E-03 | 1 | UP |
| GRWD1 | 14 | 5 | 232 | 9.06E-06 | 75 | 4.94E-05 | 0.99 | 220 | 6.06E-06 | 1.03E-03 | 1 | UP |
| SURF4 | 24 | 21 | 233 | 9.13E-06 | 199 | 4.94E-05 | 0.96 | 235 | 1.31E-05 | 1.11E-03 | 1 | UP |
| THBD | 8 | 0 | 234 | 1.06E-05 | 46 | 4.94E-05 | 0.98 | 234 | 1.31E-05 | 1.09E-03 | 1 | UP |
| MUS81 | 8 | 0 | 235 | 1.06E-05 | 144 | 4.94E-05 | 0.98 | 234 | 1.31E-05 | 1.09E-03 | 1 | UP |
| LRRC8C | 8 | 0 | 237 | 1.06E-05 | 220 | 4.94E-05 | 0.98 | 234 | 1.31E-05 | 1.09E-03 | 1 | UP |
| CCL20 | 8 | 0 | 238 | 1.06E-05 | 227 | 4.94E-05 | 0.98 | 234 | 1.31E-05 | 1.09E-03 | 1 | UP |
| LIFR | 8 | 0 | 239 | 1.06E-05 | 237 | 4.94E-05 | 0.98 | 234 | 1.31E-05 | 1.09E-03 | 1 | UP |
| UBE3C | 13 | 4 | 240 | 1.09E-05 | 282 | 8.63E-05 | 0.99 | 224 | 7.01E-06 | 1.05E-03 | 1 | UP |
| SRPX2 | 11 | 2 | 241 | 1.19E-05 | 88 | 4.94E-05 | 0.99 | 225 | 7.30E-06 | 1.05E-03 | 1 | UP |
| C3 | 0 | 64 | 242 | 1.50E-05 | 251 | 7.02E-05 | 1 | 178 | 1.67E-07 | 7.82E-04 | 1 | down |
| ALAS2 | 0 | 64 | 243 | 1.50E-05 | 256 | 7.02E-05 | 1 | 178 | 1.67E-07 | 7.82E-04 | 1 | down |
| COL1A2 | 1 | 81 | 244 | 1.89E-05 | 156 | 4.94E-05 | 1 | 161 | 4.22E-08 | 6.92E-04 | 1 | down |
| RPS15 | 33 | 42 | 245 | 2.16E-05 | 274 | 8.63E-05 | 0.82 | 260 | 5.78E-05 | 1.24E-03 | 1 | UP |
| RPL24 | 32 | 40 | 246 | 2.42E-05 | 271 | 8.63E-05 | 0.83 | 263 | 5.94E-05 | 1.30E-03 | 1 | UP |
| EEF1G | 50 | 436 | 247 | 2.43E-05 | 268 | 8.63E-05 | 0.93 | 89 | 3.98E-12 | 3.64E-04 | 1 | down |
| HEXB | 14 | 6 | 248 | 2.55E-05 | 266 | 8.63E-05 | 0.98 | 238 | 1.60E-05 | 1.12E-03 | 1 | UP |
| WASF2 | 24 | 23 | 249 | 2.56E-05 | 287 | 0.000105649 | 0.93 | 248 | 3.47E-05 | 1.18E-03 | 1 | UP |
| UBA52 | 24 | 23 | 250 | 2.56E-05 | 289 | 0.000105649 | 0.93 | 248 | 3.47E-05 | 1.18E-03 | 1 | UP |
| RPL27A | 37 | 52 | 251 | 2.56E-05 | 300 | 0.00012213 | 0.73 | 267 | 8.97E-05 | 1.32E-03 | 1 | UP |
| TPI1 | 8 | 149 | 252 | 2.95E-05 | 70 | 4.94E-05 | 1 | 141 | 4.02E-09 | 5.98E-04 | 1 | down |
| SHC1 | 20 | 16 | 253 | 3.58E-05 | 276 | 8.63E-05 | 0.95 | 247 | 3.45E-05 | 1.17E-03 | 1 | UP |
| TP53 | 20 | 16 | 254 | 3.58E-05 | 277 | 8.63E-05 | 0.95 | 247 | 3.45E-05 | 1.17E-03 | 1 | UP |
| PALMD | 9 | 1 | 255 | 4.05E-05 | 25 | 4.94E-05 | 0.98 | 241 | 2.34E-05 | 1.13E-03 | 1 | UP |
| PCDH1 | 9 | 1 | 256 | 4.05E-05 | 54 | 4.94E-05 | 0.98 | 241 | 2.34E-05 | 1.13E-03 | 1 | UP |
| RANBP1 | 9 | 1 | 257 | 4.05E-05 | 197 | 4.94E-05 | 0.98 | 241 | 2.34E-05 | 1.13E-03 | 1 | UP |
| PGAM1 | 0 | 59 | 258 | 4.09E-05 | 77 | 4.94E-05 | 0.99 | 191 | 5.96E-07 | 8.52E-04 | 1 | down |
| LDLR | 15 | 8 | 259 | 4.30E-05 | 13 | 4.94E-05 | 0.97 | 246 | 2.82E-05 | 1.17E-03 | 1 | UP |
| CNP | 12 | 4 | 260 | 4.34E-05 | 244 | 4.94E-05 | 0.98 | 242 | 2.40E-05 | 1.15E-03 | 1 | UP |
| ABI1 | 18 | 13 | 261 | 4.95E-05 | 116 | 4.94E-05 | 0.96 | 252 | 4.02E-05 | 1.20E-03 | 1 | UP |
| TSC22D2 | 0 | 58 | 262 | 4.98E-05 | 272 | 8.63E-05 | 0.99 | 195 | 7.69E-07 | 8.80E-04 | 1 | down |
| SCAMP4 | 11 | 3 | 263 | 5.08E-05 | 255 | 7.02E-05 | 0.98 | 243 | 2.71E-05 | 1.15E-03 | 1 | UP |
| PMM2 | 11 | 3 | 264 | 5.08E-05 | 261 | 7.02E-05 | 0.98 | 243 | 2.71E-05 | 1.15E-03 | 1 | UP |
| CSTA | 10 | 2 | 265 | 5.16E-05 | 270 | 8.63E-05 | 0.98 | 245 | 2.76E-05 | 1.16E-03 | 1 | UP |
| DNAJC10 | 10 | 2 | 266 | 5.16E-05 | 275 | 8.63E-05 | 0.98 | 245 | 2.76E-05 | 1.16E-03 | 1 | UP |
| TNNC2 | 7 | 0 | 267 | 5.29E-05 | 248 | 7.02E-05 | 0.96 | 261 | 5.81E-05 | 1.24E-03 | 1 | UP |
| TNFSF18 | 7 | 0 | 268 | 5.29E-05 | 249 | 7.02E-05 | 0.96 | 261 | 5.81E-05 | 1.24E-03 | 1 | UP |
| C7 | 7 | 0 | 269 | 5.29E-05 | 250 | 7.02E-05 | 0.96 | 261 | 5.81E-05 | 1.24E-03 | 1 | UP |
| FLJ39531 | 7 | 0 | 270 | 5.29E-05 | 252 | 7.02E-05 | 0.96 | 261 | 5.81E-05 | 1.24E-03 | 1 | UP |
| ECSM1 | 7 | 0 | 271 | 5.29E-05 | 253 | 7.02E-05 | 0.96 | 261 | 5.81E-05 | 1.24E-03 | 1 | UP |
| ATP5H | 7 | 0 | 272 | 5.29E-05 | 254 | 7.02E-05 | 0.96 | 261 | 5.81E-05 | 1.24E-03 | 1 | UP |
| ACVRL1 | 7 | 0 | 273 | 5.29E-05 | 257 | 7.02E-05 | 0.96 | 261 | 5.81E-05 | 1.24E-03 | 1 | UP |
| UACA | 7 | 0 | 274 | 5.29E-05 | 258 | 7.02E-05 | 0.96 | 261 | 5.81E-05 | 1.24E-03 | 1 | UP |
| LOC650049 | 7 | 0 | 275 | 5.29E-05 | 259 | 7.02E-05 | 0.96 | 261 | 5.81E-05 | 1.24E-03 | 1 | UP |
| VEPH1 | 7 | 0 | 276 | 5.29E-05 | 260 | 7.02E-05 | 0.96 | 261 | 5.81E-05 | 1.24E-03 | 1 | UP |
| CD79A | 0 | 57 | 277 | 5.83E-05 | 317 | 0.000192634 | 0.99 | 199 | 9.92E-07 | 8.97E-04 | 1 | down |
| NQO1 | 16 | 10 | 278 | 5.88E-05 | 267 | 8.63E-05 | 0.97 | 253 | 4.25E-05 | 1.21E-03 | 1 | UP |
| TCL1A | 0 | 55 | 279 | 8.86E-05 | 319 | 0.000229712 | 0.99 | 203 | 1.65E-06 | 9.21E-04 | 1 | down |
| LENG4 | 13 | 6 | 280 | 8.86E-05 | 281 | 8.63E-05 | 0.97 | 258 | 5.02E-05 | 1.23E-03 | 1 | UP |
| RPS2 | 63 | 497 | 281 | 0.0001012 | 280 | 8.63E-05 | 0.81 | 94 | 1.02E-11 | 3.93E-04 | 1 | down |
| SNRPB | 2 | 84 | 282 | 0.0001058 | 305 | 0.00015708 | 0.99 | 182 | 2.05E-07 | 8.02E-04 | 1 | down |
| MGC27165 | 0 | 54 | 283 | 0.0001079 | 308 | 0.00015708 | 0.99 | 208 | 2.13E-06 | 9.46E-04 | 1 | down |
| FSTL1 | 21 | 20 | 284 | 0.0001079 | 301 | 0.000141541 | 0.91 | 275 | 1.09E-04 | 1.40E-03 | 1 | UP |
| SERP1 | 21 | 20 | 285 | 0.0001079 | 302 | 0.000141541 | 0.91 | 275 | 1.09E-04 | 1.40E-03 | 1 | UP |
| PTTG1IP | 37 | 57 | 286 | 0.0001142 | 318 | 0.000229712 | 0.59 | 304 | 3.50E-04 | 1.59E-03 | 1 | UP |
| TAGLN2 | 3 | 94 | 287 | 0.0001211 | 307 | 0.00015708 | 0.99 | 174 | 1.50E-07 | 7.61E-04 | 1 | down |
| WSB1 | 12 | 5 | 288 | 0.0001221 | 262 | 8.63E-05 | 0.97 | 264 | 6.48E-05 | 1.30E-03 | 1 | UP |
| NDUFS1 | 12 | 5 | 289 | 0.0001221 | 269 | 8.63E-05 | 0.97 | 264 | 6.48E-05 | 1.30E-03 | 1 | UP |
| TF | 0 | 52 | 290 | 0.0001616 | 310 | 0.00015708 | 0.99 | 214 | 3.55E-06 | 9.99E-04 | 1 | down |
| CLDN1 | 0 | 52 | 291 | 0.0001616 | 311 | 0.00015708 | 0.99 | 214 | 3.55E-06 | 9.99E-04 | 1 | down |
| TARDBP | 19 | 17 | 292 | 0.0001724 | 312 | 0.000174471 | 0.92 | 281 | 1.44E-04 | 1.43E-03 | 1 | UP |
| EFTUD2 | 19 | 17 | 293 | 0.0001724 | 313 | 0.000174471 | 0.92 | 281 | 1.44E-04 | 1.43E-03 | 1 | UP |
| POLR2L | 8 | 1 | 294 | 0.0001799 | 264 | 8.63E-05 | 0.96 | 268 | 9.40E-05 | 1.33E-03 | 1 | UP |
| PLA1A | 8 | 1 | 295 | 0.0001799 | 265 | 8.63E-05 | 0.96 | 268 | 9.40E-05 | 1.33E-03 | 1 | UP |
| FES | 8 | 1 | 296 | 0.0001799 | 273 | 8.63E-05 | 0.96 | 268 | 9.40E-05 | 1.33E-03 | 1 | UP |
| MEF2A | 8 | 1 | 297 | 0.0001799 | 279 | 8.63E-05 | 0.96 | 268 | 9.40E-05 | 1.33E-03 | 1 | UP |
| EXOC6 | 8 | 1 | 298 | 0.0001799 | 283 | 8.63E-05 | 0.96 | 268 | 9.40E-05 | 1.33E-03 | 1 | UP |
| CNN2 | 26 | 32 | 299 | 0.0001922 | 325 | 0.000375785 | 0.8 | 298 | 2.70E-04 | 1.56E-03 | 1 | UP |
| EEF1A1 | 215 | 1305 | 300 | 0.0001964 | 324 | 0.000301556 | 0.04 | 63 | 3.14E-16 | 2.54E-04 | 1 | down |
| EHD2 | 10 | 3 | 301 | 0.0001964 | 284 | 0.000105649 | 0.96 | 269 | 9.52E-05 | 1.35E-03 | 1 | UP |
| NUMB | 10 | 3 | 302 | 0.0001964 | 285 | 0.000105649 | 0.96 | 269 | 9.52E-05 | 1.35E-03 | 1 | UP |
| UBAP2 | 10 | 3 | 303 | 0.0001964 | 286 | 0.000105649 | 0.96 | 269 | 9.52E-05 | 1.35E-03 | 1 | UP |
| SPG20 | 10 | 3 | 304 | 0.0001964 | 288 | 0.000105649 | 0.96 | 269 | 9.52E-05 | 1.35E-03 | 1 | UP |
| LOC653949 | 13 | 7 | 305 | 0.0002065 | 320 | 0.000264678 | 0.96 | 276 | 1.14E-04 | 1.41E-03 | 1 | UP |
| PPM1F | 9 | 2 | 306 | 0.0002107 | 303 | 0.00015708 | 0.96 | 273 | 1.03E-04 | 1.38E-03 | 1 | UP |
| WWP2 | 9 | 2 | 307 | 0.0002107 | 304 | 0.00015708 | 0.96 | 273 | 1.03E-04 | 1.38E-03 | 1 | UP |
| RALB | 9 | 2 | 308 | 0.0002107 | 306 | 0.00015708 | 0.96 | 273 | 1.03E-04 | 1.38E-03 | 1 | UP |
| PPWD1 | 9 | 2 | 309 | 0.0002107 | 309 | 0.00015708 | 0.96 | 273 | 1.03E-04 | 1.38E-03 | 1 | UP |
| PCBP4 | 6 | 0 | 310 | 0.0002647 | 290 | 0.00012213 | 0.93 | 295 | 2.58E-04 | 1.50E-03 | 1 | UP |
| SNX12 | 6 | 0 | 311 | 0.0002647 | 291 | 0.00012213 | 0.93 | 295 | 2.58E-04 | 1.50E-03 | 1 | UP |
| ACTA1 | 6 | 0 | 312 | 0.0002647 | 292 | 0.00012213 | 0.93 | 295 | 2.58E-04 | 1.50E-03 | 1 | UP |
| ACP2 | 6 | 0 | 313 | 0.0002647 | 293 | 0.00012213 | 0.93 | 295 | 2.58E-04 | 1.50E-03 | 1 | UP |
| MOV10L1 | 6 | 0 | 314 | 0.0002647 | 294 | 0.00012213 | 0.93 | 295 | 2.58E-04 | 1.50E-03 | 1 | UP |
| APLN | 6 | 0 | 315 | 0.0002647 | 295 | 0.00012213 | 0.93 | 295 | 2.58E-04 | 1.50E-03 | 1 | UP |
| CCDC99 | 6 | 0 | 316 | 0.0002647 | 296 | 0.00012213 | 0.93 | 295 | 2.58E-04 | 1.50E-03 | 1 | UP |
| LOC152485 | 6 | 0 | 317 | 0.0002647 | 297 | 0.00012213 | 0.93 | 295 | 2.58E-04 | 1.50E-03 | 1 | UP |
| TAF15 | 6 | 0 | 318 | 0.0002647 | 298 | 0.00012213 | 0.93 | 295 | 2.58E-04 | 1.50E-03 | 1 | UP |
| ZNF521 | 6 | 0 | 319 | 0.0002647 | 299 | 0.00012213 | 0.93 | 295 | 2.58E-04 | 1.50E-03 | 1 | UP |
| KRT5 | 0 | 49 | 320 | 0.0002795 | 329 | 0.0004071 | 0.98 | 227 | 7.61E-06 | 1.06E-03 | 1 | down |
| RPL13A | 87 | 210 | 321 | 0.0002807 | 321 | 0.000264678 | 0 | 414 | 7.59E-03 | 2.93E-03 | 0 | UP |
| NDUFB5 | 1 | 66 | 322 | 0.000285 | 328 | 0.0004071 | 0.99 | 202 | 1.59E-06 | 9.17E-04 | 1 | down |
| SEPT7 | 25 | 31 | 323 | 0.0002976 | 362 | 0.000641014 | 0.79 | 310 | 3.91E-04 | 1.66E-03 | 1 | UP |
| OGDH | 15 | 11 | 324 | 0.0003532 | 314 | 0.000174471 | 0.93 | 293 | 2.20E-04 | 1.49E-03 | 1 | UP |
| PTPRF | 15 | 11 | 325 | 0.0003532 | 315 | 0.000174471 | 0.93 | 293 | 2.20E-04 | 1.49E-03 | 1 | UP |
| CD37 | 0 | 47 | 326 | 0.0004214 | 377 | 0.000807738 | 0.98 | 232 | 1.27E-05 | 1.08E-03 | 1 | down |
| RPS27 | 28 | 39 | 327 | 0.0004214 | 330 | 0.0004071 | 0.68 | 322 | 6.51E-04 | 1.77E-03 | 1 | UP |
| LRRC41 | 11 | 5 | 328 | 0.000429 | 316 | 0.000192634 | 0.95 | 291 | 2.05E-04 | 1.48E-03 | 1 | UP |
| CD55 | 18 | 17 | 329 | 0.0004522 | 346 | 0.0004485 | 0.88 | 302 | 3.44E-04 | 1.58E-03 | 1 | UP |
| IGFBP4 | 10 | 145 | 330 | 0.0004743 | 358 | 0.000545832 | 0.98 | 171 | 1.29E-07 | 7.41E-04 | 1 | down |
| LOC647087 | 0 | 46 | 331 | 0.0005147 | 371 | 0.000691302 | 0.98 | 239 | 1.63E-05 | 1.13E-03 | 1 | down |
| AP2B1 | 22 | 26 | 332 | 0.0005943 | 382 | 0.000863223 | 0.8 | 320 | 5.88E-04 | 1.74E-03 | 1 | UP |
| KIAA0195 | 10 | 4 | 333 | 0.0005943 | 322 | 0.000264678 | 0.94 | 296 | 2.66E-04 | 1.54E-03 | 1 | UP |
| SNRK | 10 | 4 | 334 | 0.0005943 | 323 | 0.000264678 | 0.94 | 296 | 2.66E-04 | 1.54E-03 | 1 | UP |
| MGAT1 | 12 | 7 | 335 | 0.0006867 | 326 | 0.000392161 | 0.93 | 301 | 3.33E-04 | 1.58E-03 | 1 | UP |
| TUBB6 | 12 | 7 | 336 | 0.0006867 | 327 | 0.000392161 | 0.93 | 301 | 3.33E-04 | 1.58E-03 | 1 | UP |
| RPLP1 | 37 | 64 | 337 | 0.0007099 | 369 | 0.000676666 | 0.38 | 354 | 1.78E-03 | 2.13E-03 | 1 | UP |
| LGALS1 | 37 | 64 | 338 | 0.0007099 | 370 | 0.000676666 | 0.38 | 354 | 1.78E-03 | 2.13E-03 | 1 | UP |
| HDAC6 | 9 | 3 | 339 | 0.0007663 | 349 | 0.0004485 | 0.94 | 300 | 3.28E-04 | 1.56E-03 | 1 | UP |
| PODXL | 9 | 3 | 340 | 0.0007663 | 350 | 0.0004485 | 0.94 | 300 | 3.28E-04 | 1.56E-03 | 1 | UP |
| KCTD15 | 9 | 3 | 341 | 0.0007663 | 353 | 0.0004485 | 0.94 | 300 | 3.28E-04 | 1.56E-03 | 1 | UP |
| GLTSCR2 | 0 | 44 | 342 | 0.0007663 | 389 | 0.0012213 | 0.97 | 244 | 2.72E-05 | 1.16E-03 | 1 | down |
| SLC7A7 | 7 | 1 | 343 | 0.0007883 | 345 | 0.0004485 | 0.93 | 306 | 3.73E-04 | 1.60E-03 | 1 | UP |
| TCF8 | 7 | 1 | 344 | 0.0007883 | 347 | 0.0004485 | 0.93 | 306 | 3.73E-04 | 1.60E-03 | 1 | UP |
| DRCTNNB1A | 7 | 1 | 345 | 0.0007883 | 348 | 0.0004485 | 0.93 | 306 | 3.73E-04 | 1.60E-03 | 1 | UP |
| FLJ10815 | 7 | 1 | 346 | 0.0007883 | 351 | 0.0004485 | 0.93 | 306 | 3.73E-04 | 1.60E-03 | 1 | UP |
| SPHK1 | 7 | 1 | 347 | 0.0007883 | 352 | 0.0004485 | 0.93 | 306 | 3.73E-04 | 1.60E-03 | 1 | UP |
| INTS3 | 7 | 1 | 348 | 0.0007883 | 354 | 0.0004485 | 0.93 | 306 | 3.73E-04 | 1.60E-03 | 1 | UP |
| PRDX6 | 2 | 72 | 349 | 0.000817 | 383 | 0.001020407 | 0.99 | 213 | 3.26E-06 | 9.95E-04 | 1 | down |
| LCP1 | 1 | 60 | 350 | 0.0008608 | 379 | 0.000837831 | 0.98 | 223 | 6.70E-06 | 1.04E-03 | 1 | down |
| RPLP2 | 25 | 34 | 351 | 0.0008608 | 388 | 0.001196119 | 0.69 | 335 | 1.04E-03 | 1.88E-03 | 1 | UP |
| PIGT | 8 | 2 | 352 | 0.0008608 | 359 | 0.000608958 | 0.94 | 309 | 3.77E-04 | 1.65E-03 | 1 | UP |
| KIAA0690 | 8 | 2 | 353 | 0.0008608 | 360 | 0.000608958 | 0.94 | 309 | 3.77E-04 | 1.65E-03 | 1 | UP |
| PAPSS1 | 8 | 2 | 354 | 0.0008608 | 361 | 0.000608958 | 0.94 | 309 | 3.77E-04 | 1.65E-03 | 1 | UP |
| SMS | 0 | 43 | 355 | 0.0009159 | 387 | 0.001167651 | 0.97 | 249 | 3.51E-05 | 1.19E-03 | 1 | down |
| MED28 | 11 | 6 | 356 | 0.001011 | 372 | 0.000755105 | 0.92 | 314 | 4.63E-04 | 1.69E-03 | 1 | UP |
| HLA-DPB1 | 0 | 42 | 357 | 0.0011302 | 402 | 0.00151903 | 0.97 | 254 | 4.53E-05 | 1.21E-03 | 1 | down |
| TRIM28 | 2 | 70 | 358 | 0.0011567 | 397 | 0.001261292 | 0.98 | 217 | 5.15E-06 | 1.02E-03 | 1 | down |
| BRD2 | 16 | 15 | 359 | 0.0011848 | 375 | 0.000807738 | 0.86 | 327 | 7.46E-04 | 1.81E-03 | 1 | UP |
| RPL36 | 16 | 15 | 360 | 0.0011848 | 376 | 0.000807738 | 0.86 | 327 | 7.46E-04 | 1.81E-03 | 1 | UP |
| NFE2L1 | 16 | 15 | 361 | 0.0011848 | 378 | 0.000807738 | 0.86 | 327 | 7.46E-04 | 1.81E-03 | 1 | UP |
| RPL41 | 27 | 40 | 362 | 0.0012308 | 401 | 0.001400993 | 0.6 | 352 | 1.63E-03 | 2.12E-03 | 1 | UP |
| ITGB1 | 35 | 61 | 363 | 0.001252 | 398 | 0.001319495 | 0.37 | 370 | 2.66E-03 | 2.24E-03 | 0 | UP |
| RPS14 | 32 | 53 | 364 | 0.0012684 | 396 | 0.001233636 | 0.45 | 362 | 2.27E-03 | 2.20E-03 | 0 | UP |
| MAP3K8 | 5 | 0 | 365 | 0.0013089 | 331 | 0.000426035 | 0.87 | 337 | 1.15E-03 | 1.91E-03 | 1 | UP |
| JAM2 | 5 | 0 | 366 | 0.0013089 | 332 | 0.000426035 | 0.87 | 337 | 1.15E-03 | 1.91E-03 | 1 | UP |
| SLC39A9 | 5 | 0 | 367 | 0.0013089 | 333 | 0.000426035 | 0.87 | 337 | 1.15E-03 | 1.91E-03 | 1 | UP |
| C16orf30 | 5 | 0 | 368 | 0.0013089 | 334 | 0.000426035 | 0.87 | 337 | 1.15E-03 | 1.91E-03 | 1 | UP |
| LOC650626 | 5 | 0 | 369 | 0.0013089 | 335 | 0.000426035 | 0.87 | 337 | 1.15E-03 | 1.91E-03 | 1 | UP |
| SPINK5 | 5 | 0 | 370 | 0.0013089 | 336 | 0.000426035 | 0.87 | 337 | 1.15E-03 | 1.91E-03 | 1 | UP |
| GBP4 | 5 | 0 | 371 | 0.0013089 | 337 | 0.000426035 | 0.87 | 337 | 1.15E-03 | 1.91E-03 | 1 | UP |
| ANTXR2 | 5 | 0 | 372 | 0.0013089 | 338 | 0.000426035 | 0.87 | 337 | 1.15E-03 | 1.91E-03 | 1 | UP |
| IL1RL1 | 5 | 0 | 373 | 0.0013089 | 339 | 0.000426035 | 0.87 | 337 | 1.15E-03 | 1.91E-03 | 1 | UP |
| TMEM154 | 5 | 0 | 374 | 0.0013089 | 340 | 0.000426035 | 0.87 | 337 | 1.15E-03 | 1.91E-03 | 1 | UP |
| BMP6 | 5 | 0 | 375 | 0.0013089 | 341 | 0.000426035 | 0.87 | 337 | 1.15E-03 | 1.91E-03 | 1 | UP |
| RNASE1 | 5 | 0 | 376 | 0.0013089 | 342 | 0.000426035 | 0.87 | 337 | 1.15E-03 | 1.91E-03 | 1 | UP |
| NUP54 | 5 | 0 | 377 | 0.0013089 | 343 | 0.000426035 | 0.87 | 337 | 1.15E-03 | 1.91E-03 | 1 | UP |
| RHOBTB3 | 5 | 0 | 378 | 0.0013089 | 344 | 0.000426035 | 0.87 | 337 | 1.15E-03 | 1.91E-03 | 1 | UP |
| MORF4L1 | 0 | 41 | 379 | 0.0013145 | 453 | 0.001798398 | 0.96 | 262 | 5.84E-05 | 1.29E-03 | 1 | down |
| TAGLN | 0 | 41 | 380 | 0.0013145 | 454 | 0.001798398 | 0.96 | 262 | 5.84E-05 | 1.29E-03 | 1 | down |
| SEPT15 | 0 | 41 | 381 | 0.0013145 | 455 | 0.001798398 | 0.96 | 262 | 5.84E-05 | 1.29E-03 | 1 | down |
| KIAA0652 | 12 | 8 | 382 | 0.0013238 | 355 | 0.000513151 | 0.9 | 323 | 6.62E-04 | 1.78E-03 | 1 | UP |
| GSN | 12 | 8 | 383 | 0.0013238 | 356 | 0.000513151 | 0.9 | 323 | 6.62E-04 | 1.78E-03 | 1 | UP |
| WDR46 | 12 | 8 | 384 | 0.0013238 | 357 | 0.000513151 | 0.9 | 323 | 6.62E-04 | 1.78E-03 | 1 | UP |
| DPM1 | 1 | 57 | 385 | 0.0014033 | 405 | 0.001568089 | 0.98 | 236 | 1.37E-05 | 1.11E-03 | 1 | down |
| UBN1 | 10 | 5 | 386 | 0.0014033 | 363 | 0.00066375 | 0.92 | 321 | 6.35E-04 | 1.75E-03 | 1 | UP |
| LDB2 | 10 | 5 | 387 | 0.0014033 | 364 | 0.00066375 | 0.92 | 321 | 6.35E-04 | 1.75E-03 | 1 | UP |
| IFI16 | 10 | 5 | 388 | 0.0014033 | 365 | 0.00066375 | 0.92 | 321 | 6.35E-04 | 1.75E-03 | 1 | UP |
| ECOP | 10 | 5 | 389 | 0.0014033 | 366 | 0.00066375 | 0.92 | 321 | 6.35E-04 | 1.75E-03 | 1 | UP |
| MOAP1 | 10 | 5 | 390 | 0.0014033 | 367 | 0.00066375 | 0.92 | 321 | 6.35E-04 | 1.75E-03 | 1 | UP |
| NUPL1 | 10 | 5 | 391 | 0.0014033 | 368 | 0.00066375 | 0.92 | 321 | 6.35E-04 | 1.75E-03 | 1 | UP |
| P4HB | 31 | 51 | 392 | 0.0014133 | 419 | 0.001632286 | 0.46 | 367 | 2.48E-03 | 2.22E-03 | 0 | UP |
| PGD | 13 | 10 | 393 | 0.0015701 | 373 | 0.000767394 | 0.89 | 328 | 8.40E-04 | 1.83E-03 | 1 | UP |
| SFRS1 | 13 | 10 | 394 | 0.0015701 | 374 | 0.000767394 | 0.89 | 328 | 8.40E-04 | 1.83E-03 | 1 | UP |
| NEFL | 0 | 40 | 395 | 0.0015701 | 458 | 0.002088716 | 0.96 | 265 | 7.53E-05 | 1.31E-03 | 1 | down |
| LOC649143 | 0 | 40 | 396 | 0.0015701 | 459 | 0.002088716 | 0.96 | 265 | 7.53E-05 | 1.31E-03 | 1 | down |
| RPS25 | 33 | 57 | 397 | 0.0016652 | 450 | 0.00165554 | 0.38 | 376 | 3.22E-03 | 2.31E-03 | 0 | UP |
| AZIN1 | 18 | 20 | 398 | 0.0016652 | 467 | 0.002213443 | 0.79 | 342 | 1.26E-03 | 1.99E-03 | 1 | UP |
| PRKCSH | 18 | 20 | 399 | 0.0016652 | 468 | 0.002213443 | 0.79 | 342 | 1.26E-03 | 1.99E-03 | 1 | UP |
| RPL30 | 18 | 20 | 400 | 0.0016652 | 469 | 0.002213443 | 0.79 | 342 | 1.26E-03 | 1.99E-03 | 1 | UP |
| FTH1 | 114 | 318 | 401 | 0.0019113 | 452 | 0.001783314 | 0 | 543 | 6.26E-02 | 5.31E-03 | 0 | UP |
| PTPRCAP | 0 | 39 | 402 | 0.0019113 | 470 | 0.002509875 | 0.96 | 271 | 9.72E-05 | 1.37E-03 | 1 | down |
| HLA-DPA1 | 0 | 39 | 403 | 0.0019113 | 471 | 0.002509875 | 0.96 | 271 | 9.72E-05 | 1.37E-03 | 1 | down |
| FNDC3A | 0 | 39 | 404 | 0.0019113 | 472 | 0.002509875 | 0.96 | 271 | 9.72E-05 | 1.37E-03 | 1 | down |
| HSPD1 | 32 | 55 | 405 | 0.001974 | 451 | 0.001733109 | 0.39 | 378 | 3.54E-03 | 2.33E-03 | 0 | UP |
| GBE1 | 9 | 4 | 406 | 0.0019846 | 380 | 0.000849461 | 0.91 | 329 | 8.53E-04 | 1.83E-03 | 1 | UP |
| PGM2 | 9 | 4 | 407 | 0.0019846 | 381 | 0.000849461 | 0.91 | 329 | 8.53E-04 | 1.83E-03 | 1 | UP |
| PITRM1 | 11 | 7 | 408 | 0.0020107 | 384 | 0.001044117 | 0.9 | 332 | 9.49E-04 | 1.85E-03 | 1 | UP |
| UGDH | 11 | 7 | 409 | 0.0020107 | 385 | 0.001044117 | 0.9 | 332 | 9.49E-04 | 1.85E-03 | 1 | UP |
| CBARA1 | 11 | 7 | 410 | 0.0020107 | 386 | 0.001044117 | 0.9 | 332 | 9.49E-04 | 1.85E-03 | 1 | UP |
| EEF1D | 3 | 76 | 411 | 0.0021085 | 457 | 0.002004322 | 0.98 | 229 | 8.10E-06 | 1.07E-03 | 1 | down |
| CALR | 2 | 66 | 412 | 0.0021139 | 478 | 0.002810523 | 0.98 | 233 | 1.28E-05 | 1.08E-03 | 1 | down |
| RBMS1 | 0 | 38 | 413 | 0.0023225 | 480 | 0.002951475 | 0.95 | 280 | 1.25E-04 | 1.43E-03 | 1 | down |
| RPL37 | 22 | 30 | 414 | 0.0023244 | 492 | 0.003152543 | 0.66 | 361 | 2.25E-03 | 2.19E-03 | 0 | UP |
| G3BP | 12 | 9 | 415 | 0.0024943 | 399 | 0.001328164 | 0.87 | 339 | 1.23E-03 | 1.97E-03 | 1 | UP |
| PLOD3 | 12 | 9 | 416 | 0.0024943 | 400 | 0.001328164 | 0.87 | 339 | 1.23E-03 | 1.97E-03 | 1 | UP |
| TFPI | 8 | 3 | 417 | 0.0026602 | 390 | 0.0012213 | 0.9 | 336 | 1.11E-03 | 1.89E-03 | 1 | UP |
| LOX | 8 | 3 | 418 | 0.0026602 | 391 | 0.0012213 | 0.9 | 336 | 1.11E-03 | 1.89E-03 | 1 | UP |
| PCSK7 | 8 | 3 | 419 | 0.0026602 | 392 | 0.0012213 | 0.9 | 336 | 1.11E-03 | 1.89E-03 | 1 | UP |
| C16orf63 | 8 | 3 | 420 | 0.0026602 | 393 | 0.0012213 | 0.9 | 336 | 1.11E-03 | 1.89E-03 | 1 | UP |
| FAM8A1 | 8 | 3 | 421 | 0.0026602 | 394 | 0.0012213 | 0.9 | 336 | 1.11E-03 | 1.89E-03 | 1 | UP |
| UBL5 | 8 | 3 | 422 | 0.0026602 | 395 | 0.0012213 | 0.9 | 336 | 1.11E-03 | 1.89E-03 | 1 | UP |
| IFI27 | 13 | 11 | 423 | 0.002766 | 403 | 0.00154174 | 0.85 | 346 | 1.46E-03 | 2.03E-03 | 1 | UP |
| DAP | 13 | 11 | 424 | 0.002766 | 404 | 0.00154174 | 0.85 | 346 | 1.46E-03 | 2.03E-03 | 1 | UP |
| PFN1 | 5 | 92 | 425 | 0.002766 | 491 | 0.003084342 | 0.97 | 219 | 6.01E-06 | 1.02E-03 | 1 | down |
| TUFM | 0 | 37 | 426 | 0.0027912 | 493 | 0.003362283 | 0.95 | 285 | 1.62E-04 | 1.45E-03 | 1 | down |
| RNF128 | 0 | 37 | 427 | 0.0027912 | 494 | 0.003362283 | 0.95 | 285 | 1.62E-04 | 1.45E-03 | 1 | down |
| MT2A | 16 | 17 | 428 | 0.0028037 | 495 | 0.003873618 | 0.79 | 356 | 1.82E-03 | 2.14E-03 | 1 | UP |
| ZCD1 | 1 | 53 | 429 | 0.0028142 | 481 | 0.002966014 | 0.97 | 250 | 3.55E-05 | 1.20E-03 | 1 | down |
| SEPN1 | 14 | 13 | 430 | 0.002861 | 447 | 0.00165554 | 0.83 | 351 | 1.63E-03 | 2.11E-03 | 1 | UP |
| GSTO1 | 14 | 13 | 431 | 0.002861 | 448 | 0.00165554 | 0.83 | 351 | 1.63E-03 | 2.11E-03 | 1 | UP |
| XPO6 | 14 | 13 | 432 | 0.002861 | 449 | 0.00165554 | 0.83 | 351 | 1.63E-03 | 2.11E-03 | 1 | UP |
| PPM1G | 20 | 26 | 433 | 0.0029165 | 502 | 0.003916918 | 0.69 | 366 | 2.48E-03 | 2.22E-03 | 0 | UP |
| ARID1A | 10 | 6 | 434 | 0.0030328 | 406 | 0.001621583 | 0.88 | 345 | 1.35E-03 | 2.02E-03 | 1 | UP |
| COPG | 10 | 6 | 435 | 0.0030328 | 407 | 0.001621583 | 0.88 | 345 | 1.35E-03 | 2.02E-03 | 1 | UP |
| GTPBP4 | 10 | 6 | 436 | 0.0030328 | 408 | 0.001621583 | 0.88 | 345 | 1.35E-03 | 2.02E-03 | 1 | UP |
| TMEM43 | 6 | 1 | 437 | 0.0031283 | 409 | 0.001621583 | 0.88 | 347 | 1.46E-03 | 2.04E-03 | 1 | UP |
| DDR2 | 6 | 1 | 438 | 0.0031283 | 410 | 0.001621583 | 0.88 | 347 | 1.46E-03 | 2.04E-03 | 1 | UP |
| SLC35F2 | 6 | 1 | 439 | 0.0031283 | 411 | 0.001621583 | 0.88 | 347 | 1.46E-03 | 2.04E-03 | 1 | UP |
| TTLL5 | 6 | 1 | 440 | 0.0031283 | 412 | 0.001621583 | 0.88 | 347 | 1.46E-03 | 2.04E-03 | 1 | UP |
| LOC646195 | 6 | 1 | 441 | 0.0031283 | 413 | 0.001621583 | 0.88 | 347 | 1.46E-03 | 2.04E-03 | 1 | UP |
| BIVM | 6 | 1 | 442 | 0.0031283 | 414 | 0.001621583 | 0.88 | 347 | 1.46E-03 | 2.04E-03 | 1 | UP |
| STAB1 | 6 | 1 | 443 | 0.0031283 | 415 | 0.001621583 | 0.88 | 347 | 1.46E-03 | 2.04E-03 | 1 | UP |
| OGFOD1 | 6 | 1 | 444 | 0.0031283 | 416 | 0.001621583 | 0.88 | 347 | 1.46E-03 | 2.04E-03 | 1 | UP |
| FNBP1L | 6 | 1 | 445 | 0.0031283 | 417 | 0.001621583 | 0.88 | 347 | 1.46E-03 | 2.04E-03 | 1 | UP |
| POLR3H | 6 | 1 | 446 | 0.0031283 | 418 | 0.001621583 | 0.88 | 347 | 1.46E-03 | 2.04E-03 | 1 | UP |
| PIGG | 7 | 2 | 447 | 0.0031291 | 464 | 0.002201485 | 0.89 | 344 | 1.35E-03 | 2.01E-03 | 1 | UP |
| C12orf11 | 7 | 2 | 448 | 0.0031291 | 465 | 0.002201485 | 0.89 | 344 | 1.35E-03 | 2.01E-03 | 1 | UP |
| ARAF | 7 | 2 | 449 | 0.0031291 | 466 | 0.002201485 | 0.89 | 344 | 1.35E-03 | 2.01E-03 | 1 | UP |
| TPP1 | 11 | 8 | 450 | 0.0038384 | 456 | 0.001901586 | 0.86 | 355 | 1.80E-03 | 2.14E-03 | 1 | UP |
| GABARAPL2 | 0 | 35 | 451 | 0.0040836 | 512 | 0.005176213 | 0.94 | 297 | 2.69E-04 | 1.55E-03 | 1 | down |
| RPS17 | 29 | 251 | 452 | 0.0041894 | 501 | 0.003916918 | 0.83 | 183 | 2.11E-07 | 8.07E-04 | 1 | down |
| RGS5 | 12 | 10 | 453 | 0.0043832 | 446 | 0.001643004 | 0.84 | 360 | 2.15E-03 | 2.19E-03 | 1 | UP |
| MCFD2 | 9 | 5 | 454 | 0.0045648 | 460 | 0.002110238 | 0.87 | 358 | 1.91E-03 | 2.15E-03 | 1 | UP |
| MAP2K3 | 9 | 5 | 455 | 0.0045648 | 461 | 0.002110238 | 0.87 | 358 | 1.91E-03 | 2.15E-03 | 1 | UP |
| DLC1 | 9 | 5 | 456 | 0.0045648 | 462 | 0.002110238 | 0.87 | 358 | 1.91E-03 | 2.15E-03 | 1 | UP |
| LTA4H | 9 | 5 | 457 | 0.0045648 | 463 | 0.002110238 | 0.87 | 358 | 1.91E-03 | 2.15E-03 | 1 | UP |
| EIF2S3 | 13 | 12 | 458 | 0.0045883 | 476 | 0.002739604 | 0.81 | 364 | 2.41E-03 | 2.21E-03 | 0 | UP |
| PSME3 | 13 | 12 | 459 | 0.0045883 | 477 | 0.002739604 | 0.81 | 364 | 2.41E-03 | 2.21E-03 | 0 | UP |
| WBP2 | 19 | 25 | 460 | 0.0045966 | 532 | 0.00602398 | 0.66 | 379 | 3.59E-03 | 2.34E-03 | 0 | UP |
| HIGD1A | 2 | 61 | 461 | 0.0047557 | 511 | 0.004995141 | 0.96 | 251 | 3.95E-05 | 1.20E-03 | 1 | down |
| EWSR1 | 21 | 30 | 462 | 0.0049241 | 510 | 0.004861253 | 0.59 | 388 | 4.34E-03 | 2.42E-03 | 0 | UP |
| AES | 0 | 34 | 463 | 0.0049475 | 534 | 0.00602398 | 0.93 | 303 | 3.47E-04 | 1.59E-03 | 1 | down |
| SAT | 2 | 60 | 464 | 0.0056777 | 515 | 0.005810068 | 0.96 | 257 | 4.95E-05 | 1.23E-03 | 1 | down |
| K-ALPHA-1 | 52 | 381 | 465 | 0.0058218 | 514 | 0.005678807 | 0.6 | 157 | 3.39E-08 | 6.71E-04 | 1 | down |
| CFL1 | 21 | 198 | 466 | 0.0058722 | 513 | 0.005523228 | 0.87 | 198 | 9.80E-07 | 8.92E-04 | 1 | down |
| ANKRD17 | 0 | 33 | 467 | 0.0058844 | 603 | 0.00686101 | 0.92 | 312 | 4.48E-04 | 1.67E-03 | 1 | down |
| MMP12 | 0 | 33 | 468 | 0.0058844 | 604 | 0.00686101 | 0.92 | 312 | 4.48E-04 | 1.67E-03 | 1 | down |
| ERP29 | 0 | 33 | 469 | 0.0058844 | 605 | 0.00686101 | 0.92 | 312 | 4.48E-04 | 1.67E-03 | 1 | down |
| SF3B2 | 0 | 33 | 470 | 0.0058844 | 606 | 0.00686101 | 0.92 | 312 | 4.48E-04 | 1.67E-03 | 1 | down |
| CDH1 | 0 | 33 | 471 | 0.0058844 | 607 | 0.00686101 | 0.92 | 312 | 4.48E-04 | 1.67E-03 | 1 | down |
| PPIL2 | 10 | 7 | 472 | 0.0058844 | 473 | 0.002571158 | 0.84 | 369 | 2.62E-03 | 2.23E-03 | 0 | UP |
| LOC653105 | 10 | 7 | 473 | 0.0058844 | 474 | 0.002571158 | 0.84 | 369 | 2.62E-03 | 2.23E-03 | 0 | UP |
| METAP2 | 10 | 7 | 474 | 0.0058844 | 475 | 0.002571158 | 0.84 | 369 | 2.62E-03 | 2.23E-03 | 0 | UP |
| VCP | 25 | 41 | 475 | 0.0058844 | 537 | 0.00602398 | 0.44 | 408 | 6.82E-03 | 2.85E-03 | 0 | UP |
| TEK | 4 | 0 | 476 | 0.0058844 | 420 | 0.001632974 | 0.78 | 393 | 5.11E-03 | 2.54E-03 | 0 | UP |
| ZNF586 | 4 | 0 | 477 | 0.0058844 | 421 | 0.001632974 | 0.78 | 393 | 5.11E-03 | 2.54E-03 | 0 | UP |
| HEY1 | 4 | 0 | 478 | 0.0058844 | 422 | 0.001632974 | 0.78 | 393 | 5.11E-03 | 2.54E-03 | 0 | UP |
| FBXL3 | 4 | 0 | 479 | 0.0058844 | 423 | 0.001632974 | 0.78 | 393 | 5.11E-03 | 2.54E-03 | 0 | UP |
| MMRN2 | 4 | 0 | 480 | 0.0058844 | 424 | 0.001632974 | 0.78 | 393 | 5.11E-03 | 2.54E-03 | 0 | UP |
| GTF3C4 | 4 | 0 | 481 | 0.0058844 | 425 | 0.001632974 | 0.78 | 393 | 5.11E-03 | 2.54E-03 | 0 | UP |
| FABP4 | 4 | 0 | 482 | 0.0058844 | 426 | 0.001632974 | 0.78 | 393 | 5.11E-03 | 2.54E-03 | 0 | UP |
| SLC35A5 | 4 | 0 | 483 | 0.0058844 | 427 | 0.001632974 | 0.78 | 393 | 5.11E-03 | 2.54E-03 | 0 | UP |
| FNTB | 4 | 0 | 484 | 0.0058844 | 428 | 0.001632974 | 0.78 | 393 | 5.11E-03 | 2.54E-03 | 0 | UP |
| RGS1 | 4 | 0 | 485 | 0.0058844 | 429 | 0.001632974 | 0.78 | 393 | 5.11E-03 | 2.54E-03 | 0 | UP |
| NR_002716 | 4 | 0 | 486 | 0.0058844 | 430 | 0.001632974 | 0.78 | 393 | 5.11E-03 | 2.54E-03 | 0 | UP |
| STARD4 | 4 | 0 | 487 | 0.0058844 | 431 | 0.001632974 | 0.78 | 393 | 5.11E-03 | 2.54E-03 | 0 | UP |
| PI4K2B | 4 | 0 | 488 | 0.0058844 | 432 | 0.001632974 | 0.78 | 393 | 5.11E-03 | 2.54E-03 | 0 | UP |
| XLKD1 | 4 | 0 | 489 | 0.0058844 | 433 | 0.001632974 | 0.78 | 393 | 5.11E-03 | 2.54E-03 | 0 | UP |
| GPR4 | 4 | 0 | 490 | 0.0058844 | 434 | 0.001632974 | 0.78 | 393 | 5.11E-03 | 2.54E-03 | 0 | UP |
| TM6SF1 | 4 | 0 | 491 | 0.0058844 | 435 | 0.001632974 | 0.78 | 393 | 5.11E-03 | 2.54E-03 | 0 | UP |
| SHQ1 | 4 | 0 | 492 | 0.0058844 | 436 | 0.001632974 | 0.78 | 393 | 5.11E-03 | 2.54E-03 | 0 | UP |
| HEL308 | 4 | 0 | 493 | 0.0058844 | 437 | 0.001632974 | 0.78 | 393 | 5.11E-03 | 2.54E-03 | 0 | UP |
| QSER1 | 4 | 0 | 494 | 0.0058844 | 438 | 0.001632974 | 0.78 | 393 | 5.11E-03 | 2.54E-03 | 0 | UP |
| NRCAM | 4 | 0 | 495 | 0.0058844 | 439 | 0.001632974 | 0.78 | 393 | 5.11E-03 | 2.54E-03 | 0 | UP |
| KDELC1 | 4 | 0 | 496 | 0.0058844 | 440 | 0.001632974 | 0.78 | 393 | 5.11E-03 | 2.54E-03 | 0 | UP |
| PHF8 | 4 | 0 | 497 | 0.0058844 | 441 | 0.001632974 | 0.78 | 393 | 5.11E-03 | 2.54E-03 | 0 | UP |
| SERPINB5 | 4 | 0 | 498 | 0.0058844 | 442 | 0.001632974 | 0.78 | 393 | 5.11E-03 | 2.54E-03 | 0 | UP |
| CEP135 | 4 | 0 | 499 | 0.0058844 | 443 | 0.001632974 | 0.78 | 393 | 5.11E-03 | 2.54E-03 | 0 | UP |
| EVI1 | 4 | 0 | 500 | 0.0058844 | 444 | 0.001632974 | 0.78 | 393 | 5.11E-03 | 2.54E-03 | 0 | UP |
| ZNF346 | 4 | 0 | 501 | 0.0058844 | 445 | 0.001632974 | 0.78 | 393 | 5.11E-03 | 2.54E-03 | 0 | UP |
| HSPA5 | 7 | 102 | 502 | 0.0059133 | 533 | 0.00602398 | 0.95 | 231 | 1.17E-05 | 1.08E-03 | 1 | down |
| RPL17 | 57 | 137 | 503 | 0.0062611 | 516 | 0.005842 | 0.01 | 501 | 3.06E-02 | 4.41E-03 | 0 | UP |
| LTBR | 8 | 4 | 504 | 0.0062611 | 482 | 0.002966014 | 0.86 | 371 | 2.66E-03 | 2.25E-03 | 0 | UP |
| CUL4B | 8 | 4 | 505 | 0.0062611 | 483 | 0.002966014 | 0.86 | 371 | 2.66E-03 | 2.25E-03 | 0 | UP |
| SDF4 | 8 | 4 | 506 | 0.0062611 | 484 | 0.002966014 | 0.86 | 371 | 2.66E-03 | 2.25E-03 | 0 | UP |
| RHOBTB1 | 8 | 4 | 507 | 0.0062611 | 485 | 0.002966014 | 0.86 | 371 | 2.66E-03 | 2.25E-03 | 0 | UP |
| C14orf78 | 8 | 4 | 508 | 0.0062611 | 486 | 0.002966014 | 0.86 | 371 | 2.66E-03 | 2.25E-03 | 0 | UP |
| MBTPS1 | 8 | 4 | 509 | 0.0062611 | 487 | 0.002966014 | 0.86 | 371 | 2.66E-03 | 2.25E-03 | 0 | UP |
| ARHGAP18 | 8 | 4 | 510 | 0.0062611 | 488 | 0.002966014 | 0.86 | 371 | 2.66E-03 | 2.25E-03 | 0 | UP |
| CDCA8 | 8 | 4 | 511 | 0.0062611 | 489 | 0.002966014 | 0.86 | 371 | 2.66E-03 | 2.25E-03 | 0 | UP |
| GTF3C5 | 8 | 4 | 512 | 0.0062611 | 490 | 0.002966014 | 0.86 | 371 | 2.66E-03 | 2.25E-03 | 0 | UP |
| CAPZB | 1 | 48 | 513 | 0.0063084 | 593 | 0.006157988 | 0.95 | 278 | 1.16E-04 | 1.42E-03 | 1 | down |
| MVP | 15 | 17 | 514 | 0.0063686 | 613 | 0.006962188 | 0.73 | 385 | 4.01E-03 | 2.40E-03 | 0 | UP |
| UBE1C | 11 | 9 | 515 | 0.0065274 | 479 | 0.002906643 | 0.82 | 375 | 3.18E-03 | 2.31E-03 | 0 | UP |
| ENC1 | 14 | 15 | 516 | 0.0067702 | 602 | 0.006410811 | 0.75 | 384 | 3.97E-03 | 2.39E-03 | 0 | UP |
| DSP | 0 | 32 | 517 | 0.0067823 | 632 | 0.008135619 | 0.92 | 319 | 5.78E-04 | 1.72E-03 | 1 | down |
| CHMP5 | 0 | 32 | 518 | 0.0067823 | 633 | 0.008135619 | 0.92 | 319 | 5.78E-04 | 1.72E-03 | 1 | down |
| SCP2 | 0 | 32 | 519 | 0.0067823 | 634 | 0.008135619 | 0.92 | 319 | 5.78E-04 | 1.72E-03 | 1 | down |
| PIGP | 0 | 32 | 520 | 0.0067823 | 635 | 0.008135619 | 0.92 | 319 | 5.78E-04 | 1.72E-03 | 1 | down |
| IFI30 | 0 | 32 | 521 | 0.0067823 | 636 | 0.008135619 | 0.92 | 319 | 5.78E-04 | 1.72E-03 | 1 | down |
| ITGA3 | 0 | 32 | 522 | 0.0067823 | 637 | 0.008135619 | 0.92 | 319 | 5.78E-04 | 1.72E-03 | 1 | down |
| RFC2 | 0 | 32 | 523 | 0.0067823 | 638 | 0.008135619 | 0.92 | 319 | 5.78E-04 | 1.72E-03 | 1 | down |
| MAN2B1 | 13 | 13 | 524 | 0.0068919 | 535 | 0.00602398 | 0.77 | 382 | 3.83E-03 | 2.38E-03 | 0 | UP |
| GNG12 | 13 | 13 | 525 | 0.0068919 | 536 | 0.00602398 | 0.77 | 382 | 3.83E-03 | 2.38E-03 | 0 | UP |
| MGC23909 | 1 | 47 | 526 | 0.0074906 | 629 | 0.008038445 | 0.95 | 282 | 1.47E-04 | 1.44E-03 | 1 | down |
| AMZ2 | 0 | 31 | 527 | 0.008333 | 657 | 0.0093633 | 0.91 | 326 | 7.45E-04 | 1.80E-03 | 1 | down |
| LOC643788 | 0 | 31 | 528 | 0.008333 | 658 | 0.0093633 | 0.91 | 326 | 7.45E-04 | 1.80E-03 | 1 | down |
| PPP4R1 | 0 | 31 | 529 | 0.008333 | 659 | 0.0093633 | 0.91 | 326 | 7.45E-04 | 1.80E-03 | 1 | down |
| STARD3NL | 0 | 31 | 530 | 0.008333 | 660 | 0.0093633 | 0.91 | 326 | 7.45E-04 | 1.80E-03 | 1 | down |
| SND1 | 21 | 32 | 531 | 0.0083384 | 630 | 0.008122614 | 0.52 | 413 | 7.54E-03 | 2.93E-03 | 0 | UP |
| COTL1 | 2 | 57 | 532 | 0.0085771 | 631 | 0.008135619 | 0.95 | 270 | 9.66E-05 | 1.36E-03 | 1 | down |
| CAP1 | 34 | 68 | 533 | 0.008669 | 641 | 0.008459551 | 0.17 | 457 | 1.58E-02 | 3.38E-03 | 0 | UP |
| PARN | 7 | 3 | 534 | 0.008669 | 496 | 0.00390816 | 0.84 | 380 | 3.62E-03 | 2.34E-03 | 0 | UP |
| WWTR1 | 7 | 3 | 535 | 0.008669 | 497 | 0.00390816 | 0.84 | 380 | 3.62E-03 | 2.34E-03 | 0 | UP |
| EGLN2 | 7 | 3 | 536 | 0.008669 | 498 | 0.00390816 | 0.84 | 380 | 3.62E-03 | 2.34E-03 | 0 | UP |
| ELK3 | 7 | 3 | 537 | 0.008669 | 499 | 0.00390816 | 0.84 | 380 | 3.62E-03 | 2.34E-03 | 0 | UP |
| PPP2R1B | 7 | 3 | 538 | 0.008669 | 500 | 0.00390816 | 0.84 | 380 | 3.62E-03 | 2.34E-03 | 0 | UP |
| THRAP4 | 9 | 6 | 539 | 0.008669 | 503 | 0.004528686 | 0.83 | 381 | 3.82E-03 | 2.36E-03 | 0 | UP |
| SPTLC1 | 9 | 6 | 540 | 0.008669 | 504 | 0.004528686 | 0.83 | 381 | 3.82E-03 | 2.36E-03 | 0 | UP |
| MYD88 | 9 | 6 | 541 | 0.008669 | 505 | 0.004528686 | 0.83 | 381 | 3.82E-03 | 2.36E-03 | 0 | UP |
| SCARB2 | 9 | 6 | 542 | 0.008669 | 506 | 0.004528686 | 0.83 | 381 | 3.82E-03 | 2.36E-03 | 0 | UP |
| TSN | 9 | 6 | 543 | 0.008669 | 507 | 0.004528686 | 0.83 | 381 | 3.82E-03 | 2.36E-03 | 0 | UP |
| CNIH | 4 | 75 | 544 | 0.008669 | 639 | 0.008425062 | 0.95 | 256 | 4.70E-05 | 1.22E-03 | 1 | down |
| FLNA | 4 | 75 | 545 | 0.008669 | 640 | 0.008425062 | 0.95 | 256 | 4.70E-05 | 1.22E-03 | 1 | down |
| IGFBP3 | 1 | 46 | 546 | 0.0087842 | 651 | 0.008986217 | 0.94 | 287 | 1.85E-04 | 1.47E-03 | 1 | down |
| PXDN | 15 | 18 | 547 | 0.0093145 | 692 | 0.013095442 | 0.69 | 401 | 5.83E-03 | 2.82E-03 | 0 | UP |
| FAU | 18 | 25 | 548 | 0.0093503 | 669 | 0.010770807 | 0.6 | 410 | 7.08E-03 | 2.87E-03 | 0 | UP |
| RPL5 | 46 | 105 | 549 | 0.0093984 | 650 | 0.008661835 | 0.03 | 495 | 2.85E-02 | 4.35E-03 | 0 | UP |
| SMARCA4 | 20 | 30 | 550 | 0.0095408 | 661 | 0.009459994 | 0.53 | 418 | 8.17E-03 | 2.99E-03 | 0 | UP |
| SSB | 0 | 30 | 551 | 0.00993 | 673 | 0.011680036 | 0.9 | 334 | 9.61E-04 | 1.87E-03 | 1 | down |
| IDH3B | 0 | 30 | 552 | 0.00993 | 674 | 0.011680036 | 0.9 | 334 | 9.61E-04 | 1.87E-03 | 1 | down |
| RASSF2 | 0 | 30 | 553 | 0.00993 | 675 | 0.011680036 | 0.9 | 334 | 9.61E-04 | 1.87E-03 | 1 | down |
| ZC3H11A | 0 | 30 | 554 | 0.00993 | 676 | 0.011680036 | 0.9 | 334 | 9.61E-04 | 1.87E-03 | 1 | down |
