# Supplementary material for: A novel method of differential gene expression analysis using multiple cDNA libraries applied to the identification of tumour endothelial genes
Source: BMC Genomics. 2008 Apr 7;9:153. doi: 10.1186/1471-2164-9-153 (PMC2346479; doi:10.1186/1471-2164-9-153)
Supplement: Additional file 8 — From the latest available endothelial cDNA library data, 431 genes predicted to be statistically significantly up regulated in endothelial cells. 104 genes showed an endothelial specific profile. [file 1471-2164-9-153-S8.doc]

**Additional File 8:** From the latest available endothelial cDNA library data, 431 genes were predicted to be statistically significantly up regulated in endothelial cells. 104 genes showed an endothelial specific profile. 284 of the 431 up regulated endothelial genes have a posterior probability >= 0.9 that a gene is at least 2 fold up-regulated in endothelial cells (SAGE xProfiler statistics).

| **Gene** | **q-value** | **Endo- ESTs** | **Non-Endo ESTs** | **Product** | **Nucleotide** | **Posterior**  **Probability 2fold** |
| --- | --- | --- | --- | --- | --- | --- |
| *MMP1* | 0.0000 | 203 | 0 | matrix metalloproteinase 1 preproprotein | NM_002421 | 1 |
| *ROBO4* | 0.0000 | 130 | 0 | roundabout homolog 4, magic roundabout | NM_019055 | 1 |
| *SPARCL1* | 0.0000 | 97 | 0 | SPARC-like 1 | NM_004684 | 1 |
| *VWF* | 0.0000 | 73 | 0 | von Willebrand factor preproprotein von Willebrand | NM_000552 | 1 |
| *HHIP* | 0.0000 | 61 | 0 | hedgehog-interacting protein | NM_022475 | 1 |
| *C9orf26* | 0.0000 | 33 | 0 | interleukin 33 | NM_033439 | 1 |
| *RHOJ* | 0.0000 | 31 | 0 | TC10-like Rho GTPase | NM_020663 | 1 |
| *BMX* | 0.0000 | 30 | 0 | BMX non-receptor tyrosine kinase | NM_001721 NM_203281 | 1 |
| *ELTD1* | 0.0000 | 29 | 0 | EGF, latrophilin and seven transmembrane domain co | XM_371262 XM_942048 | 1 |
| *MMRN1* | 0.0000 | 26 | 0 | multimerin 1 | NM_007351 | 1 |
| *EMCN* | 0.0000 | 24 | 0 | endomucin | NM_016242 | 1 |
| *CDH5* | 0.0000 | 23 | 0 | cadherin 5, type 2 preproprotein cadherin 5, type | NM_001795 | 1 |
| *SOX7* | 0.0000 | 20 | 0 | SRY-box 7 | NM_031439 | 1 |
| *ARHGAP24* | 0.0000 | 18 | 0 | Rho GTPase activating protein 24 | NM_031305 NM_001025616 | 1 |
| *FGD5* | 0.0000 | 18 | 0 | FYVE, RhoGEF and PH domain containing 5 | NM_152536 | 1 |
| *PCDH12* | 0.0000 | 18 | 0 | protocadherin 12 precursor protocadherin 12 | NM_016580 | 1 |
| *CD93* | 0.0000 | 17 | 0 | CD93 antigen precursor CD93 antigen | NM_012072 | 1 |
| *ERG* | 0.0000 | 16 | 0 | v-ets erythroblastosis virus E26 oncogene like iso | NM_182918 NM_004449 | 1 |
| *MYCT1* | 0.0000 | 16 | 0 | myc target 1 | NM_025107 | 1 |
| *FLJ22746* | 0.0000 | 15 | 0 | hypothetical protein LOC79843 | NM_024785 | 1 |
| *SELE* | 0.0000 | 14 | 0 | selectin E precursor selectin E | NM_000450 | 1 |
| *ANGPT2* | 0.0000 | 13 | 0 | angiopoietin 2 | NM_001147 | 1 |
| *TCF4* | 0.0000 | 13 | 0 | transcription factor 4 isoform b | NM_003199 | 1 |
| *EDG1* | 0.0000 | 12 | 0 | endothelial differentiation, sphingolipid G-protei | NM_001400 | 1 |
| *SDPR* | 0.0000 | 12 | 0 | serum deprivation response protein | NM_004657 | 1 |
| *ARHGEF15* | 0.0000 | 11 | 0 | Rho guanine exchange factor 15 | NM_173728 | 1 |
| *GIMAP6* | 0.0000 | 11 | 0 | GTPase, IMAP family member 6 isoform 1 | NM_024711 NM_001007224 | 1 |
| *NOD27* | 0.0000 | 10 | 0 | nucleotide-binding oligomerization domains 27 | NM_032206 | 1 |
| *PLA2G4C* | 0.0000 | 10 | 0 | phospholipase A2, group IVC | NM_003706 | 1 |
| *RAD54L2* | 0.0000 | 10 | 0 | RAD54-like 2 | NM_015106 | 1 |
| *RAPGEF3* | 0.0000 | 10 | 0 | RAP guanine-nucleotide-exchange factor 3 | NM_006105 | 1 |
| *ECSM2* | 0.0000 | 9 | 0 | Endothelial Cell Specific Molecule 2 | DQ462572 | 0.99 |
| *CALCRL* | 0.0000 | 9 | 0 | calcitonin receptor-like | NM_005795 | 0.99 |
| *CD34* | 0.0000 | 9 | 0 | CD34 antigen isoform a | NM_001025109 NM_001773 | 0.99 |
| *FZD4* | 0.0000 | 9 | 0 | frizzled 4 | NM_012193 | 0.99 |
| *NOSTRIN* | 0.0000 | 9 | 0 | nitric oxide synthase trafficking isoform 1 | NM_052946 NM_001039724 | 0.99 |
| *SLC35A2* | 0.0000 | 9 | 0 | solute carrier family 35 member A2 isoform a | NM_005660 NM_001032289 NM_001042498 | 0.99 |
| *SMURF2* | 0.0000 | 9 | 0 | SMAD specific E3 ubiquitin protein ligase 2 | NM_022739 | 0.99 |
| *SRPX* | 0.0000 | 9 | 0 | sushi-repeat-containing protein, X-linked | NM_006307 | 0.99 |
| *CCL20* | 0.0000 | 8 | 0 | chemokine (C-C motif) ligand 20 CC chemokine LARC | NM_004591 | 0.98 |
| *LIFR* | 0.0000 | 8 | 0 | leukemia inhibitory factor receptor precursor leuk | NM_002310 | 0.98 |
| *LRRC8C* | 0.0000 | 8 | 0 | factor for adipocyte differentiation 158 | NM_032270 | 0.98 |
| *MUS81* | 0.0000 | 8 | 0 | MUS81 endonuclease homolog | NM_025128 | 0.98 |
| *THBD* | 0.0000 | 8 | 0 | thrombomodulin precursor thrombomodulin | NM_000361 | 0.98 |
| *ACVRL1* | 0.0001 | 7 | 0 | activin A receptor type II-like 1 activin A recept | NM_000020 | 0.96 |
| *ATP5H* | 0.0001 | 7 | 0 | ATP synthase, H+ transporting, mitochondrial F0 co | NM_006356 NM_001003785 | 0.96 |
| *C7* | 0.0001 | 7 | 0 | complement component 7 precursor complement compon | NM_000587 | 0.96 |
| *ECSM1* | 0.0001 | 7 | 0 | Endothelial Cell Specific Molecule 1 | BC070102 | 0.96 |
| *FLJ39531* | 0.0001 | 7 | 0 | hypothetical protein LOC400360 | NM_207445 | 0.96 |
| *LOC650049* | 0.0001 | 7 | 0 | hypothetical protein XP_944228 | XM_939135 | 0.96 |
| *TNFSF18* | 0.0001 | 7 | 0 | tumor necrosis factor (ligand) superfamily, member | NM_005092 | 0.96 |
| *TNNC2* | 0.0001 | 7 | 0 | fast skeletal muscle troponin C | NM_003279 | 0.96 |
| *UACA* | 0.0001 | 7 | 0 | uveal autoantigen with coiled-coil domains and ank | NM_018003 NM_001008224 | 0.96 |
| *VEPH1* | 0.0001 | 7 | 0 | ventricular zone expressed PH domain homolog 1 | NM_024621 | 0.96 |
| *ACP2* | 0.0003 | 6 | 0 | lysosomal acid phosphatase 2 precursor lysosomal a | NM_001610 | 0.93 |
| *ACTA1* | 0.0003 | 6 | 0 | alpha 1 actin precursor | NM_001100 | 0.93 |
| *APLN* | 0.0003 | 6 | 0 | apelin preproprotein | NM_017413 | 0.93 |
| *CCDC99* | 0.0003 | 6 | 0 | coiled-coil domain containing 99 | NM_017785 | 0.93 |
| *LOC152485* | 0.0003 | 6 | 0 | hypothetical protein LOC152485 | NM_178835 | 0.93 |
| *MOV10L1* | 0.0003 | 6 | 0 | MOV10-like 1 | NM_018995 | 0.93 |
| *PCBP4* | 0.0003 | 6 | 0 | poly(rC) binding protein 4 isoform c | NM_033008 NM_033009 NM_033010 NM_020418 | 0.93 |
| *SNX12* | 0.0003 | 6 | 0 | sorting nexin 12 | NM_013346 | 0.93 |
| *TAF15* | 0.0003 | 6 | 0 | TBP-associated factor 15 isoform 2 | NM_003487 NM_139215 | 0.93 |
| *ZNF521* | 0.0003 | 6 | 0 | zinc finger protein 521 | NM_015461 | 0.93 |
| *ANTXR2* | 0.0013 | 5 | 0 | anthrax toxin receptor 2 | NM_058172 | 0.87 |
| *BMP6* | 0.0013 | 5 | 0 | bone morphogenetic protein 6 precursor bone morpho | NM_001718 | 0.87 |
| *C16orf30* | 0.0013 | 5 | 0 | claudin-like protein 24 | NM_024600 | 0.87 |
| *GBP4* | 0.0013 | 5 | 0 | guanylate binding protein 4 | NM_052941 | 0.87 |
| *IL1RL1* | 0.0013 | 5 | 0 | interleukin 1 receptor-like 1 isoform 2 precursor | NM_003856 NM_016232 | 0.87 |
| *JAM2* | 0.0013 | 5 | 0 | junctional adhesion molecule 2 precursor junctiona | NM_021219 | 0.87 |
| *LOC650626* | 0.0013 | 5 | 0 | similar to WDNM1 homolog | XM_939715 | 0.87 |
| *MAP3K8* | 0.0013 | 5 | 0 | mitogen-activated protein kinase kinase kinase 8 | NM_005204 | 0.87 |
| *NUP54* | 0.0013 | 5 | 0 | nucleoporin 54kDa | NM_017426 | 0.87 |
| *RHOBTB3* | 0.0013 | 5 | 0 | rho-related BTB domain containing 3 | NM_014899 | 0.87 |
| *RNASE1* | 0.0013 | 5 | 0 | pancreatic ribonuclease precursor ribonuclease, RN | NM_198232 NM_198234 NM_198235 NM_002933 | 0.87 |
| *SLC39A9* | 0.0013 | 5 | 0 | solute carrier family 39 (zinc transporter), membe | NM_018375 | 0.87 |
| *SPINK5* | 0.0013 | 5 | 0 | serine peptidase inhibitor, Kazal type 5 precursor | NM_006846 | 0.87 |
| *TMEM154* | 0.0013 | 5 | 0 | hypothetical protein LOC201799 | NM_152680 | 0.87 |
| *CEP135* | 0.0059 | 4 | 0 | centrosome protein 4 | NM_025009 | 0.78 |
| *EVI1* | 0.0059 | 4 | 0 | ecotropic viral integration site 1 | NM_005241 | 0.78 |
| *FABP4* | 0.0059 | 4 | 0 | fatty acid binding protein 4, adipocyte | NM_001442 | 0.78 |
| *FBXL3* | 0.0059 | 4 | 0 | F-box and leucine-rich repeat protein 3 | NM_012158 | 0.78 |
| *FNTB* | 0.0059 | 4 | 0 | farnesyltransferase, CAAX box, beta | NM_002028 | 0.78 |
| *GPR4* | 0.0059 | 4 | 0 | G protein-coupled receptor 4 | NM_005282 | 0.78 |
| *GTF3C4* | 0.0059 | 4 | 0 | general transcription factor IIIC, polypeptide 4, | NM_012204 | 0.78 |
| *HEL308* | 0.0059 | 4 | 0 | DNA helicase HEL308 | NM_133636 | 0.78 |
| *HEY1* | 0.0059 | 4 | 0 | hairy/enhancer-of-split related with YRPW motif 1 | NM_001040708 NM_012258 | 0.78 |
| *KDELC1* | 0.0059 | 4 | 0 | KDEL (Lys-Asp-Glu-Leu) containing 1 | NM_024089 | 0.78 |
| *MMRN2* | 0.0059 | 4 | 0 | multimerin 2 | NM_024756 | 0.78 |
| *RNU2* | 0.0059 | 4 | 0 | RNA, U2 small nuclear (RNU2) | NR_002716 | 0.78 |
| *NRCAM* | 0.0059 | 4 | 0 | neuronal cell adhesion molecule isoform C | NM_001037133 NM_005010 NM_001037132 | 0.78 |
| *PHF8* | 0.0059 | 4 | 0 | PHD finger protein 8 | NM_015107 | 0.78 |
| *PI4K2B* | 0.0059 | 4 | 0 | phosphatidylinositol 4-kinase type-II beta | NM_018323 | 0.78 |
| *QSER1* | 0.0059 | 4 | 0 | hypothetical protein LOC79832 | NM_024774 | 0.78 |
| *RGS1* | 0.0059 | 4 | 0 | regulator of G-protein signalling 1 | NM_002922 | 0.78 |
| *SERPINB5* | 0.0059 | 4 | 0 | serine (or cysteine) proteinase inhibitor, clade B | NM_002639 | 0.78 |
| *SHQ1* | 0.0059 | 4 | 0 | SHQ1 homolog | NM_018130 | 0.78 |
| *SLC35A5* | 0.0059 | 4 | 0 | solute carrier family 35, member A5 | NM_017945 | 0.78 |
| *STARD4* | 0.0059 | 4 | 0 | START domain containing 4, sterol regulated | NM_139164 | 0.78 |
| *TEK* | 0.0059 | 4 | 0 | TEK tyrosine kinase, endothelial precursor TEK tyr | NM_000459 | 0.78 |
| *TM6SF1* | 0.0059 | 4 | 0 | transmembrane 6 superfamily member 1 | NM_023003 | 0.78 |
| *XLKD1* | 0.0059 | 4 | 0 | extracellular link domain containing 1 | NM_006691 | 0.78 |
| *ZNF346* | 0.0059 | 4 | 0 | zinc finger protein 346 | NM_012279 | 0.78 |
| *ZNF586* | 0.0059 | 4 | 0 | zinc finger protein 586 | NM_017652 | 0.78 |
| *ENG* | 0.0000 | 149 | 2 | endoglin precursor endoglin | NM_000118 | 1 |
| *A2M* | 0.0000 | 143 | 3 | alpha-2-macroglobulin precursor alpha-2-macroglobu | NM_000014 | 1 |
| *MCAM* | 0.0000 | 156 | 9 | melanoma cell adhesion molecule | NM_006500 | 1 |
| *PRG1* | 0.0000 | 159 | 15 | proteoglycan 1, secretory granule precursor proteo | NM_002727 | 1 |
| *TGM2* | 0.0000 | 209 | 27 | transglutaminase 2 isoform b | NM_198951 NM_004613 | 1 |
| *APLP2* | 0.0000 | 178 | 41 | amyloid beta (A4) precursor-like protein 2 | NM_001642 | 1 |
| *CTGF* | 0.0000 | 202 | 58 | connective tissue growth factor | NM_001901 | 1 |
| *HDLBP* | 0.0000 | 164 | 61 | high density lipoprotein binding protein | NM_005336 NM_203346 | 1 |
| *EMP1* | 0.0000 | 136 | 30 | epithelial membrane protein 1 | NM_001423 | 1 |
| *ITGA5* | 0.0000 | 113 | 15 | integrin alpha 5 precursor integrin alpha 5 | NM_002205 | 1 |
| *EIF4G2* | 0.0000 | 156 | 73 | eukaryotic translation initiation factor 4 gamma, | NM_001418 | 1 |
| *HSPA8* | 0.0000 | 251 | 243 | heat shock 70kDa protein 8 isoform 1 | NM_006597 NM_153201 | 1 |
| *VIM* | 0.0000 | 209 | 181 | vimentin | NM_003380 | 1 |
| *BGN* | 0.0000 | 82 | 6 | biglycan preproprotein biglycan proprotein biglyca | NM_001711 | 1 |
| *SGK* | 0.0000 | 86 | 9 | serum/glucocorticoid regulated kinase | NM_005627 | 1 |
| *CAV1* | 0.0000 | 80 | 8 | caveolin 1 | NM_001753 | 1 |
| *SERPINE1* | 0.0000 | 97 | 30 | plasminogen activator inhibitor-1 plasminogen acti | NM_000602 | 1 |
| *PTRF* | 0.0000 | 98 | 34 | polymerase I and transcript release factor | NM_012232 | 1 |
| *NCL* | 0.0000 | 85 | 30 | nucleolin | NM_005381 | 1 |
| *S100A8* | 0.0000 | 48 | 1 | S100 calcium-binding protein A8 | NM_002964 | 1 |
| *RHOB* | 0.0000 | 54 | 6 | ras homolog gene family, member B | NM_004040 | 1 |
| *DYSF* | 0.0000 | 48 | 3 | dysferlin | NM_003494 | 1 |
| *ENPP2* | 0.0000 | 43 | 1 | autotaxin isoform 2 preproprotein autotaxin isofor | NM_001040092 NM_006209 | 1 |
| *DCTN1* | 0.0000 | 61 | 20 | dynactin 1 isoform 1 | NM_004082 NM_023019 | 1 |
| *PECAM1* | 0.0000 | 39 | 1 | platelet/endothelial cell adhesion molecule (CD31 | NM_000442 | 1 |
| *TMSB4X* | 0.0000 | 120 | 121 | thymosin, beta 4 | NM_021109 | 1 |
| *RGS4* | 0.0000 | 51 | 11 | regulator of G-protein signaling 4 | NM_005613 | 1 |
| *GRN* | 0.0000 | 57 | 19 | granulin isoform 1 precursor granulin isoform 1 | NM_002087 NM_001012479 | 1 |
| *RNF40* | 0.0000 | 46 | 8 | ring finger protein 40 | NM_014771 | 1 |
| *S100A6* | 0.0000 | 58 | 25 | S100 calcium-binding protein A6 | NM_014624 | 1 |
| *EFEMP1* | 0.0000 | 47 | 13 | EGF-containing fibulin-like extracellular matrix p | NM_001039348 NM_004105 NM_001039349 | 1 |
| *HYOU1* | 0.0000 | 43 | 11 | oxygen regulated protein precursor oxygen regulate | NM_006389 | 1 |
| *RPL26* | 0.0000 | 76 | 60 | ribosomal protein L26 | NM_000987 | 1 |
| *ANXA2* | 0.0000 | 178 | 304 | annexin A2 isoform 2 | NM_001002857 NM_001002858 NM_004039 | 0.49 |
| *ESM1* | 0.0000 | 29 | 1 | endothelial cell-specific molecule 1 precursor end | NM_007036 | 1 |
| *ZNF207* | 0.0000 | 47 | 19 | zinc finger protein 207 isoform a | NM_003457 NM_001032293 | 1 |
| *UNC45A* | 0.0000 | 34 | 5 | smooth muscle cell associated protein-1 isoform 2 | NM_018671 NM_001039675 | 1 |
| *RPS27A* | 0.0000 | 60 | 43 | ubiquitin and ribosomal protein S27a precursor ubi | NM_002954 | 1 |
| *LMNA* | 0.0000 | 53 | 34 | lamin A/C isoform 2 | NM_005572 NM_170707 NM_170708 | 1 |
| *WARS* | 0.0000 | 39 | 14 | tryptophanyl-tRNA synthetase isoform b | NM_213646 NM_004184 NM_213645 NM_173701 | 1 |
| *RPN1* | 0.0000 | 36 | 11 | ribophorin I precursor ribophorin I | NM_002950 | 1 |
| *GNAI3* | 0.0000 | 39 | 15 | guanine nucleotide binding protein (G protein), al | NM_006496 | 1 |
| *VAMP3* | 0.0000 | 35 | 11 | vesicle-associated membrane protein 3 (cellubrevin | NM_004781 | 1 |
| *NDRG1* | 0.0000 | 33 | 9 | N-myc downstream regulated gene 1 | NM_006096 | 1 |
| *RPL19* | 0.0000 | 61 | 52 | ribosomal protein L19 | NM_000981 | 1 |
| *ADAM15* | 0.0000 | 25 | 2 | a disintegrin and metalloproteinase domain 15 isof | NM_207191 NM_207195 NM_207196 NM_207194 NM_003815 NM_207197 | 1 |
| *RPL37A* | 0.0000 | 59 | 52 | ribosomal protein L37a | NM_000998 | 1 |
| *PLOD1* | 0.0000 | 33 | 11 | lysyl hydroxylase precursor lysyl hydroxylase | NM_000302 | 1 |
| *ECE1* | 0.0000 | 32 | 10 | endothelin converting enzyme 1 | NM_001397 | 1 |
| *OS9* | 0.0000 | 29 | 7 | amplified in osteosarcoma isoform 2 precursor ampl | NM_001017956 NM_006812 NM_001017958 NM_001017957 | 1 |
| *XRCC6* | 0.0000 | 60 | 58 | ATP-dependent DNA helicase II, 70 kDa subunit | NM_001469 | 1 |
| *DYNLL1* | 0.0000 | 33 | 13 | dynein light chain 1 | NM_001037494 NM_003746 NM_001037495 | 1 |
| *TMSB10* | 0.0000 | 45 | 31 | thymosin, beta 10 | NM_021103 | 1 |
| *C4orf18* | 0.0000 | 22 | 2 | hypothetical protein LOC51313 isoform 2 | NM_016613 NM_001031700 | 1 |
| *CALU* | 0.0000 | 49 | 39 | calumenin precursor calumenin | NM_001219 | 1 |
| *SERPINH1* | 0.0000 | 44 | 31 | serine (or cysteine) proteinase inhibitor, clade H | NM_001235 | 1 |
| *FLNB* | 0.0000 | 39 | 23 | filamin B, beta (actin binding protein 278) | NM_001457 | 1 |
| *GJA1* | 0.0000 | 37 | 21 | connexin 43 | NM_000165 | 1 |
| *PPP2R1A* | 0.0000 | 43 | 31 | alpha isoform of regulatory subunit A, protein pho | NM_014225 | 1 |
| *PLS3* | 0.0000 | 36 | 20 | plastin 3 | NM_005032 | 1 |
| *WDR6* | 0.0000 | 32 | 15 | WD repeat domain 6 protein | NM_018031 | 1 |
| *MANSC1* | 0.0000 | 20 | 2 | MANSC domain containing 1 | NM_018050 | 1 |
| *SLC29A1* | 0.0000 | 20 | 2 | solute carrier family 29 (nucleoside transporters) | NM_004955 | 1 |
| *RPS20* | 0.0000 | 64 | 75 | ribosomal protein S20 | NM_001023 | 0.98 |
| *GANAB* | 0.0000 | 37 | 23 | alpha glucosidase II alpha subunit isoform 3 | NM_198335 NM_198334 | 1 |
| *TPD52L2* | 0.0000 | 25 | 7 | tumor protein D52-like 2 isoform e | NM_003288 NM_199359 NM_199360 NM_199361 NM_199362 NM_199363 | 1 |
| *PRCP* | 0.0000 | 24 | 6 | prolylcarboxypeptidase isoform 2 | NM_199418 NM_005040 | 1 |
| *NONO* | 0.0000 | 61 | 70 | non-POU domain containing, octamer-binding | NM_007363 | 0.98 |
| *RPAP1* | 0.0000 | 18 | 1 | RNA polymerase II associated protein 1 | NM_015540 | 1 |
| *DYNC1I2* | 0.0000 | 29 | 12 | dynein, cytoplasmic, intermediate polypeptide 2 | NM_001378 | 1 |
| *MYO1C* | 0.0000 | 27 | 10 | myosin IC | NM_033375 | 1 |
| *TNPO1* | 0.0000 | 19 | 2 | transportin 1 | NM_002270 NM_153188 | 1 |
| *RPL31* | 0.0000 | 55 | 59 | ribosomal protein L31 | NM_000993 | 0.99 |
| *WDR1* | 0.0000 | 45 | 39 | WD repeat-containing protein 1 isoform 1 | NM_017491 NM_005112 | 1 |
| *IKBKE* | 0.0000 | 21 | 4 | IKK-related kinase epsilon | NM_014002 | 1 |
| *SMG6* | 0.0000 | 21 | 4 | Smg-6 homolog, nonsense mediated mRNA decay factor | NM_017575 | 1 |
| *SPRR3* | 0.0000 | 17 | 1 | small proline-rich protein 3 | NM_005416 | 1 |
| *RPL32* | 0.0000 | 37 | 26 | ribosomal protein L32 | NM_000994 NM_001007073 NM_001007074 | 1 |
| *CYR61* | 0.0000 | 37 | 27 | cysteine-rich, angiogenic inducer, 61 | NM_001554 | 1 |
| *DUSP6* | 0.0000 | 19 | 3 | dual specificity phosphatase 6 isoform b | NM_022652 NM_001946 | 1 |
| *UBC* | 0.0000 | 89 | 147 | ubiquitin C | NM_021009 | 0.55 |
| *C10orf10* | 0.0000 | 16 | 1 | fasting induced gene | NM_007021 | 1 |
| *FAM62A* | 0.0000 | 25 | 10 | family with sequence similarity 62 (C2 domain cont | NM_015292 | 1 |
| *RPS12* | 0.0000 | 42 | 38 | ribosomal protein S12 | NM_001016 | 0.99 |
| *ACTR3* | 0.0000 | 30 | 18 | ARP3 actin-related protein 3 homolog | NM_005721 | 1 |
| *MMS19L* | 0.0000 | 21 | 6 | MMS19-like (MET18 homolog, S. cerevisiae) | NM_022362 | 1 |
| *SDCBP* | 0.0000 | 25 | 11 | syntenin isoform 3 | NM_001007069 NM_001007067 NM_001007070 NM_005625 NM_001007068 | 1 |
| *TXNDC5* | 0.0000 | 34 | 25 | thioredoxin domain containing 5 isoform 1 | NM_030810 NM_022085 | 1 |
| *KIAA0174* | 0.0000 | 35 | 27 | putative MAPK activating protein PM28 | NM_014761 | 1 |
| *RPS8* | 0.0000 | 69 | 103 | ribosomal protein S8 | NM_001012 | 0.75 |
| *THBS1* | 0.0000 | 40 | 38 | thrombospondin 1 precursor thrombospondin 1 | NM_003246 | 0.99 |
| *TIE1* | 0.0000 | 16 | 2 | tyrosine kinase with immunoglobulin-like and EGF-l | NM_005424 | 1 |
| *DAB2* | 0.0000 | 21 | 7 | disabled homolog 2 | NM_001343 | 1 |
| *RPS24* | 0.0000 | 53 | 66 | ribosomal protein S24 isoform c | NM_001026 NM_033022 | 0.93 |
| *SPARC* | 0.0000 | 52 | 64 | secreted protein, acidic, cysteine-rich (osteonect | NM_003118 | 0.93 |
| *GPI* | 0.0000 | 38 | 35 | glucose phosphate isomerase | NM_000175 | 0.99 |
| *RPS18* | 0.0000 | 66 | 99 | ribosomal protein S18 | NM_022551 | 0.74 |
| *TINAGL1* | 0.0000 | 22 | 9 | P3ECSL | NM_022164 | 1 |
| *SEC14L1* | 0.0000 | 14 | 1 | SEC14 (S. cerevisiae)-like 1 isoform a | NM_003003 NM_001039573 | 1 |
| *TMBIM1* | 0.0000 | 26 | 15 | transmembrane BAX inhibitor motif containing 1 | NM_022152 | 1 |
| *PPIA* | 0.0000 | 96 | 182 | peptidylprolyl isomerase A isoform 1 | NM_021130 NM_203430 NM_203431 | 0.18 |
| *VAT1* | 0.0000 | 29 | 20 | vesicle amine transport protein 1 | NM_006373 | 1 |
| *EXOSC10* | 0.0000 | 20 | 7 | exosome component 10 isoform 2 | NM_002685 NM_001001998 | 1 |
| *NRP1* | 0.0000 | 20 | 7 | neuropilin 1 isoform a | NM_003873 NM_001024629 NM_001024628 | 1 |
| *SNAP23* | 0.0000 | 18 | 5 | synaptosomal-associated protein 23 isoform SNAP23B | NM_130798 NM_003825 | 1 |
| *TYK2* | 0.0000 | 18 | 5 | tyrosine kinase 2 | NM_003331 | 1 |
| *EDN1* | 0.0000 | 16 | 3 | endothelin 1 | NM_001955 | 1 |
| *TFPI2* | 0.0000 | 24 | 13 | tissue factor pathway inhibitor 2 | NM_006528 | 1 |
| *DDX5* | 0.0000 | 50 | 65 | DEAD (Asp-Glu-Ala-Asp) box polypeptide 5 | NM_004396 | 0.88 |
| *RPS15A* | 0.0000 | 34 | 31 | ribosomal protein S15a | NM_001030009 NM_001019 | 0.98 |
| *SERINC3* | 0.0000 | 20 | 8 | tumor differentially expressed protein 1 | NM_198941 NM_006811 | 1 |
| *BAG3* | 0.0000 | 18 | 6 | BCL2-associated athanogene 3 | NM_004281 | 1 |
| *F2R* | 0.0000 | 18 | 6 | coagulation factor II receptor precursor proprotei | NM_001992 | 1 |
| *RPS26* | 0.0000 | 21 | 10 | ribosomal protein S26 | NM_001029 | 1 |
| *ADSS* | 0.0000 | 17 | 5 | adenylosuccinate synthase | NM_001126 | 1 |
| *ALDH1A1* | 0.0000 | 17 | 5 | aldehyde dehydrogenase 1A1 | NM_000689 | 1 |
| *GPR177* | 0.0000 | 16 | 4 | G protein-coupled receptor 177 isoform 2 | NM_001002292 NM_024911 | 1 |
| *HNRPH1* | 0.0000 | 26 | 18 | heterogeneous nuclear ribonucleoprotein H1 | NM_005520 | 0.99 |
| *POSTN* | 0.0000 | 19 | 8 | periostin, osteoblast specific factor | NM_006475 | 1 |
| *MAT2A* | 0.0000 | 22 | 13 | methionine adenosyltransferase II, alpha | NM_005911 | 0.99 |
| *RPS13* | 0.0000 | 39 | 46 | ribosomal protein S13 | NM_001017 | 0.91 |
| *FAM43A* | 0.0000 | 13 | 2 | hypothetical protein LOC131583 | NM_153690 | 1 |
| *ICAM2* | 0.0000 | 13 | 2 | intercellular adhesion molecule 2 precursor interc | NM_000873 | 1 |
| *PRSS23* | 0.0000 | 15 | 4 | protease, serine, 23 precursor protease, serine, 2 | NM_007173 | 1 |
| *RALA* | 0.0000 | 15 | 4 | ras related v-ral simian leukemia viral oncogene h | NM_005402 | 1 |
| *LOC653352* | 0.0000 | 44 | 58 | similar to eukaryotic translation initiation facto | XM_930074 XM_934282 XM_934284 XM_934289 XM_934291 XM_934293 XM_934296 XM_934301 XM_934305 XM_934309 XM_934313 XM_934316 XM_934318 XM_934322 XM_934324 XM_934326 | 0.85 |
| *GPR56* | 0.0000 | 14 | 3 | G protein-coupled receptor 56 isoform b | NM_201525 NM_201524 NM_005682 | 1 |
| *RNF4* | 0.0000 | 14 | 3 | ring finger protein 4 | NM_002938 | 1 |
| *LAMA4* | 0.0000 | 19 | 9 | laminin, alpha 4 precursor laminin, alpha 4 | NM_002290 | 0.99 |
| *TXNRD1* | 0.0000 | 24 | 17 | thioredoxin reductase 1 | NM_182743 NM_182729 NM_182742 NM_003330 | 0.99 |
| *PSAP* | 0.0000 | 36 | 41 | saposin | NM_001042465 | 0.43 |
| *NUCB1* | 0.0000 | 23 | 16 | nucleobindin 1 | NM_006184 | 0.99 |
| *CS* | 0.0000 | 24 | 18 | citrate synthase precursor, isoform a citrate synt | NM_004077 NM_198324 | 0.98 |
| *CDC42* | 0.0000 | 19 | 10 | cell division cycle 42 isoform 1 | NM_001791 NM_001039802 NM_044472 | 0.99 |
| *ART4* | 0.0000 | 11 | 1 | ADP-ribosyltransferase 4 precursor Dombrock blood | NM_021071 | 0.99 |
| *KIAA1539* | 0.0000 | 11 | 1 | hypothetical protein LOC80256 | NM_025182 | 0.99 |
| *PLD2* | 0.0000 | 11 | 1 | phospholipase D2 | NM_002663 | 0.99 |
| *MAP1LC3B* | 0.0000 | 18 | 9 | microtubule-associated proteins 1A/1B light chain | NM_022818 | 0.99 |
| *TNFRSF1A* | 0.0000 | 12 | 2 | tumor necrosis factor receptor 1 precursor tumor n | NM_001065 | 0.99 |
| *CASP7* | 0.0000 | 13 | 3 | caspase 7 isoform delta | NM_033338 NM_033340 NM_001227 NM_033339 | 0.99 |
| *TRAM2* | 0.0000 | 13 | 3 | translocation-associated membrane protein 2 | NM_012288 | 0.99 |
| *RPS7* | 0.0000 | 43 | 60 | ribosomal protein S7 | NM_001011 | 0.77 |
| *MMP2* | 0.0000 | 19 | 11 | matrix metalloproteinase 2 preproprotein matrix me | NM_004530 | 0.99 |
| *MAPRE1* | 0.0000 | 21 | 15 | microtubule-associated protein, RP/EB family, memb | NM_012325 | 0.98 |
| *ELOVL5* | 0.0000 | 19 | 12 | homolog of yeast long chain polyunsaturated fatty | NM_021814 | 0.98 |
| *CLIC4* | 0.0000 | 23 | 19 | chloride intracellular channel 4 | NM_013943 | 0.97 |
| *GRWD1* | 0.0000 | 14 | 5 | glutamate-rich WD repeat containing 1 | NM_031485 | 0.99 |
| *MOV10* | 0.0000 | 14 | 5 | Mov10, Moloney leukemia virus 10, homolog | NM_020963 | 0.99 |
| *SURF4* | 0.0000 | 24 | 21 | surfeit 4 | NM_033161 | 0.96 |
| *UBE3C* | 0.0000 | 13 | 4 | ubiquitin protein ligase E3C | NM_014671 | 0.99 |
| *SRPX2* | 0.0000 | 11 | 2 | sushi-repeat-containing protein, X-linked 2 | NM_014467 | 0.99 |
| *RPS15* | 0.0000 | 33 | 42 | ribosomal protein S15 | NM_001018 | 0.82 |
| *RPL24* | 0.0000 | 32 | 40 | ribosomal protein L24 | NM_000986 | 0.83 |
| *HEXB* | 0.0000 | 14 | 6 | hexosaminidase B preproprotein proprotein hexosami | NM_000521 | 0.98 |
| *UBA52* | 0.0000 | 24 | 23 | ubiquitin and ribosomal protein L40 precursor ubiq | NM_003333 NM_001033930 | 0.93 |
| *WASF2* | 0.0000 | 24 | 23 | WAS protein family, member 2 | NM_006990 | 0.93 |
| *RPL27A* | 0.0000 | 37 | 52 | ribosomal protein L27a | NM_000990 | 0.73 |
| *SHC1* | 0.0000 | 20 | 16 | SHC (Src homology 2 domain containing) transformin | NM_183001 NM_003029 | 0.95 |
| *TP53* | 0.0000 | 20 | 16 | tumor protein p53 | NM_000546 | 0.95 |
| *PALMD* | 0.0000 | 9 | 1 | palmdelphin | NM_017734 | 0.98 |
| *PCDH1* | 0.0000 | 9 | 1 | protocadherin 1 isoform 1 precursor protocadherin | NM_002587 NM_032420 | 0.98 |
| *RANBP1* | 0.0000 | 9 | 1 | RAN binding protein 1 | NM_002882 | 0.98 |
| *LDLR* | 0.0000 | 15 | 8 | low density lipoprotein receptor precursor low den | NM_000527 | 0.97 |
| *CNP* | 0.0000 | 12 | 4 | 2',3'-cyclic nucleotide 3' phosphodiesterase | NM_033133 | 0.98 |
| *ABI1* | 0.0000 | 18 | 13 | abl-interactor 1 isoform d | NM_001012752 NM_001012750 NM_001012751 NM_005470 | 0.96 |
| *PMM2* | 0.0001 | 11 | 3 | phosphomannomutase 2 | NM_000303 | 0.98 |
| *SCAMP4* | 0.0001 | 11 | 3 | secretory carrier membrane protein 4 | NM_079834 | 0.98 |
| *CSTA* | 0.0001 | 10 | 2 | cystatin A | NM_005213 | 0.98 |
| *DNAJC10* | 0.0001 | 10 | 2 | DnaJ (Hsp40) homolog, subfamily C, member 10 | NM_018981 | 0.98 |
| *NQO1* | 0.0001 | 16 | 10 | NAD(P)H menadione oxidoreductase 1, dioxin-inducib | NM_000903 NM_001025434 NM_001025433 | 0.97 |
| *LENG4* | 0.0001 | 13 | 6 | leukocyte receptor cluster (LRC) member 4 protein | NM_024298 | 0.97 |
| *FSTL1* | 0.0001 | 21 | 20 | follistatin-like 1 precursor follistatin-like 1 | NM_007085 | 0.91 |
| *SERP1* | 0.0001 | 21 | 20 | stress-associated endoplasmic reticulum protein 1 | NM_014445 | 0.91 |
| *PTTG1IP* | 0.0001 | 37 | 57 | pituitary tumor-transforming gene 1 protein-intera | NM_004339 | 0.59 |
| *NDUFS1* | 0.0001 | 12 | 5 | NADH dehydrogenase (ubiquinone) Fe-S protein 1, 75 | NM_005006 | 0.97 |
| *WSB1* | 0.0001 | 12 | 5 | WD repeat and SOCS box-containing 1 isoform 2 | NM_134265 NM_015626 | 0.97 |
| *EFTUD2* | 0.0002 | 19 | 17 | U5 snRNP-specific protein, 116 kD | NM_004247 | 0.92 |
| *TARDBP* | 0.0002 | 19 | 17 | TAR DNA binding protein | NM_007375 | 0.92 |
| *EXOC6* | 0.0002 | 8 | 1 | SEC15-like 1 isoform b | NM_001013848 NM_019053 | 0.96 |
| *FES* | 0.0002 | 8 | 1 | V-FES feline sarcoma viral/V-FPS fujinami avian sa | NM_002005 | 0.96 |
| *MEF2A* | 0.0002 | 8 | 1 | MADS box transcription enhancer factor 2, polypept | NM_005587 | 0.96 |
| *PLA1A* | 0.0002 | 8 | 1 | phospholipase A1 member A | NM_015900 | 0.96 |
| *POLR2L* | 0.0002 | 8 | 1 | DNA directed RNA polymerase II polypeptide L | NM_021128 | 0.96 |
| *CNN2* | 0.0002 | 26 | 32 | calponin 2 isoform a | NM_004368 NM_201277 | 0.8 |
| *EHD2* | 0.0002 | 10 | 3 | EH-domain containing 2 | NM_014601 | 0.96 |
| *NUMB* | 0.0002 | 10 | 3 | numb homolog isoform 1 | NM_001005743 NM_003744 NM_001005744 NM_001005745 | 0.96 |
| *SPG20* | 0.0002 | 10 | 3 | spartin | NM_015087 | 0.96 |
| *UBAP2* | 0.0002 | 10 | 3 | ubiquitin associated protein 2 | NM_018449 | 0.96 |
| *LOC653949* | 0.0002 | 13 | 7 | similar to 60S ribosomal protein L7 isoform 1 | XM_938789 XM_943868 XM_943872 | 0.96 |
| *PPM1F* | 0.0002 | 9 | 2 | protein phosphatase 1F | NM_014634 | 0.96 |
| *PPWD1* | 0.0002 | 9 | 2 | peptidylprolyl isomerase domain and WD repeat cont | NM_015342 | 0.96 |
| *RALB* | 0.0002 | 9 | 2 | v-ral simian leukemia viral oncogene homolog B | NM_002881 | 0.96 |
| *WWP2* | 0.0002 | 9 | 2 | WW domain containing E3 ubiquitin protein ligase 2 | NM_199424 NM_007014 NM_199423 | 0.96 |
| *RPL13A* | 0.0003 | 87 | 210 | ribosomal protein L13a | NM_012423 | 0 |
| *SEPT7* | 0.0003 | 25 | 31 | cell division cycle 10 isoform 2 | NM_001011553 NM_001788 | 0.79 |
| *OGDH* | 0.0004 | 15 | 11 | oxoglutarate (alpha-ketoglutarate) dehydrogenase ( | NM_001003941 NM_002541 | 0.93 |
| *PTPRF* | 0.0004 | 15 | 11 | protein tyrosine phosphatase, receptor type, F iso | NM_130440 NM_002840 | 0.93 |
| *RPS27* | 0.0004 | 28 | 39 | ribosomal protein S27 | NM_001030 | 0.68 |
| *LRRC41* | 0.0004 | 11 | 5 | MUF1 protein | NM_006369 | 0.95 |
| *CD55* | 0.0005 | 18 | 17 | decay accelerating factor for complement | NM_000574 | 0.88 |
| *KIAA0195* | 0.0006 | 10 | 4 | hypothetical protein LOC9772 | NM_014738 | 0.94 |
| *SNRK* | 0.0006 | 10 | 4 | SNF related kinase | NM_017719 | 0.94 |
| *AP2B1* | 0.0006 | 22 | 26 | adaptor-related protein complex 2, beta 1 subunit | NM_001282 NM_001030006 | 0.8 |
| *MGAT1* | 0.0007 | 12 | 7 | mannosyl (alpha-1,3-)-glycoprotein beta-1,2-N-acet | NM_002406 | 0.93 |
| *TUBB6* | 0.0007 | 12 | 7 | tubulin, beta 6 | NM_032525 | 0.93 |
| *LGALS1* | 0.0007 | 37 | 64 | beta-galactoside-binding lectin precursor lectin | NM_002305 | 0.38 |
| *RPLP1* | 0.0007 | 37 | 64 | ribosomal protein P1 isoform 1 | NM_001003 NM_213725 | 0.38 |
| *HDAC6* | 0.0008 | 9 | 3 | histone deacetylase 6 | NM_006044 | 0.94 |
| *KCTD15* | 0.0008 | 9 | 3 | potassium channel tetramerisation domain containin | NM_024076 | 0.94 |
| *PODXL* | 0.0008 | 9 | 3 | podocalyxin-like precursor isoform 1 podocalyxin-l | NM_001018111 NM_005397 | 0.94 |
| *DRCTNNB1A* | 0.0008 | 7 | 1 | down-regulated by Ctnnb1, a | NM_032581 | 0.93 |
| *FLJ10815* | 0.0008 | 7 | 1 | amino acid transporter | NM_018231 | 0.93 |
| *INTS3* | 0.0008 | 7 | 1 | hypothetical protein LOC65123 | NM_023015 | 0.93 |
| *SLC7A7* | 0.0008 | 7 | 1 | solute carrier family 7 (cationic amino acid trans | NM_003982 | 0.93 |
| *SPHK1* | 0.0008 | 7 | 1 | sphingosine kinase 1 isoform 2 | NM_182965 NM_021972 | 0.93 |
| *TCF8* | 0.0008 | 7 | 1 | transcription factor 8 (represses interleukin 2 ex | NM_030751 | 0.93 |
| *KIAA0690* | 0.0009 | 8 | 2 | hypothetical protein LOC23223 | NM_015179 | 0.94 |
| *PAPSS1* | 0.0009 | 8 | 2 | 3'-phosphoadenosine 5'-phosphosulfate synthase 1 | NM_005443 | 0.94 |
| *PIGT* | 0.0009 | 8 | 2 | phosphatidylinositol glycan, class T precursor pho | NM_015937 | 0.94 |
| *RPLP2* | 0.0009 | 25 | 34 | ribosomal protein P2 | NM_001004 | 0.69 |
| *MED28* | 0.0010 | 11 | 6 | mediator of RNA polymerase II transcription, subun | NM_025205 | 0.92 |
| *BRD2* | 0.0012 | 16 | 15 | bromodomain containing protein 2 | NM_005104 | 0.86 |
| *NFE2L1* | 0.0012 | 16 | 15 | nuclear factor (erythroid-derived 2)-like 1 | NM_003204 | 0.86 |
| *RPL36* | 0.0012 | 16 | 15 | ribosomal protein L36 | NM_015414 NM_033643 | 0.86 |
| *RPL41* | 0.0012 | 27 | 40 | ribosomal protein L41 | NM_001035267 NM_021104 | 0.6 |
| *ITGB1* | 0.0013 | 35 | 61 | integrin beta 1 isoform 1B precursor integrin beta | NM_033666 NM_033667 NM_033668 NM_033669 NM_002211 NM_133376 | 0.37 |
| *RPS14* | 0.0013 | 32 | 53 | ribosomal protein S14 | NM_001025071 NM_001025070 NM_005617 | 0.45 |
| *GSN* | 0.0013 | 12 | 8 | gelsolin isoform b | NM_198252 NM_000177 | 0.9 |
| *KIAA0652* | 0.0013 | 12 | 8 | hypothetical protein LOC9776 | NM_014741 | 0.9 |
| *WDR46* | 0.0013 | 12 | 8 | WD repeat domain 46 | NM_005452 | 0.9 |
| *ECOP* | 0.0014 | 10 | 5 | EGFR-coamplified and overexpressed protein | NM_030796 | 0.92 |
| *IFI16* | 0.0014 | 10 | 5 | interferon, gamma-inducible protein 16 | NM_005531 | 0.92 |
| *LDB2* | 0.0014 | 10 | 5 | LIM domain binding 2 | NM_001290 | 0.92 |
| *MOAP1* | 0.0014 | 10 | 5 | modulator of apoptosis 1 | NM_022151 | 0.92 |
| *NUPL1* | 0.0014 | 10 | 5 | nucleoporin like 1 isoform b | NM_001008564 NM_014089 NM_001008565 | 0.92 |
| *UBN1* | 0.0014 | 10 | 5 | ubinuclein 1 | NM_016936 | 0.92 |
| *P4HB* | 0.0014 | 31 | 51 | prolyl 4-hydroxylase, beta subunit | NM_000918 | 0.46 |
| *PGD* | 0.0016 | 13 | 10 | phosphogluconate dehydrogenase | NM_002631 | 0.89 |
| *SFRS1* | 0.0016 | 13 | 10 | splicing factor, arginine/serine-rich 1 (splicing | NM_006924 | 0.89 |
| *AZIN1* | 0.0017 | 18 | 20 | ornithine decarboxylase antizyme inhibitor | NM_015878 NM_148174 | 0.79 |
| *PRKCSH* | 0.0017 | 18 | 20 | protein kinase C substrate 80K-H isoform 2 | NM_001001329 NM_002743 | 0.79 |
| *RPL30* | 0.0017 | 18 | 20 | ribosomal protein L30 | NM_000989 | 0.79 |
| *RPS25* | 0.0017 | 33 | 57 | ribosomal protein S25 | NM_001028 | 0.38 |
| *FTH1* | 0.0019 | 114 | 318 | ferritin, heavy polypeptide 1 | NM_002032 | 0 |
| *HSPD1* | 0.0020 | 32 | 55 | chaperonin | NM_002156 NM_199440 | 0.39 |
| *GBE1* | 0.0020 | 9 | 4 | glucan (1,4-alpha-), branching enzyme 1 (glycogen | NM_000158 | 0.91 |
| *PGM2* | 0.0020 | 9 | 4 | phosphoglucomutase 2 | NM_018290 | 0.91 |
| *CBARA1* | 0.0020 | 11 | 7 | calcium binding atopy-related autoantigen 1 | NM_006077 | 0.9 |
| *PITRM1* | 0.0020 | 11 | 7 | metalloprotease 1 | NM_014889 | 0.9 |
| *UGDH* | 0.0020 | 11 | 7 | UDP-glucose dehydrogenase | NM_003359 | 0.9 |
| *RPL37* | 0.0023 | 22 | 30 | ribosomal protein L37 | NM_000997 | 0.66 |
| *G3BP* | 0.0025 | 12 | 9 | Ras-GTPase-activating protein SH3-domain-binding p | NM_005754 NM_198395 | 0.87 |
| *PLOD3* | 0.0025 | 12 | 9 | procollagen-lysine, 2-oxoglutarate 5-dioxygenase 3 | NM_001084 | 0.87 |
| *C16orf63* | 0.0027 | 8 | 3 | hypothetical protein LOC123811 | NM_144600 | 0.9 |
| *FAM8A1* | 0.0027 | 8 | 3 | Autosomal Highly Conserved Protein | NM_016255 | 0.9 |
| *LOX* | 0.0027 | 8 | 3 | lysyl oxidase preproprotein | NM_002317 | 0.9 |
| *PCSK7* | 0.0027 | 8 | 3 | proprotein convertase subtilisin/kexin type 7 prep | NM_004716 | 0.9 |
| *TFPI* | 0.0027 | 8 | 3 | tissue factor pathway inhibitor isoform a precurso | NM_006287 NM_001032281 | 0.9 |
| *UBL5* | 0.0027 | 8 | 3 | ubiquitin-like 5 | NM_024292 | 0.9 |
| *DAP* | 0.0028 | 13 | 11 | death-associated protein | NM_004394 | 0.85 |
| *IFI27* | 0.0028 | 13 | 11 | interferon, alpha-inducible protein 27 | NM_005532 | 0.85 |
| *MT2A* | 0.0028 | 16 | 17 | metallothionein 2A | NM_005953 | 0.79 |
| *GSTO1* | 0.0029 | 14 | 13 | glutathione-S-transferase omega 1 | NM_004832 | 0.83 |
| *SEPN1* | 0.0029 | 14 | 13 | selenoprotein N, 1 isoform 1 precursor selenoprote | NM_020451 NM_206926 | 0.83 |
| *XPO6* | 0.0029 | 14 | 13 | exportin 6 | NM_015171 | 0.83 |
| *PPM1G* | 0.0029 | 20 | 26 | protein phosphatase 1G | NM_177983 NM_002707 | 0.69 |
| *ARID1A* | 0.0030 | 10 | 6 | AT rich interactive domain 1A isoform a | NM_006015 NM_139135 | 0.88 |
| *COPG* | 0.0030 | 10 | 6 | coatomer protein complex, subunit gamma 1 | NM_016128 | 0.88 |
| *GTPBP4* | 0.0030 | 10 | 6 | G protein-binding protein CRFG | NM_012341 | 0.88 |
| *BIVM* | 0.0031 | 6 | 1 | basic, immunoglobulin-like variable motif containi | NM_017693 | 0.88 |
| *DDR2* | 0.0031 | 6 | 1 | discoidin domain receptor family, member 2 precurs | NM_006182 NM_001014796 | 0.88 |
| *FNBP1L* | 0.0031 | 6 | 1 | formin binding protein 1-like isoform 2 | NM_017737 NM_001024948 | 0.88 |
| *LOC646195* | 0.0031 | 6 | 1 | similar to 40S ribosomal protein S28 | XM_929151 | 0.88 |
| *OGFOD1* | 0.0031 | 6 | 1 | hypothetical protein LOC55239 | NM_018233 | 0.88 |
| *POLR3H* | 0.0031 | 6 | 1 | polymerase (RNA) III (DNA directed) polypeptide H | NM_138338 NM_001018052 NM_001018050 NM_001018051 | 0.88 |
| *SLC35F2* | 0.0031 | 6 | 1 | solute carrier family 35, member F2 | NM_017515 | 0.88 |
| *STAB1* | 0.0031 | 6 | 1 | stabilin 1 precursor stabilin 1 | NM_015136 | 0.88 |
| *TMEM43* | 0.0031 | 6 | 1 | transmembrane protein 43 | NM_024334 | 0.88 |
| *TTLL5* | 0.0031 | 6 | 1 | tubulin tyrosine ligase-like family, member 5 | NM_015072 | 0.88 |
| *ARAF* | 0.0031 | 7 | 2 | v-raf murine sarcoma 3611 viral oncogene homolog | NM_001654 | 0.89 |
| *C12orf11* | 0.0031 | 7 | 2 | hypothetical protein LOC55726 | NM_018164 | 0.89 |
| *PIGG* | 0.0031 | 7 | 2 | GPI7 protein | NM_017733 | 0.89 |
| *TPP1* | 0.0038 | 11 | 8 | tripeptidyl-peptidase I precursor | NM_000391 | 0.86 |
| *RGS5* | 0.0044 | 12 | 10 | regulator of G-protein signalling 5 | NM_003617 | 0.84 |
| *DLC1* | 0.0046 | 9 | 5 | deleted in liver cancer 1 isoform 1 | NM_182643 NM_006094 NM_024767 | 0.87 |
| *LTA4H* | 0.0046 | 9 | 5 | leukotriene A4 hydrolase leukotriene A-4 hydrolase | NM_000895 | 0.87 |
| *MAP2K3* | 0.0046 | 9 | 5 | mitogen-activated protein kinase kinase 3 isoform | NM_002756 NM_145109 NM_145110 | 0.87 |
| *MCFD2* | 0.0046 | 9 | 5 | multiple coagulation factor deficiency 2 | NM_139279 | 0.87 |
| *EIF2S3* | 0.0046 | 13 | 12 | eukaryotic translation initiation factor 2, subuni | NM_001415 | 0.81 |
| *PSME3* | 0.0046 | 13 | 12 | proteasome activator subunit 3 isoform 1 | NM_005789 NM_176863 | 0.81 |
| *WBP2* | 0.0046 | 19 | 25 | WW domain binding protein 2 | NM_012478 | 0.66 |
| *EWSR1* | 0.0049 | 21 | 30 | Ewing sarcoma breakpoint region 1 isoform EWS-b | NM_013986 NM_005243 | 0.59 |
| *LOC653105* | 0.0059 | 10 | 7 | similar to Proline-rich nuclear receptor coactivat | XM_926359 XM_931209 XM_931214 | 0.84 |
| *METAP2* | 0.0059 | 10 | 7 | methionyl aminopeptidase 2 | NM_006838 | 0.84 |
| *PPIL2* | 0.0059 | 10 | 7 | peptidylprolyl isomerase-like 2 isoform a | NM_014337 NM_148176 NM_148175 | 0.84 |
| *VCP* | 0.0059 | 25 | 41 | valosin-containing protein | NM_007126 | 0.44 |
| *ARHGAP18* | 0.0063 | 8 | 4 | Rho GTPase activating protein 18 | NM_033515 | 0.86 |
| *C14orf78* | 0.0063 | 8 | 4 | AHNAK nucleoprotein 2 isoform 1 | XM_290629 XM_942314 XM_945905 | 0.86 |
| *CDCA8* | 0.0063 | 8 | 4 | cell division cycle associated 8 | NM_018101 | 0.86 |
| *CUL4B* | 0.0063 | 8 | 4 | cullin 4B | NM_003588 | 0.86 |
| *GTF3C5* | 0.0063 | 8 | 4 | general transcription factor IIIC, polypeptide 5, | NM_012087 | 0.86 |
| *LTBR* | 0.0063 | 8 | 4 | lymphotoxin beta receptor | NM_002342 | 0.86 |
| *MBTPS1* | 0.0063 | 8 | 4 | membrane-bound transcription factor site-1 proteas | NM_201268 NM_003791 | 0.86 |
| *RHOBTB1* | 0.0063 | 8 | 4 | Rho-related BTB domain containing 1 | NM_001032380 NM_014836 NM_198225 | 0.86 |
| *SDF4* | 0.0063 | 8 | 4 | calcium binding protein Cab45 precursor | NM_016547 NM_016176 | 0.86 |
| *RPL17* | 0.0063 | 57 | 137 | ribosomal protein L17 | NM_000985 NM_001035006 | 0.01 |
| *MVP* | 0.0064 | 15 | 17 | major vault protein | NM_005115 NM_017458 | 0.73 |
| *UBE1C* | 0.0065 | 11 | 9 | ubiquitin-activating enzyme E1C isoform 3 | NM_198197 NM_198195 NM_003968 | 0.82 |
| *ENC1* | 0.0068 | 14 | 15 | ectodermal-neural cortex (with BTB-like domain) | NM_003633 | 0.75 |
| *GNG12* | 0.0069 | 13 | 13 | G-protein gamma-12 subunit | NM_018841 | 0.77 |
| *MAN2B1* | 0.0069 | 13 | 13 | mannosidase, alpha, class 2B, member 1 precursor | NM_000528 | 0.77 |
| *SND1* | 0.0083 | 21 | 32 | staphylococcal nuclease domain containing 1 | NM_014390 | 0.52 |
| *EGLN2* | 0.0087 | 7 | 3 | EGL nine (C.elegans) homolog 2 isoform 3 | NM_080732 NM_053046 NM_017555 | 0.84 |
| *ELK3* | 0.0087 | 7 | 3 | ELK3 protein | NM_005230 | 0.84 |
| *PARN* | 0.0087 | 7 | 3 | poly(A)-specific ribonuclease (deadenylation nucle | NM_002582 | 0.84 |
| *PPP2R1B* | 0.0087 | 7 | 3 | beta isoform of regulatory subunit A, protein phos | NM_002716 NM_181699 | 0.84 |
| *WWTR1* | 0.0087 | 7 | 3 | WW domain containing transcription regulator 1 | NM_015472 | 0.84 |
| *MYD88* | 0.0087 | 9 | 6 | myeloid differentiation primary response gene (88) | NM_002468 | 0.83 |
| *SCARB2* | 0.0087 | 9 | 6 | scavenger receptor class B, member 2 | NM_005506 | 0.83 |
| *SPTLC1* | 0.0087 | 9 | 6 | serine palmitoyltransferase subunit 1 isoform b | NM_178324 NM_006415 | 0.83 |
| *THRAP4* | 0.0087 | 9 | 6 | thyroid hormone receptor-associated protein 4 | NM_014815 | 0.83 |
| *TSN* | 0.0087 | 9 | 6 | translin | NM_004622 | 0.83 |
| *CAP1* | 0.0087 | 34 | 68 | adenylyl cyclase-associated protein | NM_006367 | 0.17 |
| *PXDN* | 0.0093 | 15 | 18 | peroxidasin homolog | NM_012293 | 0.69 |
| *FAU* | 0.0094 | 18 | 25 | ubiquitin-like protein fubi and ribosomal protein | NM_001997 | 0.6 |
| *RPL5* | 0.0094 | 46 | 105 | ribosomal protein L5 | NM_000969 | 0.03 |
| *SMARCA4* | 0.0095 | 20 | 30 | SWI/SNF-related matrix-associated actin-dependent | NM_003072 | 0.53 |
