# Supplementary material for: A novel method of differential gene expression analysis using multiple cDNA libraries applied to the identification of tumour endothelial genes
Source: BMC Genomics. 2008 Apr 7;9:153. doi: 10.1186/1471-2164-9-153 (PMC2346479; doi:10.1186/1471-2164-9-153)
Supplement: Additional file 9 — SAGE libraries used for experiment 2 were collected from SAGEmap. There were 10 endothelial libraries consisting of 427,254 tags and 11 normal non-endothelial libraries 329,470 tags. [file 1471-2164-9-153-S9.doc]

**Additional File 9:** SAGE libraries used for experiment 2 were collected from SAGEmap. There were 10 endothelial libraries consisting of 427,254 tags and 11 normal non-endothelial libraries 329,470 tags.

| **Endothelial Cell Line Libraries** |
| --- |
| SAGE_Duke_HMVEC(52579 tags) |
| SAGE_Duke_HMVEC+VEGF(58004 tags) |
| Human Glomerular Endothelial Cell(68987 tags) |
| Pulmonary microvascular endothelial cells under shear stress(30615 tags) |
| Human Aortic Endothelial Cell Exposure to 0h Short-Term Chronic Hypoxia (Control)(38446 tags) |
| Human Aortic Endothelial Cell Exposure to 8h Short-Term Chronic Hypoxia (40629 tags) |
| Human Aortic Endothelial Cell Exposure to 24h Short-Term Chronic Hypoxia (42371 tags) |
| Human Pulmonary Artery Endothelial Cell Exposure to 0h Short-Term Chronic Hypoxia (Control)(25706 tags) |
| Human Pulmonary Artery Endothelial Cell Exposure to 8h Short-Term Chronic Hypoxia(27666 tags) |
| Human Pulmonary Artery Endothelial Cell Exposure to 24h Short-Term Chronic Hypoxia(42251 tags) |
|  |
| **Normal Non-Endothelial Cell Line Libraries** |
| SAGE_Duke_post_crisis_fibroblasts(22466 tags) |
| SAGE_Duke_precrisis_fibroblasts(8851 tags) |
| SAGE_H126(32512 tags) |
| SAGE_HMEC-B41(1430 tags) |
| SAGE_HOSE_4(48552 tags) |
| SAGE_HX(32226 tags) |
| SAGE_IOSE29-11(48586 tags) |
| SAGE_NHA(5th)(52261 tags) |
| SAGE_TSU(11377 tags) |
| SAGE_Lung_normal_CL_L16(28983 tags) |
| SAGE_Lung_normal_CL_L15(42226 tags) |
