# Supplementary material for: A novel method of differential gene expression analysis using multiple cDNA libraries applied to the identification of tumour endothelial genes
Source: BMC Genomics. 2008 Apr 7;9:153. doi: 10.1186/1471-2164-9-153 (PMC2346479; doi:10.1186/1471-2164-9-153)
Supplement: Additional file 10 — 27 genes were predicted to be endothelial specific using a combined SAGE and cDNA library analysis of the latest libraries. The genes are sorted in descending order according to the number of non-endothelial library hits. [file 1471-2164-9-153-S10.doc]

**Additional file 10:**

27 genes were predicted to be endothelial specific using a combined SAGE and cDNA library analysis of the latest libraries. The genes are sorted in descending order according to the number of non-endothelial library hits. Experimentally well-characterised endothelial genes are highlighted in bold.

| Unigene ID | Xpfofiler p-value | Nucleotide | cDNA library Result | FDR q-value | Hits in Endo EST pool | Non-Endo pool | Description |
| --- | --- | --- | --- | --- | --- | --- | --- |
| Hs.83169 | 1.00 | NM_002421 | MMP1 | 0.0000 | 203 | 0 | Matrix metallopeptidase 1 |
| Hs.524121 | 0.97 | NM_019055 | **ROBO4** | 0.0000 | 130 | 0 | Roundabout homolog 4 |
| Hs.440848 | 1.00 | NM_000552 | **VWF** | 0.0000 | 73 | 0 | Von Willebrand factor |
| Hs.76206 | 1.00 | NM_001795 | **CDH5** | 0.0000 | 23 | 0 | Cadherin 5 |
| Hs.473819 | 0.96 | NM_182918 | **ERG** | 0.0000 | 16 | 0 | V-ets erythroblastosis virus E26 oncogene |
| Hs.410104 | 0.98 | NM_000020 | ACVRL1 | 0.0001 | 7 | 0 | Activin A receptor type II-like 1 |
| Hs.78224 | 1.00 | NM_002933 | **RNASE1** | 0.0013 | 5 | 0 | RNase A family, 1 |
| Hs.524479 | 0.95 | NM_024756 | **MMRN2** | 0.0059 | 4 | 0 | Multimerin 2 |
| Hs.367639 | 1.00 | NM_017628 | KIAA1546 | - | 0 | 0 | Hypothetical protein |
| Hs.559067 | 0.91 | NM_001029954 | ARMETL1 | - | 0 | 0 | Arginine-rich, mutated in early stage tumors-like 1 |
| Hs.514412 | 1.00 | NM_000442 | **PECAM1** | 0.0000 | 39 | 1 | Platelet/endothelial cell adhesion molecule |
| Hs.172684 | 0.91 | NM_006634 | VAMP5 | 0.1209 | 3 | 1 | Vesicle-associated membrane protein 5 (myobrevin) |
| Hs.466148 | 0.91 | NM_005234 | NR2F6 | 0.6154 | 0 | 1 | Nuclear receptor subfamily 2, group F, member 6 |
| Hs.76753 | 0.91 | NM_000118 | **ENG** | 0.0000 | 149 | 2 | Endoglin (Osler-Rendu-Weber syndrome 1) |
| Hs.431460 | 1.00 | NM_000873 | ICAM2 | 0.0000 | 13 | 2 | Intercellular adhesion molecule 2 |
| Hs.91481 | 1.00 | NM_201446 | EGFL7 | 0.5687 | 0 | 2 | EGF-like-domain, multiple 7 |
| Hs.252180 | 0.94 | NM_003494 | DYSF | 0.0000 | 48 | 3 | Dysferlin, limb girdle muscular dystrophy 2B (autosomal recessive) |
| Hs.511899 | 0.96 | NM_001955 | **EDN1** | 0.0000 | 16 | 3 | Endothelin 1 |
| Hs.567544 | 1.00 | XM_940209 | KIAA0194 | 0.8064 | 1 | 3 | KIAA0194 protein High mobility group domain |
| Hs.418520 | 1.00 | NM_138408 | C6orf51 | 0.9400 | 1 | 4 | Chromosome 6 open reading frame 51 |
| Hs.46446 | 1.00 | NM_005583 | LYL1 | 0.4357 | 0 | 5 | Lymphoblastic leukemia derived sequence 1 |
| Hs.172685 | 1.00 | NM_015024 | XPO7 | 0.9300 | 1 | 5 | Exportin 7 |
| Hs.554776 | 1.00 | NM_004176 | SREBF1 | 0.6154 | 1 | 8 | Sterol regulatory element binding transcription factor 1 |
| Hs.500916 | 1.00 | NM_032727 | INA | 0.2302 | 0 | 11 | Internexin neuronal intermediate filament protein, alpha |
| Hs.76224 | 1.00 | NM_004105 | **EFEMP1** | 0.0000 | 47 | 13 | EGF-containing fibulin-like extracellular matrix protein 1 |
| Hs.517603 | 0.90 | NM_002405 | **MFNG** | 0.5687 | 8 | 23 | Manic fringe homolog (Drosophila) |
| Hs.374477 | 1.00 | NM_005243 | EWSR1 | 0.0049 | 21 | 30 | Ewing sarcoma breakpoint region 1 |
