# Supplementary material for: A novel method of differential gene expression analysis using multiple cDNA libraries applied to the identification of tumour endothelial genes
Source: BMC Genomics. 2008 Apr 7;9:153. doi: 10.1186/1471-2164-9-153 (PMC2346479; doi:10.1186/1471-2164-9-153)
Supplement: Additional file 11 — Experiment 3 included cDNA and SAGE libraries from cancer, microdissected and flow sorted cell lines. 178,653 ESTs and 733,461 SAGE tags were contained within these library pools. [file 1471-2164-9-153-S11.doc]

**Additional file 11:** cDNA libraries: Experiment 3 included cDNA and SAGE libraries from cancer, microdissected and flow sorted cell lines. 178,653 ESTs and 733,461 SAGE tags were contained within these library pools.

Activated T-cells I

Activated T-cells II

Activated T-cells III

Activated T-cells IV

Activated T-cells IX

Activated T-cells V

Activated T-cells VI

Activated T-cells VII

Activated T-cells VIII

Activated T-cells X

Activated T-cells XI

Activated T-cells XII

Activated T-cells XX

CAMA1Ee cell line I

CAMA1Ee cell line II

CCRF-CEM cells, cyclohexamide treated I

cDNA library of activated B cell line 3D5

Chromosome 7 HeLa cDNA Library

Colon carcinoma (Caco-2) cell line I

Colon carcinoma (Caco-2) cell line II

Colon carcinoma (HCC) cell line

Colon carcinoma (HCC) cell line II

HCC cell line (matastasis to liver in mouse)

HCC cell line (matastasis to liver in mouse) II

HeLa cDNA (T. Noma)

HeLa SRIG (Synthetic retinoids induced genes)

Homo sapiens monocyte-derived macrophages

HSC172 cells I

HSC172 cells II

Human 23132 gastric carcinoma cell line

Human breast cancer cell line Bcap 37

Human cell line A431 subclone

Human cell line AGZY-83a

Human cell line PCI-O6A

Human cell line PCI-O6B

Human cell line SK-N-MC

Human cell line TF-1 (D.L.Ma)

Human exocervical cells (CGLee)

Human fibrosarcoma cell line HT1080

Human fibrosarcoma cell line HT1080-6TGc5

Human gastric cancer SGC-7901 cell line

Human GM-CSF-deprived TF-1 cell line (Liu, Hongtao)

Human HeLa (Y. Wang)

Human HeLa cells (M. Lovett)

Human Jurkat cell line mRNA (K. Thiele)

Human K562 erythroleukemic cells

Human lung cancer cell line A549.A549

Human nasopharyngeal carcinoma cell line HNE1

Human neuroblastoma SK-ER3 cells (M. Garnier)

Human newborn melanocytes (T. Vogt)

Human pancreatic cancer cell line Patu 8988t

Human primary melanocytes mRNA (I.M. Eisenbarth)

Human promyelocytic HL60 cell line (S. Herblot)

Human retina cell line ARPE-19

Human salivary gland cell line HSG

Human White blood cells

Jurkat T-cells I

Jurkat T-cells II

Jurkat T-cells III

Jurkat T-cells V

Jurkat T-cells VI

Liver HepG2 cell line

LNCAP cells I

Macrophage I

Macrophage II

Macrophage, subtracted (total cDNA)

MCF7 cell line

Namalwa B cells I

Namalwa B cells II

NCI_CGAP_Br4

NCI_CGAP_Br5

NCI_CGAP_CLL1

NCI_CGAP_GCB0

NCI_CGAP_GCB1

NCI_CGAP_HN1

NCI_CGAP_HN3

NCI_CGAP_HN4

NCI_CGAP_HSC1

NCI_CGAP_Li1

NCI_CGAP_Li2

NCI_CGAP_Ov5

NCI_CGAP_Ov6

NCI_CGAP_Pr1

NCI_CGAP_Pr10

NCI_CGAP_Pr11

NCI_CGAP_Pr16

NCI_CGAP_Pr18

NCI_CGAP_Pr2

NCI_CGAP_Pr20

NCI_CGAP_Pr24

NCI_CGAP_Pr25

NCI_CGAP_Pr3

NCI_CGAP_Pr4

NCI_CGAP_Pr4.1

NCI_CGAP_Pr5

NCI_CGAP_Pr6

NCI_CGAP_Pr7

NCI_CGAP_Pr8

NCI_CGAP_Pr9

Normal human trabecular bone cells

Raji cells, cyclohexamide treated I

Retinal pigment epithelium 0041 cell line

Retinoid treated HeLa cells

Soares melanocyte 2NbHM

Soares_senescent_fibroblasts_NbHSF

Stratagene HeLa cell s3 937216

Supt cells

T, human adult rhabdomyosarcoma cell line

## SAGE libraries

SAGE_HCT116 Colon, cell line derived from colorectal carcinoma

SAGE_Caco_2 Colon, colorectal carcinoma cell line

SAGE_Duke_H392 Brain, Duke glioblastoma multiforme cell line

SAGE_SW837 Colon, cancer cell line

SAGE_RKO Colon, cancer cell line

SAGE_NHA(5th) Brain, normal human astrocyte cells harvested at passage 5

SAGE_ES2-1 Ovarian clear cell carcinoma cell line ES-2, poorly differentiated

SAGE_OVCA432-2 Ovary, carcinoma cell line OVCA432

SAGE_OV1063-3 Ovary, carcinoma cell line OV1063

SAGE_Duke_mhh-1 Brain, c-myc negative medulloblastoma cell line mhh-1

SAGE_Duke-H341 Brain, c-myc positive medulloblastoma cell line H341

SAGE_HOSE_4 Ovary, normal surface epithelium

SAGE_OVP-5 Ovary, pooled cancer cell lines

SAGE_LNCaP Prostate, cell line, androgen dependent

SAGE_HMEC-B41 Cell culture HMEC-B41 of normal human mammary epithelial cells

SAGE_MDA453 Cell line MDA-MB-453 of human breast carcinoma

SAGE_SKBR3 ATCC cell line SK-BR-3, human breast adenocarcinoma

SAGE_A2780-9 Ovary, ovarian cancer cell line A2780

SAGE_Duke_H247_normal Brain, glioblastoma multiforme cell line H247

SAGE_Duke_H247_Hypoxia Brain, Duke glioblastoma multiforme cell line H247, grown under 1.5% oxygen

SAGE_Duke_post_crisis_fibroblasts Skin, postcrisis survival fibroblast cell line

SAGE_Duke_precrisis_fibroblasts Skin, large T antigen transformed human fibroblasts clones

SAGE_A Prostate, cancer cell line, induced with synthetic androgen

SAGE_IOSE29-11 Ovary, surface epithelium line
