# Supplementary material for: A novel method of differential gene expression analysis using multiple cDNA libraries applied to the identification of tumour endothelial genes
Source: BMC Genomics. 2008 Apr 7;9:153. doi: 10.1186/1471-2164-9-153 (PMC2346479; doi:10.1186/1471-2164-9-153)
Supplement: Additional file 12 — 58 endothelial specific genes were predicted by SAGE-CGAP xProfiler. All SAGE and cDNA non-endothelial cell libraries, including those from transformed cell lines and those produced by tissue micro-dissection or cell sorting, were used in this analysis. [file 1471-2164-9-153-S12.doc]

**Additional file 12:**

58 endothelial specific genes were predicted by SAGE-CGAP xProfiler. All SAGE and cDNA non-endothelial cell libraries, including those from transformed cell lines and those produced by tissue micro-dissection or cell sorting, were used in this analysis.

| Unigene | p-value | Gene | q-value | Endo ESTs | Non-Endo | Nucleotide | Description |
| --- | --- | --- | --- | --- | --- | --- | --- |
| Hs.252180 | 1 | DYSF | 0.0000 | 48 | 0 | NM_003494 | Dysferlin, limb girdle muscular dystrophy 2B (autosomal recessive) |
| Hs.495731 | 0.976 | BMX | 0.0000 | 30 | 0 | NM_203281 | BMX non-receptor tyrosine kinase |
| Hs.132314 | 0.972 | ELTD1 | 0.0000 | 29 | 0 | XM_371262 | EGF, latrophilin and seven transmembrane domain containing 1 |
| Hs.268107 | 0.98 | MMRN1 | 0.0000 | 26 | 0 | NM_007351 | Multimerin 1 |
| Hs.473819 | 1 | ERG | 0.0000 | 16 | 0 | NM_182918 | V-ets erythroblastosis virus E26 oncogene like (avian) |
| Hs.78824 | 0.985 | TIE1 | 0.0000 | 16 | 0 | NM_005424 | Tyrosine kinase with immunoglobulin-like and EGF-like domains 1 |
| Hs.391561 | 0.998 | FABP4 | 0.0027 | 4 | 0 | NM_001442 | Fatty acid binding protein 4, adipocyte |
| Hs.524479 | 1 | MMRN2 | 0.0027 | 4 | 0 | NM_024756 | Multimerin 2 |
| Hs.142003 | 0.987 | PEAR1 | 0.0737 | 2 | 0 | XM_371320/XM_938090 | Platelet endothelial aggregation receptor 1 (FLJ00193) |
| Hs.505337 | 0.998 | CLDN5 | 0.2525 | 1 | 0 | NM_003277 | Claudin 5 (transmembrane protein deleted in velocardiofacial syndrome) |
| Hs.525307 | 0.997 | CLEC14A | 0.2525 | 1 | 0 | NM_175060 | C-type lectin domain family 14, member A |
| Hs.233955 | 1 | RASIP1 | 0.2525 | 1 | 0 | NM_017805 | Ras interacting protein 1 |
| Hs.8619 | 0.976 | SOX18 | 0.2525 | 1 | 0 | NM_018419 | SRY (sex determining region Y)-box 18 |
| Hs.559067 | 1 | ARMETL1 | - | 0 | 0 | NM_001029954 | Arginine-rich, mutated in early stage tumors-like 1 |
| Hs.231850 | 0.998 | TUB | - | 0 | 0 | NM_003320/NM_177972 | Tubby homolog (mouse) |
| Hs.91481 | 1 | EGFL7 | - | 0 | 0 | NM_016215/NM_201446 | EGF-like-domain, multiple 7 |
| Hs.2913 | 0.976 | EPHB3 | - | 0 | 0 | NM_004443 | EPH receptor B3 |
| Hs.500916 | 1 | INA | - | 0 | 0 | NM_032727 | Internexin neuronal intermediate filament protein, alpha |
| Hs.524121 | 1 | ROBO4 | 0.0000 | 130 | 1 | NM_019055 | Roundabout homolog 4, magic roundabout (Drosophila) |
| Hs.440848 | 1 | VWF | 0.0000 | 73 | 1 | NM_000552 | Von Willebrand factor |
| Hs.76206 | 1 | CDH5 | 0.0000 | 23 | 1 | NM_001795 | Cadherin 5, type 2, VE-cadherin (vascular epithelium) |
| Hs.97199 | 0.994 | CD93 | 0.0000 | 17 | 1 | NM_012072 | Complement component 1, q subcomponent, receptor 1 |
| Hs.285671 | 0.998 | BMP6 | 0.0049 | 5 | 1 | NM_001718 | Bone morphogenetic protein 6 |
| Hs.479756 | 0.918 | KDR | 0.2525 | 2 | 1 | NM_002253 | Kinase insert domain receptor (a type III receptor tyrosine kinase) |
| Hs.129944 | 0.994 | ESM1 | 0.0000 | 29 | 2 | NM_007036 | Endothelial cell-specific molecule 1 |
| Hs.495728 | 0.967 | PIR | 0.3630 | 2 | 2 | NM_001018109 | Pirin (iron-binding nuclear protein) |
| Hs.511899 | 1 | EDN1 | 0.0000 | 16 | 2 | NM_001955 | Endothelin 1 |
| Hs.97997 | 0.994 | RNASEN | 0.3630 | 2 | 2 | NM_013235 | Ribonuclease III, nuclear (RNASE3L) |
| Hs.410104 | 1 | ACVRL1 | 0.0026 | 7 | 3 | NM_000020 | Activin A receptor type II-like 1 |
| Hs.555874 | 0.999 | HSPG2 | 0.1063 | 4 | 3 | NM_005529 | Heparan sulfate proteoglycan 2 (perlecan) |
| Hs.367639 | 0.999 | KIAA1546 | 0.5682 | 0 | 3 | NM_017628 | Hypothetical protein FLJ20032 |
| Hs.554776 | 1 | SREBF1 | 0.6729 | 1 | 3 | NM_004176 | Sterol regulatory element binding transcription factor 1 |
| Hs.514412 | 1 | PECAM1 | 0.0000 | 39 | 5 | NM_000442 | Platelet/endothelial cell adhesion molecule (CD31 antigen) |
| Hs.78224 | 1 | RNASE1 | 0.1506 | 5 | 6 | NM_198234 | Ribonuclease, RNase A family, 1 (pancreatic) |
| Hs.550502 | 0.908 | LIAS | 0.9701 | 1 | 6 | NM_194451 | Lipoic acid synthetase |
| Hs.418520 | 1 | C6orf51 | 0.9701 | 1 | 6 | NM_138408 | Chromosome 6 open reading frame 51 |
| Hs.32995 | 0.999 | SAP130 | 0.4096 | 0 | 7 | NM_024545 | MSin3A-associated protein 130 |
| Hs.172685 | 1 | XPO7 | 0.8085 | 1 | 8 | NM_015024 | Exportin 7 |
| Hs.76224 | 1 | EFEMP1 | 0.0000 | 47 | 8 | NM_018894 | EGF-containing fibulin-like extracellular matrix protein 1 |
| Hs.46446 | 1 | LYL1 | 0.3388 | 0 | 9 | NM_005583 | Lymphoblastic leukemia derived sequence 1 |
| Hs.475125 | 0.994 | ATXN10 | 0.6111 | 3 | 9 | NM_013236 | Ataxin 10 |
| Hs.466148 | 0.993 | NR2F6 | 0.3106 | 0 | 10 | NM_005234 | Nuclear receptor subfamily 2, group F, member 6 |
| Hs.76753 | 0.993 | ENG | 0.0000 | 149 | 10 | NM_000118 | Endoglin (Osler-Rendu-Weber syndrome 1) |
| Hs.431460 | 1 | ICAM2 | 0.0002 | 13 | 10 | NM_000873 | Intercellular adhesion molecule 2 |
| Hs.292026 | 0.903 | EIF4E2 | 0.6579 | 1 | 10 | NM_004846 | Eukaryotic translation initiation factor 4E member 2 |
| Hs.567544 | 1 | KIAA0194 | 0.6579 | 1 | 11 | XM_940209 | KIAA0194 protein |
| Hs.172684 | 1 | VAMP5 | 0.8091 | 3 | 14 | NM_006634 | Vesicle-associated membrane protein 5 (myobrevin) |
| Hs.83169 | 0.999 | MMP1 | 0.0000 | 203 | 18 | NM_002421 | Matrix metallopeptidase 1 (interstitial collagenase) |
| Hs.374477 | 1 | EWSR1 | 0.0000 | 21 | 25 | NM_013986 | Ewing sarcoma breakpoint region 1 |
| Hs.517603 | 0.991 | MFNG | 0.5682 | 8 | 30 | NM_002405 | Manic fringe homolog (Drosophila) |
| Hs.418241 | 1 | MT2A | 0.0262 | 16 | 32 | NM_005953 | Metallothionein 2A |
| Hs.445351 | 0.986 | LGALS1 | 0.0001 | 37 | 74 | NM_002305 | Lectin, galactoside-binding, soluble, 1 (galectin 1) |
| Hs.565318/Hs.262886 | 0.98 | INPP5D | 0.0008 | 1 | 78 | NM_005541 | Inositol polyphosphate-5-phosphatase, 145kDa (INPP5D)Transcribed locus |
| Hs.414795 | 1 | SERPINE1 | 0.0000 | 97 | 94 | NM_000602 | Serpin peptidase inhibitor, clade E (nexin, plasminogen activator inhibitor type 1) |
| Hs.522584 | 0.903 | TMSB4X | 0.0000 | 120 | 162 | NM_021109 | Thymosin, beta 4, X-linked |
| Hs.49265 | 0.994 | - |  |  |  |  | Homo sapiens, clone IMAGE:4690669, mRNA |
| Hs.483538 | 0.998 | - |  |  |  |  | Transcribed locus, moderately similar to XP_429401 |
| Hs.170508 | 0.967 | - |  |  |  |  | Transcribed locus |
