# Supplementary material for: A novel method of differential gene expression analysis using multiple cDNA libraries applied to the identification of tumour endothelial genes
Source: BMC Genomics. 2008 Apr 7;9:153. doi: 10.1186/1471-2164-9-153 (PMC2346479; doi:10.1186/1471-2164-9-153)
Supplement: Additional file 13 — By combining all the genes found from experiments 1, 2 and 3, a non-redundant comprehensive list of 459 endothelial genes is produced. HUGO and Refseq interim gene symbols are presented. [file 1471-2164-9-153-S13.doc]

**Additional File 13:** By combining all the genes found from experiments 1, 2 and 3, a non-redundant comprehensive list of 459 endothelial genes is produced. HUGO and Refseq interim gene symbols are presented.

| *EDN1* | *VAMP5* | *ITGA5* | *FNBP1L* | *WDR46* | *SLC39A9* | *SNX12* | *RPL31* | *PPIA* | C9orf26 | *PLS3* | *WDR6* | *LTBR* | *MMS19L* |
| --- | --- | --- | --- | --- | --- | --- | --- | --- | --- | --- | --- | --- | --- |
| *ENG* | *XPO7* | *RNU2* | *MAP3K8* | *XPO6* | *PITRM1* | *SDF4* | *SURF4* | *UBE3C* | *PPIL2* | *RPL30* | *RALB* | *CTGF* | *LOC653352* |
| *EWSR1* | *LYL1* | *AZIN1* | *GANAB* | *NRP1* | *UGDH* | *TPP1* | *FLJ22746* | *CASP7* | *RGS4* | *APLP2* | *SNRK* | *TCF4* | *CBARA1* |
| *PECAM1* | *NR2F6* | *EMCN* | *LOX* | *RPS7* | *A2M* | *BIVM* | *ABI1* | *PALMD* | *S100A6* | *PLOD3* | *PCSK7* | *COPG* | *DNAJC10* |
| *DYSF* | *EGFL7* | *SLC7A7* | *SPTLC1* | *CEP135* | *MYCT1* | *PTPRF* | *EDG1* | *TMEM43* | *LDB2* | *EFTUD2* | *CYR61* | *FAM43A* | *TNFRSF1A* |
| *ICAM2* | *EIF4E2* | *PRKCSH* | *PCDH12* | *VIM* | *PHF8* | *ENC1* | *HSPD1* | *TXNDC5* | *VAT1* | *JAM2* | *KDELC1* | *NUPL1* | *ARHGEF15* |
| *EFEMP1* | *KDR* | *LRRC41* | *NDUFS1* | *WARS* | *RPS20* | *RPS27* | *RPS25* | *RPS12* | *PMM2* | *S100A8* | *TM6SF1* | *C16orf30* | *SMARCA4* |
| *RNASE1* | *SAP130* | *RPL26* | *PARN* | *ZNF586* | *SDPR* | *LIFR* | *STAB1* | *SEC14L1* | *UBA52* | *DYNC1I2* | *SPG20* | *MED28* | *DRCTNNB1A* |
| *ERG* | *PIR* | *SOX7* | *GSN* | *FAM8A1* | *SDCBP* | *TYK2* | *MCAM* | *ARAF* | *TTLL5* | *C7* | *KIAA0690* | *GPR56* | *LOC650049* |
| *MMRN2* | *CLDN5* | *PTTG1IP* | *WWTR1* | *EXOC6* | *SPHK1* | *NQO1* | *SPARC* | *PLOD1* | *G3BP* | *XLKD1* | *RPL37* | *METAP2* | *KIAA0652* |
| *ACVRL1* | *ATXN10* | *RPS18* | *MYD88* | *GJA1* | *CCL20* | *NDRG1* | *RAD54L2* | *PXDN* | *STARD4* | *CS* | *PPP2R1B* | *SGK* | *LOC646195* |
| *VWF* | *LIAS* | *POLR2L* | *TMBIM1* | *NONO* | *TEK* | *SNAP23* | *DDR2* | *PPP2R1A* | *SND1* | *RPLP2* | *VAMP3* | *NUMB* | *SERPINH1* |
| *ROBO4* | *CLEC14A* | *TMSB10* | *C16orf63* | *LENG4* | *FAM62A* | *RPL5* | *WWP2* | *SLC35A2* | *KIAA0195* | *RPS24* | *PIGT* | *SLC29A1* | *MAP1LC3B* |
| *CDH5* | *RNASEN* | *FES* | *GNAI3* | *ELOVL5* | *RPS26* | *TNPO1* | *CALU* | *MBTPS1* | *CALCRL* | *SPARCL1* | *SLC35A5* | *WASF2* | *ARHGAP18* |
| *MMP1* | *TUB* | *CNN2* | *HHIP* | *UBN1* | *CLIC4* | *GBE1* | *ZNF207* | *PSME3* | *TFPI2* | *NFE2L1* | *GBP4* | *FLJ39531* | *ARHGAP24* |
| *SERPINE1* | *EPHB3* | *NUP54* | *TRAM2* | *SPRR3* | *DLC1* | *CDC42* | *HYOU1* | *TCF8* | *RPS27A* | *UNC45A* | *PAPSS1* | *PPWD1* | *LOC653949* |
| *LGALS1* | *PEAR1* | *RGS5* | *RPL13A* | *SHC1* | *PLA2G4C* | *GTPBP4* | *TAF15* | *WSB1* | *F2R* | *RPAP1* | *IKBKE* | *TMEM154* | *LOC152485* |
| *TIE1* | *RASIP1* | *PLA1A* | *DAB2* | *VEPH1* | *PPM1F* | *ACTA1* | *THBD* | *XRCC6* | *GPR177* | *ACP2* | *PI4K2B* | *MVP* |  |
| *TMSB4X* | *HSPG2* | *TP53* | *C14orf78* | *TFPI* | *MCFD2* | *UACA* | *MGAT1* | *DYNLL1* | *CAV1* | *MAN2B1* | *KIAA1539* | *RPS8* |  |
| *MT2A* | *SOX18* | *FTH1* | *TPD52L2* | *GSTO1* | *LRRC8C* | *GPR4* | *LAMA4* | *ELK3* | *THBS1* | *MAP2K3* | *HSPA8* | *HEY1* |  |
| *ESM1* | *INPP5D* | *CUL4B* | *RPL36* | *RPL37A* | *PPM1G* | *RPS15A* | *TNFSF18* | *HEL308* | *NUCB1* | *PGD* | *GRN* | *FGD5* |  |
| *BMP6* | *EMP1* | *CDCA8* | *NOSTRIN* | *QSER1* | *CD55* | *HEXB* | *MOAP1* | *PTRF* | *ALDH1A1* | *EXOSC10* | *KIAA0174* | *RGS1* |  |
| *FABP4* | *FSTL1* | *FAU* | *RPL41* | *PRG1* | *OGDH* | *BAG3* | *SEPT7* | *OS9* | *NM_001042465* | *RPL24* | *LOC650626* | *SELE* |  |
| *ELTD1* | *HNRPH1* | *RNF40* | *RPS15* | *DUSP6* | *POSTN* | *THRAP4* | *EIF4G2* | *ENPP2* | *LOC653105* | *RHOBTB1* | *C4orf18* | *SMURF2* |  |
| *BMX* | *FLJ10815* | *C12orf11* | *GTF3C5* | *FNTB* | *SHQ1* | *P4HB* | *TUBB6* | *MEF2A* | *RAPGEF3* | *PRSS23* | *RPL27A* | *RHOJ* |  |
| *CD93* | *ECE1* | *RPL19* | *LDLR* | *NRCAM* | *MOV10L1* | *FLNB* | *PODXL* | *FZD4* | *GTF3C4* | *VCP* | *ANTXR2* | *INTS3* |  |
| *MMRN1* | *MMP2* | *EIF2S3* | *KCTD15* | *SERP1* | *SFRS1* | *IFI27* | *RPL17* | *UBL5* | *MUS81* | *BGN* | *ZNF521* | *HDAC6* |  |
| *KIAA0194* | *ANXA2* | *GRWD1* | *ITGB1* | *ATP5H* | *RANBP1* | *UBE1C* | *EVI1* | *GPI* | *SCARB2* | *SEPN1* | *MAT2A* | *LTA4H* |  |
| *INA* | *IFI16* | *MOV10* | *BRD2* | *RHOBTB3* | *MANSC1* | *SRPX2* | *RHOB* | *CCDC99* | *RPS14* | *WBP2* | *RPS13* | *UBC* |  |
| *KIAA1546* | *ACTR3* | *DCTN1* | *PCDH1* | *SLC35F2* | *CAP1* | *AP2B1* | *RALA* | *C10orf10* | *ART4* | *RPL32* | *TSN* | *HDLBP* |  |
| *C6orf51* | *CSTA* | *NOD27* | *FBXL3* | *DAP* | *POLR3H* | *MYO1C* | *PCBP4* | *TNNC2* | *ADAM15* | *GNG12* | *EGLN2* | *SRPX* |  |
| *SREBF1* | *PGM2* | *SMG6* | *PLD2* | *TGM2* | *CNP* | *WDR1* | *UBAP2* | *GIMAP6* | *MAPRE1* | *NCL* | *ARID1A* | *ECSM1* |  |
| *MFNG* | *PRCP* | *DDX5* | *RPN1* | *SCAMP4* | *SPINK5* | *ADSS* | *RPLP1* | *ZNF346* | *TXNRD1* | *ECOP* | *TARDBP* | *ANGPT2* |  |
| *ARMETL1* | *CD34* | *SERPINB5* | *SERINC3* | *PIGG* | *EHD2* | *RNF4* | *LMNA* | *TINAGL1* | *ECSM2* | *APLN* | *IL1RL1* | *OGFOD1* |  |
