# Supplementary material for: A novel method of differential gene expression analysis using multiple cDNA libraries applied to the identification of tumour endothelial genes
Source: BMC Genomics. 2008 Apr 7;9:153. doi: 10.1186/1471-2164-9-153 (PMC2346479; doi:10.1186/1471-2164-9-153)
Supplement: Additional file 14 — 237 Brain tumour bulk tissue libraries containing 140,621 ESTs were used versus brain normal libraries to find differentially expressed genes. [file 1471-2164-9-153-S14.doc]

**Additional file 14:** 237 Brain tumour bulk tissue libraries containing 140,621 ESTs were used versus brain normal libraries to find differentially expressed genes.

Human Glialblastoma Cell

NCI_CGAP_Brn20

NCI_CGAP_Brn21

NCI_CGAP_Brn23

NCI_CGAP_Brn25

NCI_CGAP_Brn35

NCI_CGAP_Brn41

NCI_CGAP_Brn50

NCI_CGAP_Brn52

NCI_CGAP_Brn53

NCI_CGAP_Brn64

NCI_CGAP_Brn65

NCI_CGAP_Brn66

NCI_CGAP_Brn67

NCI_CGAP_Brn70

NIH_MGC_192

NT0001

NT0002

NT0003

NT0004

NT0005

NT0006

NT0007

NT0008

NT0010

NT0011

NT0012

NT0013

NT0014

NT0015

NT0018

NT0020

NT0022

NT0023

NT0024

NT0025

NT0027

NT0028

NT0029

NT0031

NT0032

NT0033

NT0035

NT0036

NT0037

NT0038

NT0039

NT0040

NT0042

NT0043

NT0045

NT0046

NT0048

NT0050

NT0052

NT0053

NT0054

NT0057

NT0058

NT0071

NT0072

NT0073

NT0074

NT0075

NT0076

NT0077

NT0078

NT0079

NT0080

NT0081

NT0082

NT0083

NT0084

NT0086

NT0087

NT0088

NT0089

NT0090

NT0092

NT0098

NT0099

NT0100

NT0101

NT0102

NT0103

NT0104

NT0105

NT0109

NT0110

NT0112

NT0113

NT0115

NT0116

NT0117

**Additional file 14:** Brain tumour bulk libraries

NT0118

NT0119

NT0120

NT0121

NT0122

NT0123

NT0124

NT0125

NT0126

NT0128

NT0129

NT0131

NT0132

NT0133

NT0135

NT0136

NT0137

NT0138

NT0139

NT0140

NT0141

NT0142

NT0143

NT0144

NT0146

NT0147

NT0148

NT0149

NT0150

NT0151

NT0152

NT0153

NT0154

NT0155

NT0156

NT0157

NT0158

NT0159

NT0162

NT0163

NT0164

NT0167

NT0168

NT0169

NT0170

NT0171

NT0175

NT0176

NT0177

NT0178

NT0179

NT0180

NT0181

NT0182

NT0185

NT0186

NT0187

NT0189

NT0192

NT0193

NT0194

NT0196

NT0197

NT0198

NT0199

NT0200

NT0201

NT0202

NT0203

NT0204

NT0209

NT0210

NT0211

NT0213

NT0214

NT0215

NT0216

NT0217

NT0219

NT0221

NT0224

NT0225

NT0226

NT0227

NT0228

NT0229

NT0230

NT0232

**Additional file 14:** Brain tumour bulk libraries

NT0233

NT0235

NT0236

NT0237

NT0239

NT0242

NT0243

NT0244

NT0245

NT0246

NT0247

NT0248

NT0249

NT0250

NT0251

NT0252

NT0256

NT0257

NT0261

NT0263

NT0264

NT0265

NT0266

NT0267

NT0268

NT0269

NT0270

NT0271

NT0272

NT0273

NT0274

NT0278

NT0280

NT0282

NT0283

NT0284

NT0285

NT0286

NT0287

NT0288

NT0289

NT0290

NT0291

NT0294

NT0299

NT0312

NT0314

NT0319

NT0837

NT1006

Schiller astrocytoma

Schiller meningioma

Schiller oligodendroglioma

human favorable neuroblastoma

human unfavorable neuroblastoma
