# Supplementary material for: A novel method of differential gene expression analysis using multiple cDNA libraries applied to the identification of tumour endothelial genes
Source: BMC Genomics. 2008 Apr 7;9:153. doi: 10.1186/1471-2164-9-153 (PMC2346479; doi:10.1186/1471-2164-9-153)
Supplement: Additional file 15 — 24 Brain foetal bulk tissue libraries containing 69,862 ESTs were used versus brain normal libraries to find differentially expressed genes. [file 1471-2164-9-153-S15.doc]

**Additional File 15:** 24 Brain foetal bulk tissue libraries containing 69,862 ESTs were used versus brain normal libraries to find differentially expressed genes.

547 (synonym: hfbr1)

564 (synonym: hfbr2)

Brain IV brain

Chromosome 7 Fetal Brain cDNA Library

ClonTech HL 1065a

Clontech human fetal brain polyA+ mRNA (#6535)

FHTA

FHTB

Fetal Brain, Bento Soares

Fetal brain I

Fetal brain III

Fetal brain library

Fetal brain, Stratagene

Fetal brain, Stratagene (cat#936206)

Homo sapiens FETAL BRAIN

Human fetal brain (TFujiwara)

Human fetal brain QBoqin2

Human fetal brain S. Meier-Ewert

LTI_FL013_FBrn1

NIH_MGC_121

P, Human foetal Brain Whole tissue

Schneider fetal brain 00004

hEx1 (RZPD no. 800)

GeneTrack, 4p16.3 JM Rommen
