# Supplementary material for: A novel method of differential gene expression analysis using multiple cDNA libraries applied to the identification of tumour endothelial genes
Source: BMC Genomics. 2008 Apr 7;9:153. doi: 10.1186/1471-2164-9-153 (PMC2346479; doi:10.1186/1471-2164-9-153)
Supplement: Additional file 16 — 302 Brain normal bulk tissue libraries containing 100,554 ESTs were used versus brain tumour/foetal libraries to find differentially expressed genes. [file 1471-2164-9-153-S16.doc]

**Additional file 16:** 302 Brain normal bulk tissue libraries containing 100,554 ESTs were used versus brain tumour/foetal libraries to find genes.

NIH_MGC_114

NIH_MGC_119

761 (synonym: hamy2)

Corpus callosum II

DKFZphamy1

DKFZphsnu1

HTB

HTC

HTE

HTF

Homo sapiens ADULT BRAIN

Cerebellum II

Hippocampus I

Hippocampus II

Johnston frontal cortex

NN0001

NN0003

NN0004

NN0005

NN0006

NN0007

NN0008

NN0009

NN0010

NN0011

NN0012

NN0021

NN0023

NN0024

NN0025

NN0026

NN0027

NN0028

NN0030

NN0031

NN0032

NN0033

NN0034

NN0035

NN0036

NN0037

NN0038

NN0039

NN0040

NN0041

NN0042

NN0044

NN0045

NN0046

NN0047

NN0048

NN0049

NN0050

NN0051

NN0052

NN0053

NN0054

NN0055

NN0056

NN0057

NN0058

NN0059

NN0060

NN0061

NN0062

NN0063

NN0064

NN0066

NN0067

NN0068

NN0070

NN0071

NN0072

NN0073

NN0074

NN0075

NN0076

NN0078

NN0079

NN0080

NN0081

NN0082

NN0083

NN0084

NN0087

NN0088

NN0089

NN0090

NN0091

NN0092

NN0098

NN0099

NN0101

NN0104

NN0107

NN0110

NN0116

NN0117

NN0118

NN0119

NN0120

NN0127

NN0128

NN0129

NN0130

NN0131

NN0133

NN0134

NN0135

NN0136

NN0141

NN0142

NN0143

NN0144

NN0145

NN0146

NN0155

NN0157

NN0165

NN0166

NN0167

NN0168

NN0169

NN0170

NN0171

NN0172

NN0173

NN0174

NN0177

NN0178

NN0179

NN0182

NN0183

NN0184

NN0186

NN0187

NN0188

NN0189

NN0190

NN0191

**Additional file 16:** Brain normal libraries

NN0193

NN0194

NN0195

NN0196

NN0198

NN0199

NN0200

NN0203

NN0204

NN0205

NN0206

NN0208

NN0209

NN0210

NN0211

NN0212

NN0213

NN0214

NN0215

NN0216

NN0217

NN0218

NN0219

NN0220

NN0221

NN0222

NN0223

NN0225

NN0226

NN0227

NN0228

NN0229

NN0230

NN0231

NN0232

NN0233

NN0234

NN0237

NN0238

NN0243

NN0244

NN0245

NN0246

NN0247

NN0248

NN0249

NN0250

NN0251

NN0252

NN0253

NN0255

NN0256

NN0257

NN0258

NN0259

NN0261

NN0262

NN0267

NN0268

NN0273

NN0281

NN1002

NN1003

NN1004

NN1005

NN1006

NN1008

NN1009

NN1010

NN1011

NN1012

NN1013

NN1014

NN1015

NN1018

NN1020

NN1021

NN1022

NN1023

NN1024

NN1025

NN1026

NN1027

NN1028

NN1029

NN1030

NN1031

NN1032

NN1035

NN1037

NN1038

NN1039

NN1040

NN1041

NN1042

NN1043

NN1044

NN1045

NN1046

NN1047

NN1048

NN1049

NN1050

NN1055

NN1059

NN1060

NN1061

NN1062

NN1063

NN1064

NN1065

NN1066

NN1067

NN1068

NN1071

NN1072

NN1073

NN1075

NN1076

NN1077

NN1078

NN1080

NN1081

NN1082

NN1083

NN1084

NN1085

NN1086

NN1089

NN1101

NN1104

NN1105

NN1111

NN1112

NN1120

NN1121

NN1122

NN1123

NN1124

NN1125

NN1126

**Additional file 16:** Brain normal libraries

NN1128

NN1129

NN1130

NN1131

NN1140

NN1141

NN1142

NN1145

NN1146

NN1147

NN1148

NN1149

NN1152

NN1153

NN1154

NN1155

NN1156

NN1157

NN1158

NN1161

NN1162
