# Supplementary material for: A novel method of differential gene expression analysis using multiple cDNA libraries applied to the identification of tumour endothelial genes
Source: BMC Genomics. 2008 Apr 7;9:153. doi: 10.1186/1471-2164-9-153 (PMC2346479; doi:10.1186/1471-2164-9-153)
Supplement: Additional file 17 — 178 lung tumour bulk tissue libraries containing 108,107 ESTs were used versus lung normal libraries to find differentially expressed genes. [file 1471-2164-9-153-S17.doc]

**Additional file 17:** 178 lung tumour bulk tissue libraries containing 108,107 ESTs were used versus lung normal libraries to find differentially expressed genes.

ET0001

ET0005

ET0008

ET0009

ET0011

ET0014

ET0015

ET0016

ET0017

ET0018

ET0019

ET0020

ET0021

ET0022

ET0023

ET0024

ET0026

ET0027

ET0028

ET0029

ET0030

ET0031

ET0037

ET0038

ET0039

ET0040

ET0041

ET0042

ET0043

ET0044

ET0134

ET0135

ET0136

ET0138

ET0139

ET0134

ET0135

ET0136

ET0138

ET0147

ET0148

ET0149

ET0150

ET0151

ET0152

ET0153

ET0154

ET0155

ET0163

ET0164

ET0171

ET0172

ET0139

ET0140

ET0141

ET0142

ET0144

ET0145

ET0146

ET0140

ET0141

ET0142

ET0144

ET0145

ET0146

ET0147

ET0148

ET0045

ET0046

ET0047

ET0058

ET0060

ET0061

ET0063

ET0066

ET0067

ET0068

ET0070

ET0072

ET0073

ET0074

ET0078

ET0079

ET0080

ET0081

ET0082

ET0084

ET0093

ET0094

ET0095

ET0096

ET0097

ET0098

ET0099

ET0100

ET0101

ET0102

ET0103

ET0104

ET0105

ET0106

ET0107

ET0108

ET0109

ET0110

ET0111

ET0112

ET0113

ET0114

ET0115

ET0116

ET0117

ET0118

ET0119

ET0120

ET0121

ET0122

ET0123

ET0124

ET0125

ET0126

ET0127

ET0129

ET0130

ET0131

ET0132

ET0133

ET0173

ET0174

ET0175

ET0176

ET0177

ET0178

ET0180

ET0181

ET0183

ET0184

ET0185

**Additional file 17:** Lung tumour libraries

ET0188

ET0191

ET0192

ET0193

ET0195

ET0196

ET0197

ET0198

ET0199

ET0200

ET0201

ET0202

ET0203

ET0205

ET0206

ET0207

ET0208

ET0209

ET0211

ET0226

ET0227

ET0237

ET0245

ET0251

ET0254

ET0255

ET0256

ET0257

ET0258

ET0259

ET0260

ET0261

ET0262

ET0266

ET0267

ET0268

ET0272

ET0273

ET0274

ET0275

ET0277

ET0278

ET0675

Lung tumor II

NCI_CGAP_Lu1

NCI_CGAP_Lu19

NCI_CGAP_Lu21

NCI_CGAP_Lu24

NCI_CGAP_Lu27

NCI_CGAP_Lu28

NCI_CGAP_Lu5

NCI_CGAP_Lu6

NCI_CGAP_Lu7
