# Supplementary material for: A novel method of differential gene expression analysis using multiple cDNA libraries applied to the identification of tumour endothelial genes
Source: BMC Genomics. 2008 Apr 7;9:153. doi: 10.1186/1471-2164-9-153 (PMC2346479; doi:10.1186/1471-2164-9-153)
Supplement: Additional file 18 — 10 lung foetal bulk tissue libraries containing 112,690 ESTs were used versus lung normal libraries to find differentially expressed genes. [file 1471-2164-9-153-S18.doc]

**Additional file 18:** 10 lung foetal bulk tissue libraries containing 112,690 ESTs were used versus lung normal libraries to find differentially expressed genes.

Fetal lung I

Fetal lung II

Fetal lung III

HSC172 cells II

Human fetal lung

Soares_fetal_lung_NbHL19W

UI-CF-EC0

UI-CF-EC1

NIH_MGC_122 pooled tissue

Soares_NFL_T_GBC_S1
