# Supplementary material for: A novel method of differential gene expression analysis using multiple cDNA libraries applied to the identification of tumour endothelial genes
Source: BMC Genomics. 2008 Apr 7;9:153. doi: 10.1186/1471-2164-9-153 (PMC2346479; doi:10.1186/1471-2164-9-153)
Supplement: Additional file 19 — 91 lung normal bulk tissue libraries containing 82,757 ESTs were used versus lung tumour/foetal libraries to find differentially expressed genes. [file 1471-2164-9-153-S19.doc]

**Additional file 19:** 91 lung normal bulk tissue libraries containing 82,757 ESTs were used versus lung tumour/foetal libraries to find differentially expressed genes.

EN0001

EN0002

EN0003

EN0004

EN0005

EN0006

EN0007

EN0008

EN0009

EN0010

EN0011

EN0012

EN0013

EN0014

EN0018

EN0020

EN0022

EN0023

EN0024

EN0025

EN0026

EN0027

EN0029

EN0030

EN0031

EN0032

EN0040

EN0042

EN0045

EN0046

EN0047

EN0048

EN0049

EN0051

EN0052

EN0053

EN0054

EN0055

EN0058

EN0059

EN0060

EN0061

EN0063

EN0064

EN0065

EN0066

EN0067

EN0068

EN0071

EN0072

EN0073

EN0074

EN0075

EN0077

EN0078

EN0079

EN0080

EN0081

EN0083

EN0084

EN0085

EN0086

EN0087

EN0088

EN0089

EN0090

EN0091

EN0092

EN0093

EN0094

EN0095

EN0096

EN0097

EN0098

EN0100

EN0101

EN0102

EN0109

EN0111

EN0112

EN0132

EN0133

EN0194

EN0220

Human Lung

Lung

NIH_MGC_77

Stratagene lung (#937210)

UI-CF-DU0

UI-CF-DU1

UI-CF-FN0
