# Supplementary material for: A novel method of differential gene expression analysis using multiple cDNA libraries applied to the identification of tumour endothelial genes
Source: BMC Genomics. 2008 Apr 7;9:153. doi: 10.1186/1471-2164-9-153 (PMC2346479; doi:10.1186/1471-2164-9-153)
Supplement: Additional file 20 — 7 kidney bulk tumour tissue libraries containing 38,519 ESTs were used versus kidney normal libraries to find differentially expressed genes. [file 1471-2164-9-153-S20.doc]

**Additional file 20:** 7 kidney bulk tumour tissue libraries containing 38,519 ESTs were used versus kidney normal libraries to find differentially expressed genes.

NCI_CGAP_Kid1

NCI_CGAP_Kid12

NCI_CGAP_Kid13

NCI_CGAP_Kid5

NCI_CGAP_Kid6

NCI_CGAP_Kid7

NCI_CGAP_Kid8
