# Supplementary material for: A novel method of differential gene expression analysis using multiple cDNA libraries applied to the identification of tumour endothelial genes
Source: BMC Genomics. 2008 Apr 7;9:153. doi: 10.1186/1471-2164-9-153 (PMC2346479; doi:10.1186/1471-2164-9-153)
Supplement: Additional file 21 — 5 kidney bulk foetal tissue libraries containing 2,605 ESTs were used versus kidney normal libraries to find differentially expressed genes. [file 1471-2164-9-153-S21.doc]

**Additional file 21:** 5 kidney bulk foetal tissue libraries containing 2,605 ESTs were used versus kidney normal libraries to find differentially expressed genes.

566 (synonym: hfkd2)

Fetal kidney I

Fetal kidney II

Fetal kidney III

NEM subtracted human fetal kidney cDNA
