# Supplementary material for: A novel method of differential gene expression analysis using multiple cDNA libraries applied to the identification of tumour endothelial genes
Source: BMC Genomics. 2008 Apr 7;9:153. doi: 10.1186/1471-2164-9-153 (PMC2346479; doi:10.1186/1471-2164-9-153)
Supplement: Additional file 22 — 5 kidney bulk normal tissue libraries containing 72,476 ESTs were used versus kidney tumour/foetal libraries to find differentially expressed genes. [file 1471-2164-9-153-S22.doc]

**Additional file 22:** 5 kidney bulk normal tissue libraries containing 72,476 ESTs were used versus kidney tumour/foetal libraries to find differentially expressed genes.

Human Kidney

Kidney I

NCI_CGAP_Kid11

NCI_CGAP_Kid3

NIH_MGC_75
