# Supplementary material for: A novel method of differential gene expression analysis using multiple cDNA libraries applied to the identification of tumour endothelial genes
Source: BMC Genomics. 2008 Apr 7;9:153. doi: 10.1186/1471-2164-9-153 (PMC2346479; doi:10.1186/1471-2164-9-153)
Supplement: Additional file 23 — 131 prostate bulk tumour tissue libraries containing 19,125 ESTs were used versus prostate normal libraries to find differentially expressed genes. [file 1471-2164-9-153-S23.doc]

**Additional file 23:** 131 prostate bulk tumour tissue libraries containing 19,125 ESTs were used versus prostate normal libraries to find differentially expressed genes.

FT0001

FT0002

FT0004

FT0005

FT0006

FT0010

FT0014

FT0015

FT0018

FT0019

FT0020

FT0021

FT0022

FT0023

FT0024

FT0025

FT0027

FT0028

FT0029

FT0032

FT0037

FT0038

FT0039

FT0040

FT0041

FT0042

FT0044

FT0045

FT0046

FT0049

FT0050

FT0051

FT0052

FT0053

FT0054

FT0055

FT0056

FT0057

FT0058

FT0059

FT0060

FT0061

FT0062

FT0067

FT0068

FT0069

FT0070

FT0071

FT0073

FT0074

FT0077

FT0079

FT0080

FT0082

FT0083

FT0089

FT0093

FT0095

FT0096

FT0097

FT0099

FT0100

FT0101

FT0103

FT0104

FT0111

FT0115

FT0116

FT0117

FT0119

FT0122

FT0123

FT0126

FT0128

FT0129

FT0130

FT0131

FT0133

FT0134

FT0144

FT0145

FT0148

FT0149

FT0150

FT0151

FT0152

FT0153

FT0157

FT0159

FT0160

FT0161

FT0162

FT0163

FT0164

FT0166

FT0167

FT0169

FT0170

FT0175

FT0176

FT0179

FT0180

FT0181

FT0182

FT0183

FT0184

FT0186

FT0190

FT0191

FT0192

FT0193

FT0194

FT0195

FT0199

FT0200

FT0201

FT0202

FT0203

FT0204

FT0205

FT0206

FT0207

FT0208

FT0209

FT0210

FT0211

FT0212

NCI_CGAP_Pr23

bvtumor

tumor1

tumor2
