# Supplementary material for: A novel method of differential gene expression analysis using multiple cDNA libraries applied to the identification of tumour endothelial genes
Source: BMC Genomics. 2008 Apr 7;9:153. doi: 10.1186/1471-2164-9-153 (PMC2346479; doi:10.1186/1471-2164-9-153)
Supplement: Additional file 24 — 129 prostate bulk normal tissue libraries containing 68,480 ESTs were used versus prostate tumour libraries to find differentially expressed genes. [file 1471-2164-9-153-S24.doc]

**Additional file 24:** 129 prostate bulk normal tissue libraries containing 68,480 ESTs were used versus prostate tumour libraries to find genes.

Barstead prostate BPH HPLRB4 1

FN0004

FN0005

FN0006

FN0007

FN0009

FN0010

FN0011

FN0012

FN0019

FN0020

FN0021

FN0022

FN0023

FN0025

FN0026

FN0027

FN0028

FN0037

FN0039

FN0040

FN0041

FN0042

FN0043

FN0044

FN0046

FN0047

FN0048

FN0050

FN0053

FN0055

FN0056

FN0057

FN0058

FN0059

FN0060

FN0061

FN0063

FN0064

FN0066

FN0067

FN0068

FN0070

FN0071

FN0072

FN0073

FN0074

FN0080

FN0081

FN0082

FN0084

FN0086

FN0088

FN0089

FN0090

FN0094

FN0096

FN0097

FN0098

FN0100

FN0102

FN0103

FN0104

FN0105

FN0106

FN0107

FN0108

FN0109

FN0110

FN0111

FN0112

FN0113

FN0114

FN0115

FN0116

FN0119

FN0124

FN0127

FN0133

FN0138

FN0139

FN0140

FN0141

FN0142

FN0143

FN0149

FN0150

FN0152

FN0155

FN0156

FN0157

FN0162

FN0164

FN0165

FN0178

FN0180

FN0181

FN0182

FN0183

FN0184

FN0186

FN0188

FN0193

FN0194

FN0195

FN0197

FN0198

FN0200

FN0201

FN0202

FN0203

FN0204

FN0206

FN0209

FN0210

FN0211

FN0212

FN0213

FN0214

FN0769

NCI_CGAP_Pr21

NCI_CGAP_Pr22

NCI_CGAP_Pr28

NIH_MGC_83

PN001-Normal-Human-Prostate

bvnorm

conorm

mynorm

yodnorm
