# Supplementary material for: A novel method of differential gene expression analysis using multiple cDNA libraries applied to the identification of tumour endothelial genes
Source: BMC Genomics. 2008 Apr 7;9:153. doi: 10.1186/1471-2164-9-153 (PMC2346479; doi:10.1186/1471-2164-9-153)
Supplement: Additional file 25 — 6 skin bulk tumour tissue libraries containing 12,484 ESTs were used versus skin normal libraries to find differentially expressed genes. [file 1471-2164-9-153-S25.doc]

**Additional file 25:** 6 skin bulk tumour tissue libraries containing 12,484 ESTs were used versus skin normal libraries to find differentially expressed genes.

Epithilioid sarcoma

NCI_CGAP_Mel1

NCI_CGAP_Mel15

NCI_CGAP_Mel3

NCI_CGAP_Skn4

Skin tumor I
