# Supplementary material for: A novel method of differential gene expression analysis using multiple cDNA libraries applied to the identification of tumour endothelial genes
Source: BMC Genomics. 2008 Apr 7;9:153. doi: 10.1186/1471-2164-9-153 (PMC2346479; doi:10.1186/1471-2164-9-153)
Supplement: Additional file 26 — 4 skin bulk normal tissue libraries containing 33,218 ESTs were used versus skin tumour libraries to find differentially expressed genes. [file 1471-2164-9-153-S26.doc]

**Additional file 26:** 4 skin bulk normal tissue libraries containing 33,218 ESTs were used versus skin tumour libraries to find differentially expressed genes.

NCI_CGAP_Skn1

NCI_CGAP_Skn3

Soares melanocyte 2NbHM
