# Supplementary material for: A novel method of differential gene expression analysis using multiple cDNA libraries applied to the identification of tumour endothelial genes
Source: BMC Genomics. 2008 Apr 7;9:153. doi: 10.1186/1471-2164-9-153 (PMC2346479; doi:10.1186/1471-2164-9-153)
Supplement: Additional file 27 — 557 colon bulk tumour tissue libraries containing 143,025 ESTs were used versus colon normal libraries to find differentially expressed genes. [file 1471-2164-9-153-S27.doc]

**Additional file 27:** 557 colon bulk tumour tissue libraries containing 143,025 ESTs were used versus colon normal libraries to find differentially expressed genes.

CT0001

CT0002

CT0003

CT0004

CT0005

CT0006

CT0008

CT0009

CT0010

CT0011

CT0012

CT0013

CT0014

CT0015

CT0016

CT0017

CT0018

CT0027

CT0029

CT0030

CT0031

CT0032

CT0033

CT0034

CT0035

CT0036

CT0037

CT0038

CT0039

CT0040

CT0041

CT0042

CT0043

CT0044

CT0045

CT0046

CT0047

CT0048

CT0050

CT0052

CT0054

CT0056

CT0057

CT0058

CT0059

CT0060

CT0061

CT0062

CT0063

CT0064

CT0065

CT0066

CT0068

CT0069

CT0070

CT0071

CT0075

CT0076

CT0077

CT0078

CT0079

CT0080

CT0081

CT0082

CT0083

CT0084

CT0086

CT0088

CT0090

CT0092

CT0094

CT0100

CT0101

CT0102

CT0103

CT0104

CT0105

CT0106

CT0109

CT0113

CT0114

CT0115

CT0116

CT0117

CT0121

CT0123

CT0127

CT0128

CT0129

CT0131

CT0135

CT0141

CT0143

CT0145

CT0147

CT0149

CT0151

CT0152

CT0153

CT0155

CT0157

CT0159

CT0160

CT0161

CT0163

CT0165

CT0167

CT0172

CT0173

CT0175

CT0176

CT0177

CT0178

CT0179

CT0180

CT0181

CT0186

CT0189

CT0192

CT0194

CT0195

CT0196

CT0197

CT0198

CT0199

CT0200

CT0201

CT0202

CT0203

CT0204

CT0205

CT0206

CT0207

CT0208

**Additional file 27:** Colon bulk tumour libraries

CT0209

CT0210

CT0212

CT0213

CT0214

CT0215

CT0216

CT0217

CT0219

CT0220

CT0221

CT0222

CT0223

CT0224

CT0225

CT0227

CT0236

CT0237

CT0238

CT0239

CT0240

CT0241

CT0242

CT0243

CT0244

CT0245

CT0246

CT0247

CT0248

CT0249

CT0250

CT0251

CT0252

CT0253

CT0254

CT0255

CT0256

CT0257

CT0258

CT0260

CT0261

CT0263

CT0264

CT0265

CT0266

CT0267

CT0268

CT0269

CT0275

CT0276

CT0277

CT0278

CT0279

CT0280

CT0281

CT0282

CT0283

CT0284

CT0285

CT0286

CT0287

CT0288

CT0289

CT0290

CT0291

CT0292

CT0293

CT0294

CT0295

CT0297

CT0298

CT0299

CT0300

CT0301

CT0302

CT0303

CT0304

CT0305

CT0306

CT0307

CT0308

CT0309

CT0310

CT0311

CT0312

CT0313

CT0314

CT0315

CT0317

CT0318

CT0319

CT0320

CT0321

CT0322

CT0323

CT0324

CT0325

CT0326

CT0328

CT0329

CT0330

CT0331

CT0333

CT0334

CT0336

CT0337

CT0338

CT0339

CT0341

CT0342

CT0343

CT0345

CT0347

CT0348

CT0349

CT0350

CT0352

CT0353

CT0354

CT0355

CT0357

CT0358

CT0360

CT0361

CT0363

CT0364

CT0365

CT0366

CT0367

CT0368

CT0369

CT0370

CT0372

CT0373

CT0376

CT0377

CT0378

CT0379

CT0380

CT0381

**Additional file 27:** Colon bulk tumour libraries

CT0382

CT0387

CT0386

CT0383

CT0388

CT0389

CT0390

CT0392

CT0393

CT0394

CT0395

CT0396

CT0397

CT0398

CT0400

CT0401

CT0402

CT0403

CT0404

CT0405

CT0406

CT0407

CT0408

CT0409

CT0411

CT0412

CT0413

CT0414

CT0415

CT0416

CT0417

CT0424

CT0425

CT0427

CT0428

CT0429

CT0432

CT0434

CT0437

CT0451

CT0452

CT0453

CT0454

CT0456

CT0458

CT0459

CT0461

CT0462

CT0463

CT0465

CT0466

CT0467

CT0469

CT0470

CT0471

CT0472

CT0473

CT0474

CT0477

CT0478

CT0479

CT0480

CT0481

CT0482

CT0483

CT0484

CT0485

CT0486

CT0487

CT0488

CT0491

CT0492

CT0497

CT0499

CT0500

CT0502

CT0505

CT0506

CT0507

CT0508

CT0510

CT0511

CT0512

CT0513

CT0514

CT0515

CT0516

CT0517

CT0518

CT0519

CT0520

CT0521

CT0522

CT0523

CT0524

CT0525

CT0527

CT0528

CT0529

CT0531

CT0532

CT0534

CT0537

CT0538

CT0539

CT0540

CT0541

CT0543

CT0545

CT0546

CT0547

CT0549

CT0550

CT0553

CT0554

CT0555

CT0556

CT0557

CT0558

CT0559

CT0560

CT0561

CT0562

CT0563

CT0564

CT0566

CT0570

CT0572

CT0574

CT0577

CT0581

CT0583

CT0586

CT0588

CT0589

CT0591

CT0592

CT0593

CT0594

**Additional file 27:** Colon bulk tumour libraries

CT0597

CT0598

CT0599

CT0600

CT0595

CT0596

CT0601

CT0602

CT0603

CT0605

CT0606

CT0607

CT0608

CT0616

CT0618

CT0621

CT0623

CT0624

CT0625

CT0626

CT0627

CT0628

CT0629

CT0631

CT0634

CT0635

CT0637

CT0638

CT0639

CT0640

CT0641

CT0642

CT0643

CT0644

CT0645

CT0648

CT0649

CT0651

CT0652

CT0653

CT0654

CT0655

CT0656

CT0657

CT0658

CT0659

CT0660

CT0661

CT0662

CT0663

CT0664

CT0669

CT0674

CT0676

CT0683

CT0685

CT0691

CT0692

CT0695

CT0697

CT0709

CT0710

CT0711

CT0714

CT0715

CT0716

CT0730

CT0731

CT0732

CT0734

CT0735

CT0736

CT0737

CT0741

CT0743

CT0744

CT0748

CT0749

CT0753

CT0754

CT0765

CT0772

CT0779

CT0780

CT0781

CT0782

CT0783

CT0793

CT0794

CT0795

CT0798

CT0799

CT0800

CT0801

CT0802

CT0803

CT0804

CT0805

CT0806

CT0807

CT0808

CT0809

CT0810

CT0811

CT0813

CT0814

CT0815

CT0817

CT0818

CT0819

CT0821

CT0823

CT0824

CT0829

CT0830

CT0839

CT0844

CT0845

CT0848

CT0849

CT0850

CT3001

CT3002

CT5001

CT5002

CT5003

Colon adenocarcinoma IV

Human colorectal cancer

NCI_CGAP_Co1

NCI_CGAP_Co10

NCI_CGAP_Co11

NCI_CGAP_Co12

NCI_CGAP_Co14

NCI_CGAP_Co16

NCI_CGAP_Co17

NCI_CGAP_Co18

NCI_CGAP_Co19

NCI_CGAP_Co20

NCI_CGAP_Co21

**Additional file 27:** Colon bulk tumour libraries

NCI_CGAP_Co3

NCI_CGAP_Co4

NCI_CGAP_Co8

NCI_CGAP_Co9

Stratagene colon HT29 (#937221)
