# Supplementary material for: A novel method of differential gene expression analysis using multiple cDNA libraries applied to the identification of tumour endothelial genes
Source: BMC Genomics. 2008 Apr 7;9:153. doi: 10.1186/1471-2164-9-153 (PMC2346479; doi:10.1186/1471-2164-9-153)
Supplement: Additional file 28 — 134 colon bulk normal tissue libraries containing 37,269 ESTs were used versus colon tumour libraries to find differentially expressed genes. [file 1471-2164-9-153-S28.doc]

**Additional file 28:** 134 colon bulk normal tissue libraries containing 37,269 ESTs were used versus colon tumour libraries to find differentially expressed genes.

Barstead colon HPLRB7

CN0002

CN0003

CN0004

CN0005

CN0006

CN0007

CN0008

CN0009

CN0010

CN0011

CN0012

CN0013

CN0014

CN0016

CN0017

CN0018

CN0019

CN0023

CN0024

CN0025

CN0026

CN0027

CN0028

CN0029

CN0030

CN0031

CN0032

CN0033

CN0035

CN0036

CN0037

CN0038

CN0039

CN0040

CN0041

CN0042

CN0043

CN0044

CN0045

CN0046

CN0048

CN0049

CN0050

CN0051

CN0053

CN0054

CN0055

CN0056

CN0057

CN0060

CN0061

CN0062

CN0063

CN0065

CN0067

CN0068

CN0076

CN0077

CN0078

CN0080

CN0081

CN0082

CN0083

CN0084

CN0085

CN0089

CN0090

CN0091

CN0092

CN0093

CN0094

CN0095

CN0096

CN0097

CN0098

CN0099

CN0100

CN0101

CN0104

CN0105

CN0109

CN0110

CN0112

CN0114

CN0115

CN0120

CN0129

CN0136

CN0137

CN0138

CN0147

CN0150

CN0151

CN0152

CN0153

CN0155

CN0156

CN0157

CN0159

CN0160

CN0161

CN0162

CN0163

CN0164

CN0165

CN0168

CN0170

CN0171

CN0173

CN0174

CN0175

CN0177

CN0185

CN0186

CN0188

CN0190

CN0191

CN0192

CN0193

CN0194

CN0195

CN0202

CN0203

CN0204

CN0265

CN0344

CN0513

CN0612

CN0833

Colon I

Human Colon

Human colon mucosa

NIH_MGC_116
