# Supplementary material for: A novel method of differential gene expression analysis using multiple cDNA libraries applied to the identification of tumour endothelial genes
Source: BMC Genomics. 2008 Apr 7;9:153. doi: 10.1186/1471-2164-9-153 (PMC2346479; doi:10.1186/1471-2164-9-153)
Supplement: Additional file 29 — Potential Tumour Endothelial Markers and their likelihood ratio test statistic results. [file 1471-2164-9-153-S29.doc]

## Additional File 29: Potential Tumour Endothelial Markers

27 predicted TEMs that were significantly endothelial were also up-regulated or specific to tumours. Three foetal tissues were also screened as they contain regions of active angiogenesis. The information held in each cell is as follows:

E.g. UP 0.21 3 0 (space delimited),

UP/DOWN = gene was up or down regulated in the tissue

0.21 = FDR q-value

3 0 = EST counts, 3 tumour bulk tissue ESTs and 0 normal bulk tissue ESTs.

Red text are genes showing tumour or foetal specific expression (no counts in normal tissue) but not at a statistically significant level.

Blue text denotes genes that showed statistically significant (q-value <= 0.01) differential expression but not specific (some expression seen in normal tissue). Green values portray significant and specific results.

Green text is for genes that were both significantly (q-value <= 0.01) and specifically (no counts in normal tissue) up regulated in tumour or foetal tissue.

| **Gene** | **brain** | **colon** | **kidney** | **Lung** | **skin** | **prostate** | **Foetal kidney** | **Foetal lung** | **Foetal brain** |
| --- | --- | --- | --- | --- | --- | --- | --- | --- | --- |
| **SPHK1** | DOWN 0.86 1 1 | **UP 0.57 2 0** | **UP 0.38 1 0** | **UP 0.33 2 0** | **UP 0.22 2 0** | - - | - - | **UP 0.43 1 0** | UP 0.83 1 1 |
| **KCTD15** | **UP 0.33 2 0** | **UP 0.62 1 0** | DOWN 0.42 0 2 | **UP 0.08 6 0** | UP 0.45 3 3 | - - | DOWN 0.79 0 2 | **UP 0.01 13 0** | **UP 0.21 2 0** |
| **LRRC8C** | **UP 0.33 2 0** | **UP 0.62 1 0** | - - | - - | - - | **UP 0.41 1 0** | - - | **UP 0.33 2 0** | - - |
| **PCDH12** | **UP 0.43 1 0** | **UP 0.52 3 0** | **UP 0.38 1 0** | - - | - - | - - | - - | **UP 0.23 3 0** | - - |
| **SPARC** | **UP 0.00 155 31** | **UP 0.01 82 6** | **UP 0.00 37 8** | DOWN 0.18 21 29 | UP 0.71 12 27 | **UP 0.00 32 25** | **UP 0.00 6 8** | UP 0.44 50 29 | UP 0.44 28 31 |
| **ANGPT2** | - - | **UP 0.52 3 0** | **UP 0.13 3 0** | - - | - - | - - | - - | **UP 0.43 1 0** | - - |
| **VIM** | **UP 0.00 323 9** | DOWN 0.75 36 11 | **UP 0.00 35 9** | UP 0.87 50 36 | **UP 0.00 23 11** | DOWN 0.70 2 10 | UP 0.07 4 9 | DOWN 0.53 40 36 | **UP 0.00 94 9** |
| **BGN** | **UP 0.00 61 4** | UP 0.95 28 7 | UP 0.87 2 3 | UP 0.14 21 6 | **UP 0.00 7 0** | UP 0.55 1 1 | DOWN 0.79 0 3 | DOWN 0.85 7 6 | DOWN 0.81 2 4 |
| **C12orf11** | **UP 0.11 5 0** | **UP 0.46 4 0** | DOWN 0.63 1 4 | DOWN 0.37 0 1 | **UP 0.31 1 0** | **UP 0.41 1 0** | DOWN 0.79 0 4 | UP 0.84 2 1 | **UP 0.21 2 0** |
| **C16orf30** | **UP 0.07 6 0** | **UP 0.40 5 0** | DOWN 0.20 1 12 | DOWN 0.37 0 1 | - - | DOWN 0.54 0 2 | UP 0.79 1 12 | UP 0.04 13 1 | - - |
| **ECOP** | **UP 0.00 27 0** | **UP 0.46 4 0** | DOWN 0.37 0 3 | UP 0.59 3 1 | UP 0.85 1 2 | DOWN 0.55 0 1 | DOWN 0.79 0 3 | UP 0.84 2 1 | - - |
| **ECSM2** | **UP 0.16 4 0** | **UP 0.46 4 0** | - - | UP 0.59 3 1 | - - | UP 0.70 1 2 | - - | **UP 0.23 3 0** | - - |
| **ERG** | - - | - - | DOWN 0.77 1 3 | **UP 0.33 2 0** | - - | **UP 0.41 1 0** | DOWN 0.79 0 3 | **UP 0.04 8 0** | **UP 0.34 1 0** |
| **GBP4** | **UP 0.43 1 0** | **UP 0.62 1 0** | **UP 0.03 5 0** | UP 0.37 7 2 | **UP 0.31 1 0** | DOWN 0.54 0 2 | - - | DOWN 0.23 0 2 | - - |
| **IKBKE** | **UP 0.07 6 0** | **UP 0.57 2 0** | **UP 0.38 1 0** | DOWN 0.59 2 3 | - - | - - | - - | UP 0.71 6 3 | - - |
| **LOC653949** | **UP 0.07 6 0** | UP 0.57 10 1 | **UP 0.00 32 0** | DOWN 0.37 3 6 | DOWN 0.53 0 1 | DOWN 0.55 1 8 | - - | DOWN 0.43 4 6 | **UP 0.34 1 0** |
| **MED28** | **UP 0.11 5 0** | **UP 0.46 4 0** | UP 0.77 1 1 | **UP 0.22 3 0** | DOWN 0.53 0 1 | - - | DOWN 0.79 0 1 | **UP 0.18 4 0** | **UP 0.21 2 0** |
| **P4HB** | **UP 0.00 49 2** | UP 0.62 50 10 | **UP 0.01 13 3** | **UP 0.01 42 10** | DOWN 0.22 1 16 | UP 0.79 5 15 | DOWN 0.79 0 3 | DOWN 0.43 8 10 | **UP 0.00 33 2** |
| **PLOD3** | **UP 0.00 19 0** | **UP 0.16 13 0** | UP 0.37 4 2 | **UP 0.08 6 0** | UP 0.37 2 1 | UP 0.51 3 4 | UP 0.79 1 2 | **UP 0.00 14 0** | **UP 0.21 2 0** |
| **PTTG1IP** | **UP 0.01 25 3** | **UP 0.00 60 1** | **UP 0.01 18 8** | UP 0.89 25 18 | DOWN 0.37 3 18 | DOWN 0.44 2 20 | UP 0.05 4 8 | DOWN 0.58 19 18 | UP 0.33 6 3 |
| **SERP1** | **UP 0.01 34 7** | **UP 0.00 90 3** | UP 0.37 13 13 | UP 0.33 16 6 | DOWN 0.13 0 13 | DOWN 0.55 2 12 | DOWN 0.79 0 13 | **UP 0.01 34 6** | UP 0.97 5 7 |
| **STAB1** | **UP 0.00 32 1** | DOWN 0.49 2 2 | **UP 0.23 2 0** | **UP 0.22 3 0** | - - | DOWN 0.55 0 1 | - - | **UP 0.43 1 0** | DOWN 0.43 0 1 |
| **THRAP4** | **UP 0.00 21 0** | DOWN 0.85 6 2 | DOWN 0.30 0 4 | UP 0.90 3 2 | **UP 0.01 6 0** | **UP 0.41 1 0** | UP 0.79 1 4 | DOWN 0.84 2 2 | **UP 0.06 4 0** |
| **TXNDC5** | UP 0.05 12 1 | **UP 0.00 62 2** | **UP 0.00 10 1** | DOWN 0.42 21 23 | UP 0.31 4 3 | UP 0.48 3 3 | DOWN 0.79 0 1 | DOWN 0.04 11 23 | UP 0.34 3 1 |
| **APLN** | **UP 0.00 13 0** | **UP 0.57 2 0** | DOWN 0.20 0 6 | - - | - - | - - | DOWN 0.79 0 6 | **UP 0.33 2 0** | - - |
| **ROBO4** | **UP 0.21 3 0** | DOWN 0.34 1 2 | **UP 0.38 1 0** | DOWN 0.57 1 2 | DOWN 0.53 0 1 | - - | **UP 0.32 1 0** | UP 0.33 8 2 | **UP 0.02 6 0** |
