# Supplementary figures and images for: A novel method of differential gene expression analysis using multiple cDNA libraries applied to the identification of tumour endothelial genes
Source: BMC Genomics. 2008 Apr 7;9:153. doi: 10.1186/1471-2164-9-153 (PMC2346479; doi:10.1186/1471-2164-9-153)

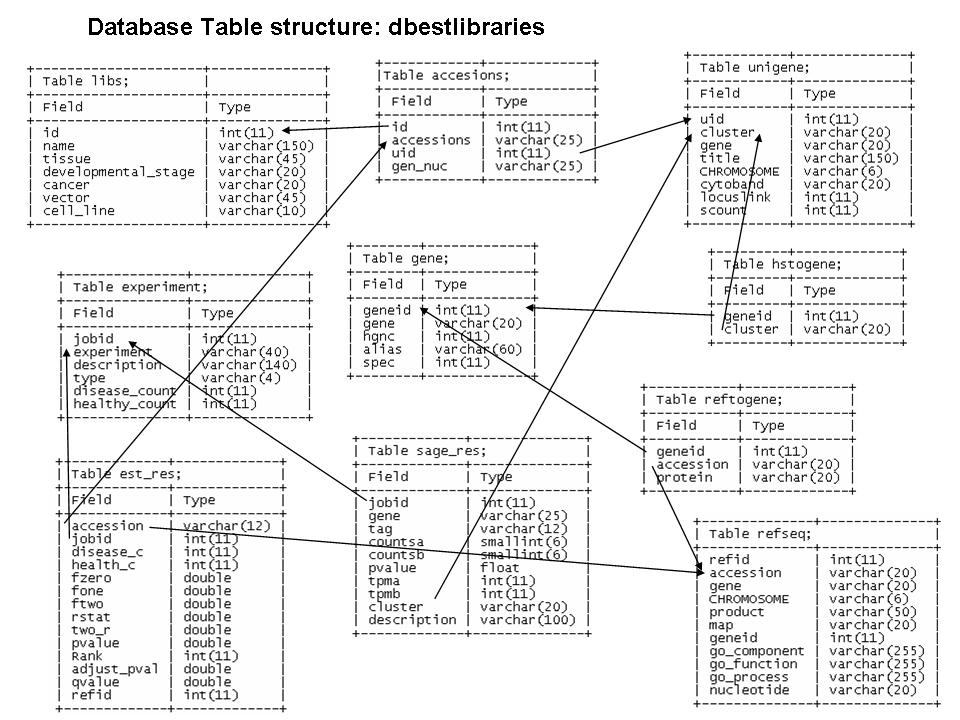

Supplement: Additional file 37 — A schema of the database constructed to store cDNA library data, gene/Refseq annotations and cDNA/SAGE library analysis results. [file 1471-2164-9-153-S37.jpeg]
