# Supplementary material for: A novel method of differential gene expression analysis using multiple cDNA libraries applied to the identification of tumour endothelial genes
Source: BMC Genomics. 2008 Apr 7;9:153. doi: 10.1186/1471-2164-9-153 (PMC2346479; doi:10.1186/1471-2164-9-153)
Supplement: Additional file 38 — Real Time PCR was carried out to find preferential expression of genes amongst several cell isolates. This lists the primers used for each of the genes. [file 1471-2164-9-153-S38.doc]

**Additional File 38:** Real Time PCR was carried out to find preferential expression of genes amongst several cell isolates. This lists the primers used for each of the genes.

| **Gene** | **Accession Number** | **Forward Primer** | **Reverse Primer** | **Probe Number** |
| --- | --- | --- | --- | --- |
| *FLOT2* | NM_004475 | ctcagcttcaccatcaaggac | tcagcatctctctgcaccac | 61 |
| *Ubiquitin C* | NM_021009 | cctgaccagcagaggttgat | tgttgtagtcagacagggtacga | 11 |
| *β-Actin* | NM_001101 | cccagcacaatgaagatcaa | cgatccacacggagtacttg | 63 |
| *ECSM2* | NM_001077693 | cacagagccagtttcttcca | gcttgtgcctccgtctctt | 03 |
| *MMP1* | NM_002421 | gatgaagtccggtttttcaaag | ggggtatccgtgtagcacat | 26 |
| *SOX18* | NM_018419 | atggtgtgggcaaaggac | gcgttcagctccttccac | 61 |
| *ERG* | NM_004449 | aagtagccgccttgcaaat | gctggagttggagctgtcc | 79 |
| *RHOJ* | NM_020663 | aaaccctgcctcttaccaca | catcacggagatcaatctgg | 79 |
| *APLN* | NM_017413 | gctctggctctccttgacc | ccattccttgaccctctgg | 11 |
| *MMRN2* | NM_024756 | gacacctggggtctggaag | ggtaggggcaccagttacg | 73 |
| *STAB1* | NM_015136 | tctttgtgcccaccaacc | gcactgtgtctgcgtcca | 37 |
| *LYL1* | NM_005583 | caggctgcaagaacagtgc | ctgccttctcagtcatggtg | 01 |
| *ELTD1* | XM_371262 | tccaaaagaccacagagtttga | tgcagcttttctctttggaaat | 58 |
| *EFEMP1* | NM_001039348 | aacccttcccaccgtatcc | tgcagtgcactcgtctatgtc | 40 |
| *BMX* | NM_001721 | ggaattccctgagtcaagttca | gatgttaccagcaaaccagtca | 35 |
